# Supplementary material for: Low-dose metformin targets the lysosomal AMPK pathway through PEN2
Source: Nature. 2022 Feb 23;603(7899):159–65. doi: 10.1038/s41586-022-04431-8 (PMC8891018; doi:10.1038/s41586-022-04431-8)

nature portfolio

https://doi.org/10.1038/s41586-022-04431-8

Supplementary information

Low-dose metformin targets the lysosomal AMPK pathway through PEN2

In the format provided by the authors and unedited

Supplementary Information for

Low-dose metformin targets the lysosome-AMPK pathway through PEN2

Teng Ma,[1,6] Xiao Tian,[1,6] Baoding Zhang,[1,6] Mengqi Li,[1] Yu Wang,[1] Chunyan Yang,[1] Jianfeng Wu,[2] Xiaoyan Wei,[1] Qi Qu,[1] Yaxin Yu,[1] Shating Long,[1] Jin-Wei Feng,[1] Chun Li,[1] Cixiong Zhang,[1] Changchuan Xie,[1] Yaying Wu,[1] Zheni Xu,[1] Junjie Chen,[3] Yong Yu,[1] Xi Huang,[1] Ying He,[1] Luming Yao,[1] Lei Zhang,[1] Mingxia Zhu,[1] Wen Wang,[4] Zhi-Chao Wang,[4] Mingliang Zhang,[5] Yuqian Bao,[5] Weiping Jia,[5] Shu-Yong Lin,[1] Zhiyun Ye,[1] Hai-Long Piao,[4] Xianming Deng,[1,7] Chen-Song Zhang,[1,7] and Sheng-Cai Lin[1,7]

[1]State Key Laboratory for Cellular Stress Biology, Innovation Centre for Cell Signalling Network, School of Life Sciences, Xiamen University, Fujian 361102, China.

[2]Laboratory Animal Research Centre, Xiamen University, Fujian 361102, China.

[3]Analysis and Measurement Centre, School of Pharmaceutical Sciences, Xiamen University, Fujian 361102, China.

[4]CAS Key Laboratory of Separation Science for Analytical Chemistry, Dalian Institute of Chemical Physics, Chinese Academy of Sciences, Liaoning 116023, China.

[5]Department of Endocrinology and Metabolism, Shanghai Clinical Centre for Diabetes, Shanghai Diabetes Institute, Shanghai Key Laboratory of Diabetes Mellitus, Shanghai Jiao Tong University Affiliated Sixth People's Hospital, Shanghai 200233, China.

[6]These authors contributed equally to this work.

[7]e-mail: xmdeng@xmu.edu.cn; cszhang@xmu.edu.cn; linsc@xmu.edu.cn

This file includes:

Supplementary Notes 1 to 10 (Pages 3 to 9)

References for Supplementary Notes (ref. 64 to 116, Pages 9 to 13)

Supplementary Fig. 1 (gel source data, Pages 14 to 45)

Supplementary Fig. 2 (NMR source data for Met-Ps, Page 46)

Supplementary Note 1

We have previously shown that metformin inhibits the vacuolar H^+-ATPase (v-ATPase) complex, triggering lysosomal translocation of AXIN and LKB1 to form the Ragulator-v-ATPase-AXIN/LKB1-AMPK super-complex, leading to AMPK activation[24,64,65]. As shown in Fig. 1a, b and Extended Data Fig. 1b, c, we found that metformin at concentrations as low as 5 μM could robustly inactivate v-ATPase and activate AMPK in human or mouse primary hepatocytes, as determined by the signal from LysoSensor Green DND-189 dye (which decreases when lysosomal pH increases) and by increased phosphorylation of Thr172 on AMPKα and Ser79/Ser212 on ACC1/2. Since it has been suggested that the intracellular concentrations of metformin might be higher than the plasma concentrations due to possible cumulative effect, we also measured the activity of v-ATPase in cell-free assays using purified lysosomes, and found that 5 μM metformin was also sufficient to inhibit v-ATPase, as evidenced by decreases of ATP hydrolysis and proton transport activity (Extended Data Fig. 1d). Importantly, the cellular AMP:ATP and ADP:ATP ratios, absolute concentrations of AMP, ADP and ATP, as well as mitochondrial membrane potentials and mitochondrial oxygen consumption rates (OCR) were unchanged in metformin-treated cells, even after 48 h (Extended Data Fig. 1e-g). We also administered mice with a series of doses of metformin, and detected an average concentration of 15 μM metformin in the blood of mice taking metformin from drinking water at 1 g/l, and activation of AMPK in the liver in which the AMP:ATP and ADP:ATP ratios remained unchanged (Extended Data Fig. 1h-n). By contrast, in MEFs and HEK293T cells that lack the metformin transporter OCT1, we found that treatment with 200 μM or 300 μM metformin for 12 h led to intracellular metformin concentrations similar to those detected in the livers of mice administered with metformin in drinking water (Extended Data Fig. 1o, p). Under these conditions, while the v-ATPase was effectively inhibited, no elevation of AMP:ATP or ADP:ATP ratios, or disruption of mitochondrial membrane potentials and OCR, was detected (Extended Data Fig. 1q-t). Metformin in mitochondria was found to be at a similar concentration to that in the cytosol in MEFs (Extended Data Fig. 1u), indicative of no mitochondrial accumulation of metformin. To avoid possible side effects caused by the 12-h incubation of metformin in MEFs and HEK293T cells, we established MEFs and HEK293T cells with OCT1 stably expressed, and found that incubation for 2 h of metformin (5 μM) led to 34 μM and 35 μM metformin in MEFs and HEK293T cells, respectively, which are similar to those in primary hepatocytes (Extended Data Fig. 1v, w). Similar AMPK activation and unchanged AMP:ATP ratios were detected in the OCT1-expressing MEFs and HEK293T cells (Extended Data Fig. 1v, w). Therefore, clinical doses of metformin can activate AMPK without altering the levels of AMP or ADP.

Of note, the intracellular metformin concentrations in cultured mouse primary hepatocytes were about 5 folds higher than that in the culture medium (Fig. 1b), consistent with the previous findings[66,67]. However, metformin concentrations in the liver from mice administered with 1 g/l metformin in drinking water were similar to

those in the plasma (Extended Data Fig. 1h). One possible explanation is that the secretion/elimination rates of hepatic metformin inside animals, driven by relatively low concentrations of metformin in hepatic vein and high capability of renal OCT2 transporter in mouse models, are very high, 3 folds higher than the maximal hepatic metformin uptake rate[68]. This situation might be more pronounced in our mouse model that is treated with low metformin: a stable (at least 1 week) treatment with low metformin (1 g/l in drinking water). Such a high capacity of metformin clearance has been supported by some pharmacokinetic studies. For example, in short time durations (within 120 min) after intravenous injection (i.v.) of metformin, the plasma metformin concentrations were not dose-proportional, only with a narrow change between the 50 mg/kg (mpk) and the 100 mpk groups, and were increased only at 200 mpk[69]. Given that the plasma metformin achieved in our model is far below that of i.v. at 50 mpk within 120 min, it is reasonable to suggest that metformin is rapidly eliminated in the liver and may therefore be equally distributed between liver and plasma.

Supplementary Note 2

We identified that, after subtracting hits from the DMSO control group, 3,042 proteins with at least one specific peptide hit from the mass spectrometry. Among these proteins, 219 had been listed as lysosomal resident proteins[70], and 58 had been shown to be related with lysosome by us and others. To avoid possible omission during the mass spectrometry analysis due to low expression levels or poor digestion by trypsin, we also tested metformin probe interactions with an additional 90 proteins from the list of lysosomal proteins[70]. Therefore, a total of 367 proteins were chosen for the verification, through which we identified PEN2 as a metformin-interacting protein that may participate in the activation of AMPK by low metformin.

Interestingly, although *PEN2* knockout blocked the activation of AMPK, as well as the inhibition of v-ATPase, in low metformin in primary hepatocytes, MEFs or HEK293T cells, we found that *PEN2* is not required for maintaining the basal activity of v-ATPase (e.g., Extended Data Fig. 3a, c, i). Similarly, it has been shown that lysosomes in MEFs with PS1/2-DKO show intact pH[71]. However, in neuronal cells, it has been shown that PS1 is strictly required for maintaining lysosomal acidity[72]. Therefore, effects of γ-secretase on lysosomal acidity might be cell type-specific.

Supplementary Note 3

PEN2 was originally identified as a component of the γ-secretase, an aspartyl intramembrane protease that catalyses the cleavage of a wide spectrum of type I integral membrane proteins including the precursor protein for beta-amyloid involved in the Alzheimer's disease[7,73,74] (see also the cryo-electron microscopy structure, PDB ID: 6IYC[75]). In addition to PEN2, the γ-secretase comprises other subunits, including presenilin-1/2 (PS1/2, the catalytic subunit), nicastrin (NCSTN) and APH1A/B (or APH1A/B/C in mouse)[74,76]. We found that the activity of the γ-secretase holoenzyme

may be dispensable for the regulation of AMPK by metformin, because a triple knockout of *APH1A*/*B*/*C* in MEFs did not affect the activation of AMPK (Extended Data Fig. 3n). Consistent with this, treating MEFs with two γ-secretase inhibitors, DAPT[77] or RO4929097 (ref. [78]) did not affect metformin-induced AMPK activation (Extended Data Fig. 3m). By comparison, knockout of *NCSTN* dampened AMPK activation upon metformin treatment, possibly because NCSTN is required for maintaining the protein stability of PEN2 (ref. [79] and Extended Data Fig. 3p, in which the protein levels of PEN2 was decreased by approximately 60%; see also Extended Data Fig. 3r, o for the *NCSTN* knockout strategy). Indeed, re-introduction of PEN2 in *NCSTN*$^{-/-}$ MEFs restored AMPK activation (Extended Data Fig. 3q, see validation data in Extended Data Fig. 3r). Although PS1 and PS2 have been shown to be also required for PEN2 stability[80,81], double knockout of *PS1* and *PS2* in MEFs caused a much milder reduction of PEN2 (about a 15% decrease, Extended Data Fig. 3s, and 4a, b), and did not affect metformin-induced AMPK activation.

The dispensability of γ-secretase activity for the AMPK activation under metformin treatment was further confirmed by the effects of the two PEN2 mutants, PEN2-2A and PEN2-20A. Although both PEN2-2A and PEN2-20A blocked metformin-induced AMPK activation, PEN2-2A was found to even slightly increase the activity of γ-secretase, as determined by the cleavage of Notch protein[82], while PEN2-20A blocked the activity of γ-secretase (Extended Data Fig. 6m).

Interestingly, although we have shown that the activity of γ-secretase is not required for the activation of AMPK under metformin treatment, metformin could slightly increase the activity of γ-secretase (Extended Data Fig. 6o) without altering the complex formation of γ-secretase (Extended Data Fig. 6n). In addition, one previous study reported that metformin promotes the γ-secretase-mediated production of Aβ42, as a result of autophagy induction in Tg6799 AD model mice[83], although (possibly owing to different mouse models used) another study found the opposite, showing that that metformin prevents amyloid plaque deposition and memory impairment in APP/PS1 mice[84]. Therefore, whether and how metformin affects neuronal degeneration awaits further investigation.

As to the relationship between γ-secretase and ATP6AP1, we found that ATP6AP1 showed some basal interaction with NCSTN, presenilins and APH1 (Extended Data Fig. 7f), consistent with the previous studies showing that γ-secretase can interact with the v-ATPase complex[85,86]. We further showed that these interactions were promoted by metformin, and importantly were dependent on PEN2 (Extended Data Fig. 7f, g). Of note, ATP6AP1 itself did not bind Met-P1 (Extended Data Fig. 7h). These results therefore point to the conclusion that lysosomal PEN2 is recruited to ATP6AP1 of the v-ATPase complex by binding of metformin to the former.

Supplementary Note 4

The observation that a portion of PEN2 is localised on the lysosome is consistent with previous findings that PEN2 is widely distributed across the cell, including the lysosome, ER and plasma membrane[87]. At this point, we really have no idea through which mechanism PEN2 is targeted to the lysosome, because no apparent lysosome-targeting sequence is found on PEN2. It appears that autophagy is not involved in mediating the lysosomal localisation of PEN2: knockout of *ATG5*, or treatment of chloroquine, 3-MA or bafilomycin A, did not affect the localisation of PEN2 (Extended Data Fig. 4f). Similarly, inhibitors of endocytosis, such as Dynasore and Dyngo-4a which inhibit the dynamin-mediated endocytosis (including the clathrin-coated, pit-mediated endocytosis, and the fast endophilin-mediated endocytosis), Nystatin and methyl-β-cyclodextrin which inhibit the clathrin-independent carrier/glycosylphosphatidylinositol-anchored protein-enriched early endocytic compartment endocytosis and the caveolae-dependent endocytosis, and cytochalasin D which inhibits macropinocytosis and phagocytosis, all failed to disrupt lysosomal localisation of PEN2 (Extended Data Fig. 4g). As for the roles of other γ-secretase subunits, we found that knockout of *PS1* and *PS2*, *NCSTN* and *APH1A*/*B*/*C*, all failed to affect the lysosomal localisation of PEN2 (Extended Data Fig. 5a). Therefore, how PEN2 is targeted to the lysosome remains unclear.

Supplementary Note 5

We chose lysosomal protein extracts to identify potential targets of metformin, because we found that metformin was able to inhibit v-ATPase in purified lysosomes (e.g., Extended Data Fig. 1d), which suggested that the lysosomal proteins contain mediators that can signal to inhibit v-ATPase to prime AMPK activation. It was also because we had previously found that knockout of *AXIN* or *LAMTOR1*, factors of the lysosomal pathway, abolished metformin-induced AMPK activation[64], which was recapitulated in this study (Extended Data Fig. 9a, b). Similarly, re-introduction of AMPKβ1-G2A mutant that cannot be localised to lysosome[88], into AMPKβ1/2-DKO MEFs, did not mediate the activation of AMPK by low metformin (Extended Data Fig. 9h). In the subsequent verification processes following the mass analysis on the Met-P1:protein conjugates, we started with 219 such identified proteins, and 58 more proteins that had been shown to be related to the lysosome, either according to our mass spectrometry determination of the total proteins in the purified lysosomes or by others in the new literatures. We even went further to include 90 additional proteins in the list of "lysosomal proteins[70]" that were not among the hits from the Met-P1 conjugates, because some proteins, possibly owing to the low expression levels or poor digestion by trypsin, might have been missed by mass spectrometry. This resulted in a total of 367 proteins to undergo subsequent verification processes. After gene silencing experiments, we found that knockdown, as well as all the other approaches, of PEN2, but not the others, abolished metformin signalling in MEFs based initially on AMPK activation. We have also shown that it is PEN2 that physically binds to metformin. However, we could not exclude the possibility that other cytosolic regulatory protein(s) may also translocate to the lysosome and participate in metformin signalling: AXIN,

cytosolically localised, is one of such examples.

Supplementary Note 6

The K_D values of PEN2 for metformin were determined to be 1.7 µM and 0.15 µM by ITC and BIAcore, respectively. Similarly, we found that metformin at 5 µM sufficiently inhibited the activity of v-ATPase *in vitro* (e.g., Extended Data Fig. 1d), and that 5 µM metformin could promote the interactions between PEN2 and ATP6AP1 in both cell lysates and *in vitro* (in purified proteins). However, intracellular concentrations of metformin were estimated to be over 10 µM for AMPK activation in the various cells: hepatocytes at 25 µM of metformin, MEFs at 15 µM, and HEK293T cells at 20 µM (Fig. 1b, and Extended Data Fig. 1o, p). One possible explanation for a higher intracellular concentration than the K_D values may be that diffusion for the monoprotonated metformin to the lysosome may be hindered by its positive charge, such that there needs to be presence of higher intracellular concentrations of metformin. Evidence in support of this is that metformin can be immobilised by copper and iron ions in cells[89-91]. In addition, the cytosolic face of the lysosome tends to be positively charged, adding a possible hindrance of metformin from reaching to the lysosomal surface[92].

Supplementary Note 7

Although we and others[20] have shown that low metformin could reduce severity of fatty liver on HFD-treated mice, systematic reviews and meta-analysis on patients concluded that metformin did not have a substantial impact on liver disease[93,94]. The ineffectiveness observed in human patients might be attributed to the fact that the time duration for taking metformin is not long enough or punctuated. In particular, we found that 4 months' administration of low metformin was required for reducing hepatic fat in mice. Requirement of such a long duration may be explained by: a) a mild promotion of β-oxidation by low metformin, as determined by the conversion rates of [U-^{13}C]-palmitate to the intermediates of the TCA cycle (Extended Data Fig. 13g); b) no effect on TAG synthesis from fatty acids by low metformin, as determined by the conversion rates of [U-^{13}C]-palmitate to TAG (Extended Data Fig. 13h). Furthermore, although low metformin efficiently inhibited the de novo lipogenesis, as determined by the conversion rates of [U-^{13}C]-glucose to TAG (Extended Data Fig. 13h), the rates of de novo lipogenesis is intrinsically low, particularly in HFD-fed mice in which the de novo lipogenesis is strongly supressed by the abundant, exogenous fatty acids. Moreover, the ineffectiveness of fatty liver alleviation in patients might also be caused by the nature of the development of fatty liver among different individuals: if the fatty liver is not caused by over-eating like that of HFD-treated mice, but is caused by other reasons such as different genetic backgrounds, metformin may not have an effect.

Supplementary Note 8

It has been shown that autophagy plays critical roles in mediating many beneficial effects of AMPK, including improvement of hepatic fat-related decreases and glucose tolerance, and lifespan extension (reviewed in ref. [95]). Since we have shown that low metformin inhibits the activity of v-ATPase and hence decreases lysosomal acidity, it is interesting to determine whether autophagy could still happen under metformin treatment. We found that in MEFs, the increase of lysosomal pH, and the increase of p-AMPK signals emerged after 8 h of metformin treatment and p-AMPK signals remained relatively constant up to 72 h. The decrease of p62, as autophagy indicators, however, only became obvious after 30 h of metformin treatment, accompanied with a recovery of overall lysosomal acidity as assessed by Lysosensor (Extended Data Fig. 14a, b). Therefore, v-ATPase inhibition and AMPK activation seem to occur much earlier than autophagy induction. Even though v-ATPase was inhibited to elevate the lysosomal pH at an early stage of metformin treatment, it did not impair the initiation of autophagy at later stages. It remains intriguing as to if the same lysosomes undergo autophagy as those inhibited of the v-ATPase.

Supplementary Note 9

Another important downstream event of AMPK is to promote the production of the reactive oxygen species (ROS)[96,97]. It has been shown that metformin can increase ROS production in both mammalian cells (e.g., in 3T3-L1 cells treated with 4 mM metformin[98]) and in *C. elegans*[99]. We also found that in nematodes, metformin increased ROS by approximately 37% on average, which could be blocked by N-acetyl cysteine (NAC) (Extended Data Fig. 14c). We also observed that metformin-mediated lifespan extension of *C. elegans* was blocked by NAC (Extended Data Fig. 14d), as shown previously[96,99]. Of note, no elevation of AMP:ATP and ADP:ATP ratios was observed in *C. elegans* (Extended Data Fig. 13b, f), indicating that metformin at such a concentration is not sufficient to cause mitochondrial damage. Consistently, one recent study suggests that the effects of metformin on lifespan extension can be achieved only when the energy states are not altered[36]. On the contrary, it was shown that if AMP:ATP and ADP:ATP ratios are elevated by metformin (e.g., administered to aged nematodes), the nematode lifespan is even shortened despite the elevation of ROS[36].

It has also been suggested that ROS modulates AMPK activity (e.g., ref. [14]). In MEFs, we failed to detect any increase of ROS in low metformin (Extended Data Fig. 14f), suggesting that metformin may only increase ROS at high concentrations. Consistently, pre-treatment of NAC (24 h) on MEFs, HEK293T cells or primary hepatocytes did not alter the activity of AMPK under low metformin (Extended Data Fig. 14g, h). Similarly, NAC did not alter the activity of AMPK in *C. elegans* (Extended Data Fig. 14i), although metformin did elevate ROS in the nematodes. Therefore, it is reasonable to suggest that low metformin activates AMPK in an ROS-independent manner. Such a conclusion may also be supported by the previous reports that ROS could only activate AMPK in a condition where mitochondria were severely damaged and AMP levels were elevated[100,101].

Supplementary Note 10

Since the activity of v-ATPase exerts a critical role in modulating the activity of mTORC1[102], we also examined mTORC1 signalling in PEN2-KO MEFs/HEK293T cells, and ATP6AP1-KO MEFs/HEK293T cells re-introduced with ATP6AP1 or its Δ420-440 treated with metformin. We found that while metformin treatment reduced levels of phosphorylated S6K (a substrate of mTORC1), the deficiency of the PEN2-ATP6AP1 axis rendered low metformin unable to dampen mTORC1, indicating that the PEN2-ATP6AP1 axis is required for metformin to inhibit mTORC1, through inhibiting the v-ATPase (Extended Data Fig. 14l, m). The v-ATPase is thus the converging point for both metformin and low glucose, to regulate both AMPK activation and mTORC1 inhibition, with the former targeting ATP6AP1 and the latter changing the conformation of aldolase[25], both of which are integral components of the v-ATPase complex[103-108].

However, mTORC1 does not seem to be involved in the (AMPK-mediated) beneficial effects of metformin, on the physiological processes investigated in this study. For the roles of mTORC1 (inhibition) in promoting post-prandial glucose absorption, controversial results have been shown. Although the dominant-negative form of mTOR expressed in duodenum is able to promote glucose absorption[109], many other studies have shown that inhibition of mTORC1 by short-term rapamycin treatment even impairs glucose tolerance (e.g., ref. [110]). Therefore, roles of mTORC1 in glucose tolerance may be context-dependent. As for the roles of mTORC1 in hepatic TAG reduction, it has been shown that hepatic depletion of *Raptor*, which leads to a constitutive inactivation of mTORC1, did not affect HFD-induced fatty liver in mice[111]. Although long-term rapamycin treatment can alleviate fatty liver[112], such an effect was later attributed to the inhibition of mTORC2[113], given that liver-specific knockout of *Rictor*, which renders mTORC2 inactivated blocks the HFD-induced hepatic TAG accumulation[114]. Therefore, inhibition of mTORC1 unlikely plays a significant role in the fatty liver alleviation. Finally, although knockdown of *Raptor* (*daf-15*), or treatment of rapamycin has also been shown to extend lifespan in *C. elegans*[115], metformin could still extend lifespan in the *Raptor* deficient nematodes[116], suggesting that mTORC1 does not play a dominant role in lifespan extension under metformin treatment.

References for Supplementary Notes

64 Zhang, C. S. *et al.* Metformin Activates AMPK through the Lysosomal Pathway. *Cell metabolism* **24**, 521-522, doi:10.1016/j.cmet.2016.09.003 (2016).

65 Zhang, Y. L. *et al.* AMP as a low-energy charge signal autonomously initiates assembly of AXIN-AMPK-LKB1 complex for AMPK activation. *Cell Metab.* **18**, 546-555, doi:10.1016/j.cmet.2013.09.005 (2013).

66 Rodgers, T., Leahy, D. & Rowland, M. Physiologically based pharmacokinetic modeling 1: predicting the tissue distribution of moderate-to-strong bases. *J Pharm Sci* **94**, 1259-1276, doi:10.1002/jps.20322 (2005).

67 Moonira, T. *et al.* Metformin lowers glucose 6-phosphate in hepatocytes by activation of glycolysis downstream of glucose phosphorylation. *The Journal of biological chemistry* **295**, 3330-3346, doi:10.1074/jbc.RA120.012533 (2020).
68 Higgins, J. W., Bedwell, D. W. & Zamek-Gliszczynski, M. J. Ablation of both organic cation transporter (OCT)1 and OCT2 alters metformin pharmacokinetics but has no effect on tissue drug exposure and pharmacodynamics. *Drug Metab Dispos* **40**, 1170-1177, doi:10.1124/dmd.112.044875 (2012).
69 Choi, Y. H., Kim, S. G. & Lee, M. G. Dose-independent pharmacokinetics of metformin in rats: Hepatic and gastrointestinal first-pass effects. *J Pharm Sci* **95**, 2543-2552, doi:10.1002/jps.20744 (2006).
70 Chapel, A. *et al.* An extended proteome map of the lysosomal membrane reveals novel potential transporters. *Mol Cell Proteomics* **12**, 1572-1588, doi:10.1074/mcp.M112.021980 (2013).
71 Coen, K. *et al.* Lysosomal calcium homeostasis defects, not proton pump defects, cause endo-lysosomal dysfunction in PSEN-deficient cells. *The Journal of cell biology* **198**, 23-35, doi:10.1083/jcb.201201076 (2012).
72 Lee, J. H. *et al.* Lysosomal proteolysis and autophagy require presenilin 1 and are disrupted by Alzheimer-related PS1 mutations. *Cell* **141**, 1146-1158, doi:10.1016/j.cell.2010.05.008 (2010).
73 Kopan, R. & Ilagan, M. X. Gamma-secretase: proteasome of the membrane? *Nature reviews. Molecular cell biology* **5**, 499-504, doi:10.1038/nrm1406 (2004).
74 De Strooper, B. Aph-1, Pen-2, and Nicastrin with Presenilin generate an active gamma-Secretase complex. *Neuron* **38**, 9-12, doi:10.1016/s0896-6273(03)00205-8 (2003).
75 Zhou, R. *et al.* Recognition of the amyloid precursor protein by human gamma-secretase. *Science* **363**, doi:10.1126/science.aaw0930 (2019).
76 Kopan, R. & Goate, A. Aph-2/Nicastrin: an essential component of gamma-secretase and regulator of Notch signaling and Presenilin localization. *Neuron* **33**, 321-324, doi:10.1016/s0896-6273(02)00585-8 (2002).
77 Dovey, H. F. *et al.* Functional gamma-secretase inhibitors reduce beta-amyloid peptide levels in brain. *Journal of neurochemistry* **76**, 173-181, doi:10.1046/j.1471-4159.2001.00012.x (2001).
78 Luistro, L. *et al.* Preclinical profile of a potent gamma-secretase inhibitor targeting notch signaling with in vivo efficacy and pharmacodynamic properties. *Cancer Res* **69**, 7672-7680, doi:10.1158/0008-5472.CAN-09-1843 (2009).
79 Zhang, Y. W. *et al.* Nicastrin is critical for stability and trafficking but not association of other presenilin/gamma-secretase components. *The Journal of biological chemistry* **280**, 17020-17026, doi:10.1074/jbc.M409467200 (2005).
80 Sannerud, R. *et al.* Restricted Location of PSEN2/gamma-Secretase Determines Substrate Specificity and Generates an Intracellular Abeta Pool. *Cell* **166**, 193-208, doi:10.1016/j.cell.2016.05.020 (2016).
81 Wang, H. *et al.* Presenilins and gamma-secretase inhibitors affect intracellular

trafficking and cell surface localization of the gamma-secretase complex components. *The Journal of biological chemistry* **279**, 40560-40566, doi:10.1074/jbc.M404345200 (2004).
82 De Strooper, B. *et al.* A presenilin-1-dependent gamma-secretase-like protease mediates release of Notch intracellular domain. *Nature* **398**, 518-522, doi:10.1038/19083 (1999).
83 Son, S. M. *et al.* Metformin Facilitates Amyloid-beta Generation by beta- and gamma-Secretases via Autophagy Activation. *J Alzheimers Dis* **51**, 1197-1208, doi:10.3233/JAD-151200 (2016).
84 Ou, Z. *et al.* Metformin treatment prevents amyloid plaque deposition and memory impairment in APP/PS1 mice. *Brain Behav Immun* **69**, 351-363, doi:10.1016/j.bbi.2017.12.009 (2018).
85 Wakabayashi, T. *et al.* Analysis of the gamma-secretase interactome and validation of its association with tetraspanin-enriched microdomains. *Nature cell biology* **11**, 1340-1346, doi:10.1038/ncb1978 (2009).
86 Nylandsted, J. *et al.* ErbB2-associated changes in the lysosomal proteome. *Proteomics* **11**, 2830-2838, doi:10.1002/pmic.201000734 (2011).
87 Meckler, X. & Checler, F. Visualization of specific gamma-secretase complexes using bimolecular fluorescence complementation. *J Alzheimers Dis* **40**, 161-176, doi:10.3233/JAD-131268 (2014).
88 Oakhill, J. S. *et al.* beta-Subunit myristoylation is the gatekeeper for initiating metabolic stress sensing by AMP-activated protein kinase (AMPK). *Proceedings of the National Academy of Sciences of the United States of America* **107**, 19237-19241, doi:10.1073/pnas.1009705107 (2010).
89 Repiscak, P., Erhardt, S., Rena, G. & Paterson, M. J. Biomolecular mode of action of metformin in relation to its copper binding properties. *Biochemistry* **53**, 787-795, doi:10.1021/bi401444n (2014).
90 Muller, S. *et al.* Metformin reveals a mitochondrial copper addiction of mesenchymal cancer cells. *PLoS One* **13**, e0206764, doi:10.1371/journal.pone.0206764 (2018).
91 Stynen, B. *et al.* Changes of Cell Biochemical States Are Revealed in Protein Homomeric Complex Dynamics. *Cell* **175**, 1418-1429 e1419, doi:10.1016/j.cell.2018.09.050 (2018).
92 Wang, W. *et al.* A voltage-dependent K(+) channel in the lysosome is required for refilling lysosomal Ca(2+) stores. *The Journal of cell biology* **216**, 1715-1730, doi:10.1083/jcb.201612123 (2017).
93 Li, Y., Liu, L., Wang, B., Wang, J. & Chen, D. Metformin in non-alcoholic fatty liver disease: A systematic review and meta-analysis. *Biomed Rep* **1**, 57-64, doi:10.3892/br.2012.18 (2013).
94 Blazina, I. & Selph, S. Diabetes drugs for nonalcoholic fatty liver disease: a systematic review. *Syst Rev* **8**, 295, doi:10.1186/s13643-019-1200-8 (2019).
95 Bagherniya, M., Butler, A. E., Barreto, G. E. & Sahebkar, A. The effect of fasting or calorie restriction on autophagy induction: A review of the literature. *Ageing research reviews* **47**, 183-197, doi:10.1016/j.arr.2018.08.004 (2018).

96 Schulz, T. J. *et al.* Glucose restriction extends Caenorhabditis elegans life span by inducing mitochondrial respiration and increasing oxidative stress. *Cell metabolism* **6**, 280-293, doi:10.1016/j.cmet.2007.08.011 (2007).
97 Schmeisser, S. *et al.* Neuronal ROS signaling rather than AMPK/sirtuin-mediated energy sensing links dietary restriction to lifespan extension. *Mol Metab* **2**, 92-102, doi:10.1016/j.molmet.2013.02.002 (2013).
98 Anedda, A., Rial, E. & Gonzalez-Barroso, M. M. Metformin induces oxidative stress in white adipocytes and raises uncoupling protein 2 levels. *J Endocrinol* **199**, 33-40, doi:10.1677/JOE-08-0278 (2008).
99 De Haes, W. *et al.* Metformin promotes lifespan through mitohormesis via the peroxiredoxin PRDX-2. *Proceedings of the National Academy of Sciences of the United States of America* **111**, E2501-2509, doi:10.1073/pnas.1321776111 (2014).
100 Auciello, F. R., Ross, F. A., Ikematsu, N. & Hardie, D. G. Oxidative stress activates AMPK in cultured cells primarily by increasing cellular AMP and/or ADP. *FEBS letters* **588**, 3361-3366, doi:10.1016/j.febslet.2014.07.025 (2014).
101 Hinchy, E. C. *et al.* Mitochondria-derived ROS activate AMP-activated protein kinase (AMPK) indirectly. *The Journal of biological chemistry* **293**, 17208-17217, doi:10.1074/jbc.RA118.002579 (2018).
102 Zoncu, R. *et al.* mTORC1 senses lysosomal amino acids through an inside-out mechanism that requires the vacuolar H(+)-ATPase. *Science* **334**, 678-683, doi:10.1126/science.1207056 (2011).
103 Jansen, E. J., Hafmans, T. G. & Martens, G. J. V-ATPase-mediated granular acidification is regulated by the V-ATPase accessory subunit Ac45 in POMC-producing cells. *Mol Biol Cell* **21**, 3330-3339, doi:10.1091/mbc.E10-04-0274 (2010).
104 Jansen, E. J. *et al.* ATP6AP1 deficiency causes an immunodeficiency with hepatopathy, cognitive impairment and abnormal protein glycosylation. *Nat Commun* **7**, 11600, doi:10.1038/ncomms11600 (2016).
105 Pareja, F. *et al.* Loss-of-function mutations in ATP6AP1 and ATP6AP2 in granular cell tumors. *Nat Commun* **9**, 3533, doi:10.1038/s41467-018-05886-y (2018).
106 Lu, M., Holliday, L. S., Zhang, L., Dunn, W. A., Jr. & Gluck, S. L. Interaction between aldolase and vacuolar H+-ATPase: evidence for direct coupling of glycolysis to the ATP-hydrolyzing proton pump. *The Journal of biological chemistry* **276**, 30407-30413, doi:10.1074/jbc.M008768200 (2001).
107 Lu, M., Sautin, Y. Y., Holliday, L. S. & Gluck, S. L. The glycolytic enzyme aldolase mediates assembly, expression, and activity of vacuolar H+-ATPase. *The Journal of biological chemistry* **279**, 8732-8739, doi:10.1074/jbc.M303871200 (2004).
108 Lu, M., Ammar, D., Ives, H., Albrecht, F. & Gluck, S. L. Physical interaction between aldolase and vacuolar H+-ATPase is essential for the assembly and activity of the proton pump. *The Journal of biological chemistry* **282**, 24495-24503, doi:10.1074/jbc.M702598200 (2007).

109 Waise, T. M. Z. *et al.* Inhibition of upper small intestinal mTOR lowers plasma glucose levels by inhibiting glucose production. *Nat Commun* **10**, 714, doi:10.1038/s41467-019-08582-7 (2019).

110 Fang, Y. *et al.* Duration of rapamycin treatment has differential effects on metabolism in mice. *Cell metabolism* **17**, 456-462, doi:10.1016/j.cmet.2013.02.008 (2013).

111 Umemura, A. *et al.* Liver damage, inflammation, and enhanced tumorigenesis after persistent mTORC1 inhibition. *Cell metabolism* **20**, 133-144, doi:10.1016/j.cmet.2014.05.001 (2014).

112 Chang, G. R. *et al.* Long-term administration of rapamycin reduces adiposity, but impairs glucose tolerance in high-fat diet-fed KK/HlJ mice. *Basic Clin Pharmacol Toxicol* **105**, 188-198, doi:10.1111/j.1742-7843.2009.00427.x (2009).

113 Lamming, D. W. *et al.* Rapamycin-induced insulin resistance is mediated by mTORC2 loss and uncoupled from longevity. *Science* **335**, 1638-1643, doi:10.1126/science.1215135 (2012).

114 Yuan, M., Pino, E., Wu, L., Kacergis, M. & Soukas, A. A. Identification of Akt-independent regulation of hepatic lipogenesis by mammalian target of rapamycin (mTOR) complex 2. *The Journal of biological chemistry* **287**, 29579-29588, doi:10.1074/jbc.M112.386854 (2012).

115 Lin, Y. H. *et al.* Diacylglycerol lipase regulates lifespan and oxidative stress response by inversely modulating TOR signaling in Drosophila and C. elegans. *Aging cell* **13**, 755-764, doi:10.1111/acel.12232 (2014).

116 Chen, J. *et al.* Metformin extends C. elegans lifespan through lysosomal pathway. *Elife* **6**, doi:10.7554/eLife.31268 (2017).

Supplementary Fig. 1 | Uncropped gels for Fig. 1 to 4 and Extended Data Fig.1 to 14. After electrophoretic transfer of proteins, the PVDF membranes were cut into strips containing sets of samples, and were then subjected to immunoblotting. Show here are films that had been exposed and developed to the membrane strips. The Pierce™ Prestained Protein MW Marker (Cat. 26612, ThermoFisher Scientific) was used as the protein markers.

Fig. 1b

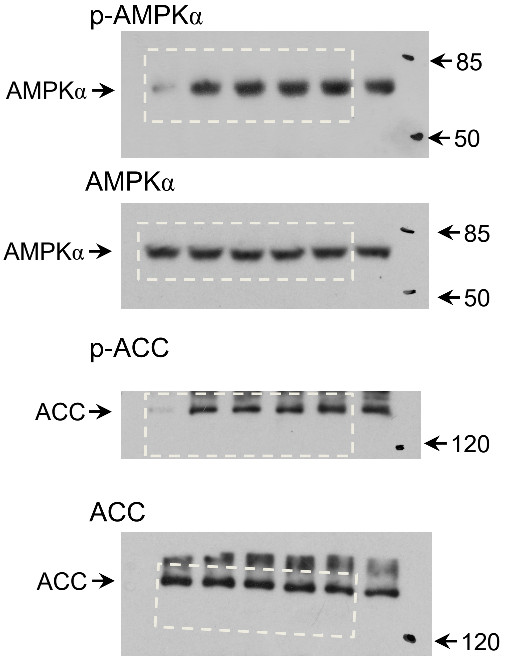

Fig. 2b

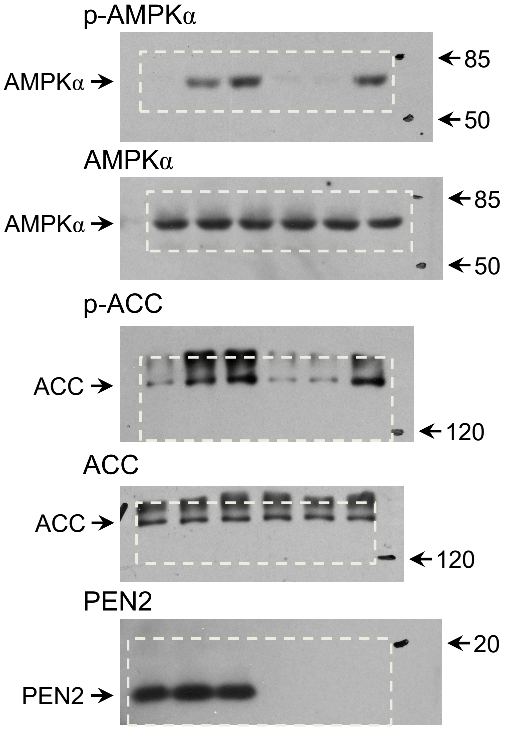

Fig. 2c

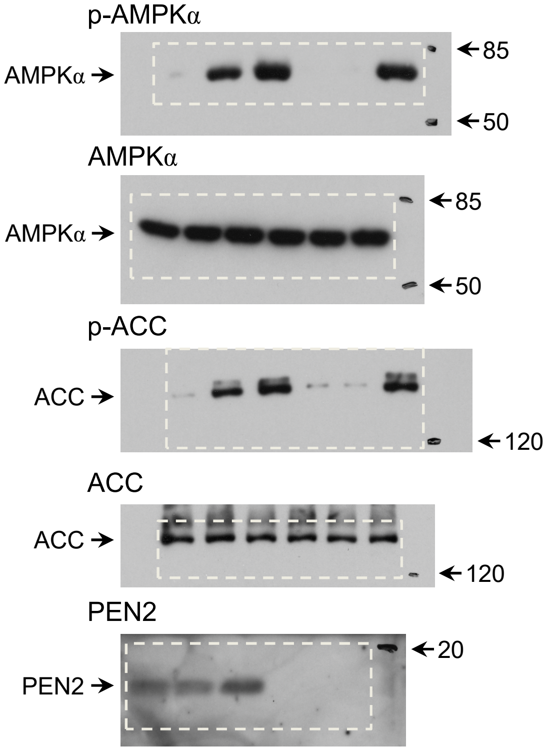

Fig. 2g

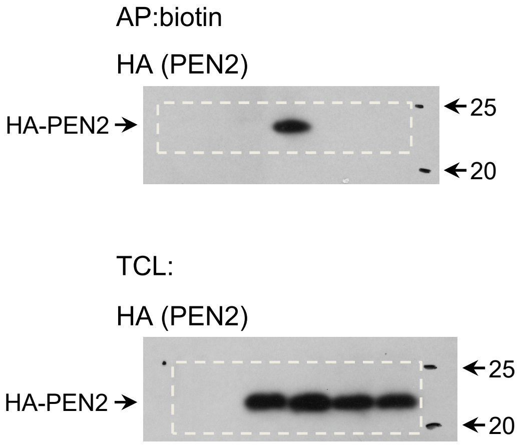

Fig. 2h

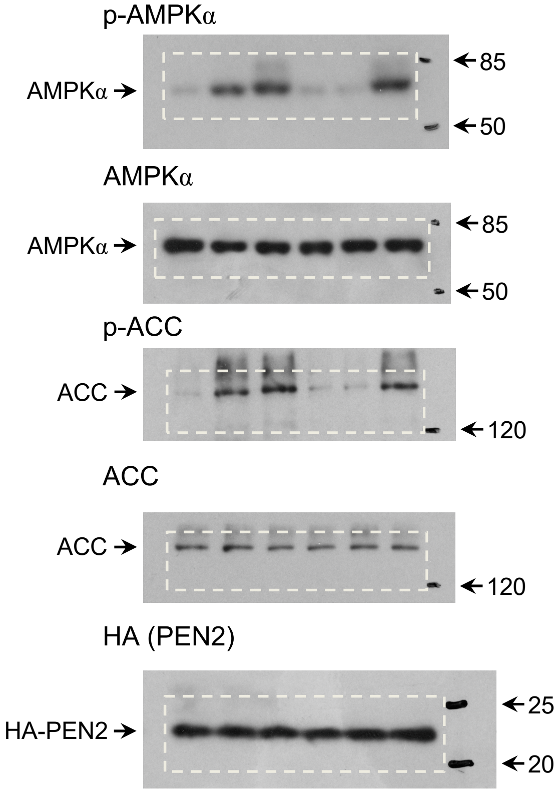

Fig. 3a

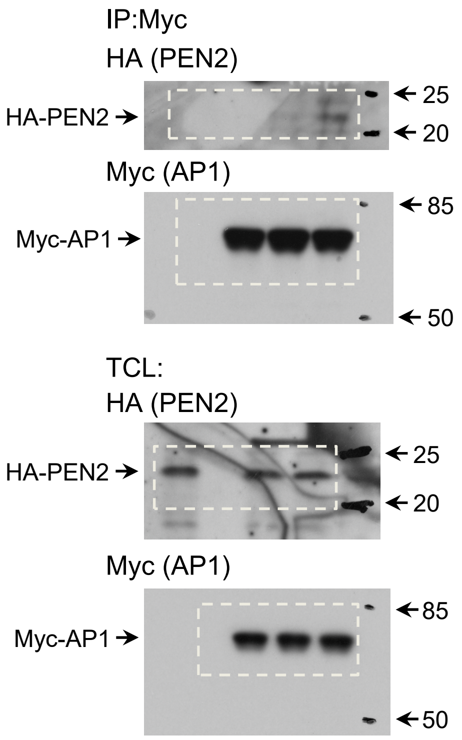

Fig. 3b

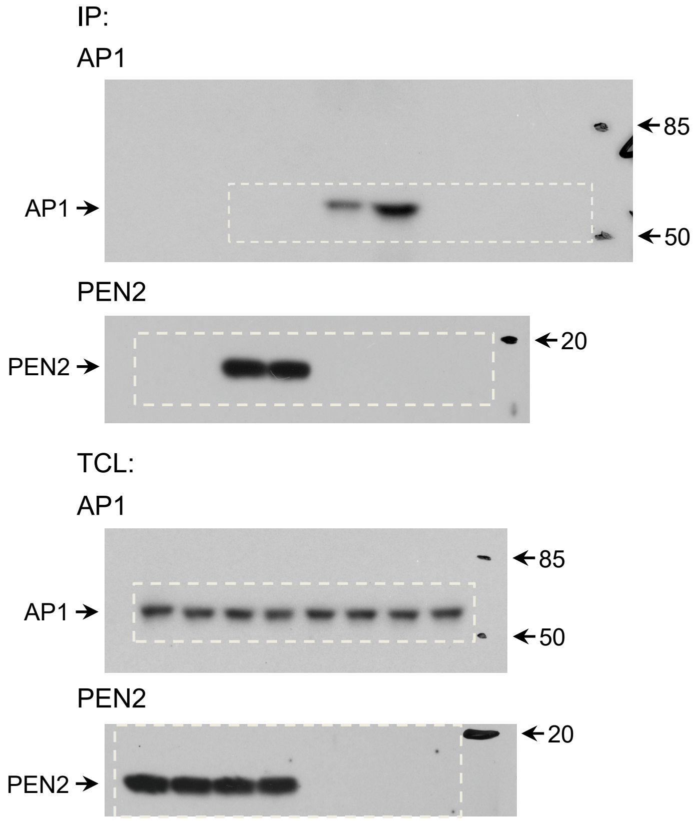

Fig. 3c

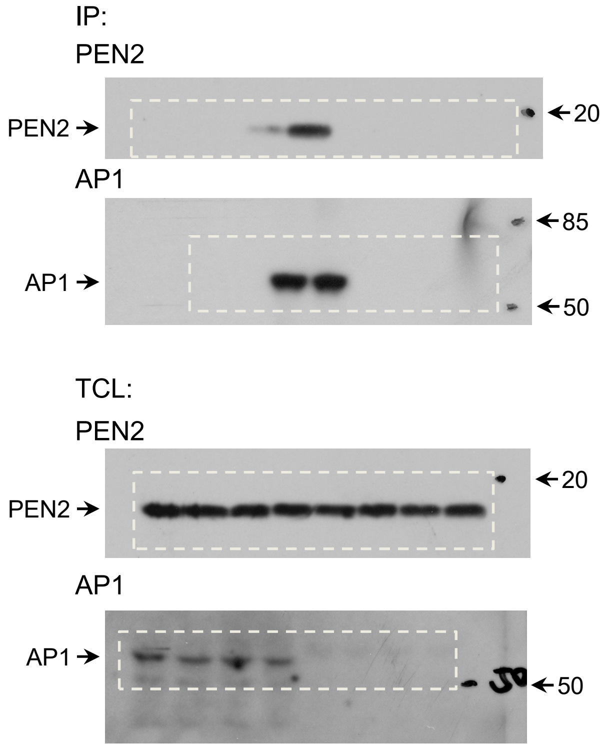

Fig. 3d

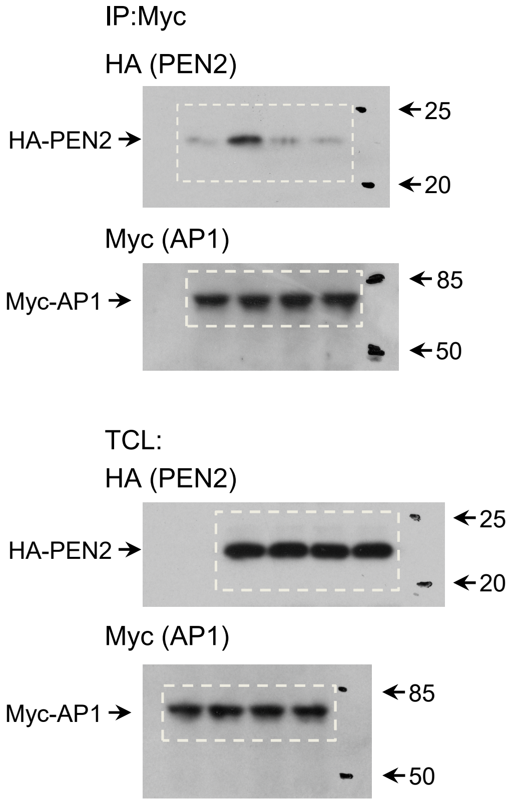

Fig. 3e

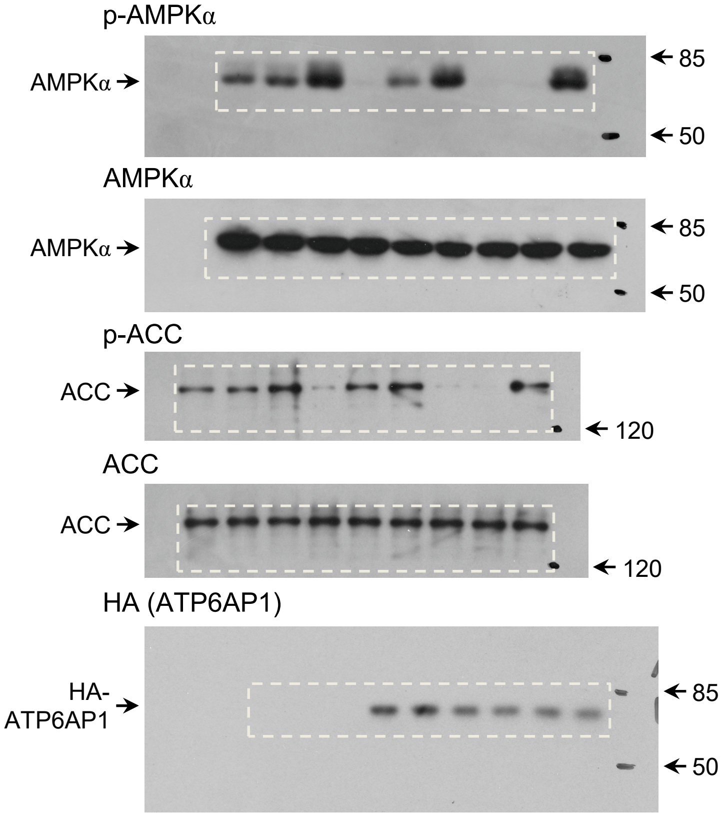

Fig. 3f

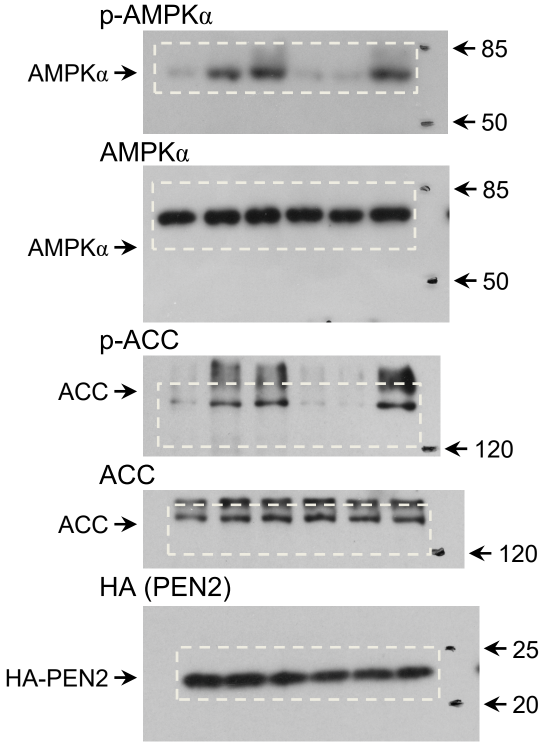

Fig. 3h

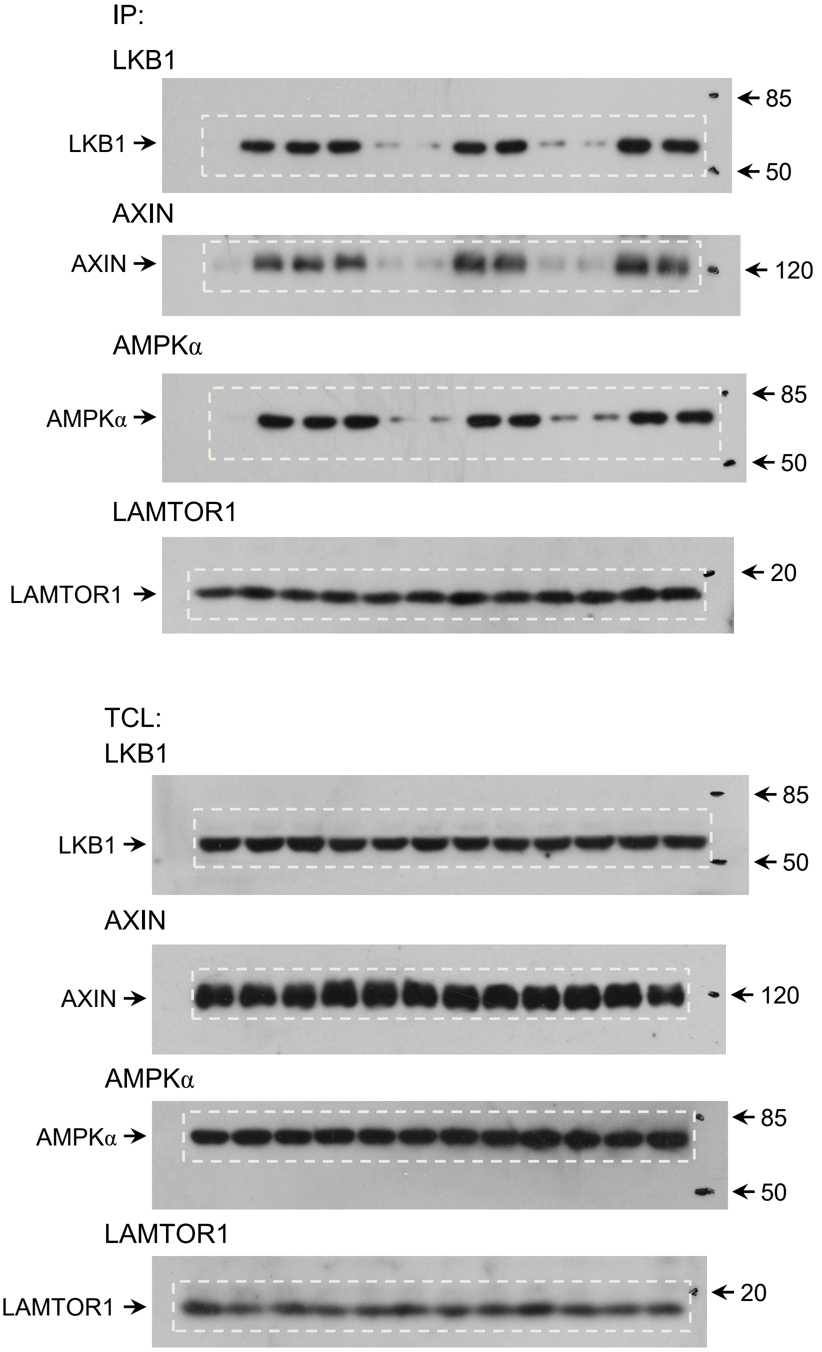
IP:
LKB1
LKB1
85
50
AXIN
AXIN
120
AMPKα
AMPKα
85
50
LAMTOR1
LAMTOR1
20
TCL:
LKB1
LKB1
85
50
AXIN
AXIN
120
AMPKα
AMPKα
85
50
LAMTOR1
LAMTOR1
20

Fig. 3i

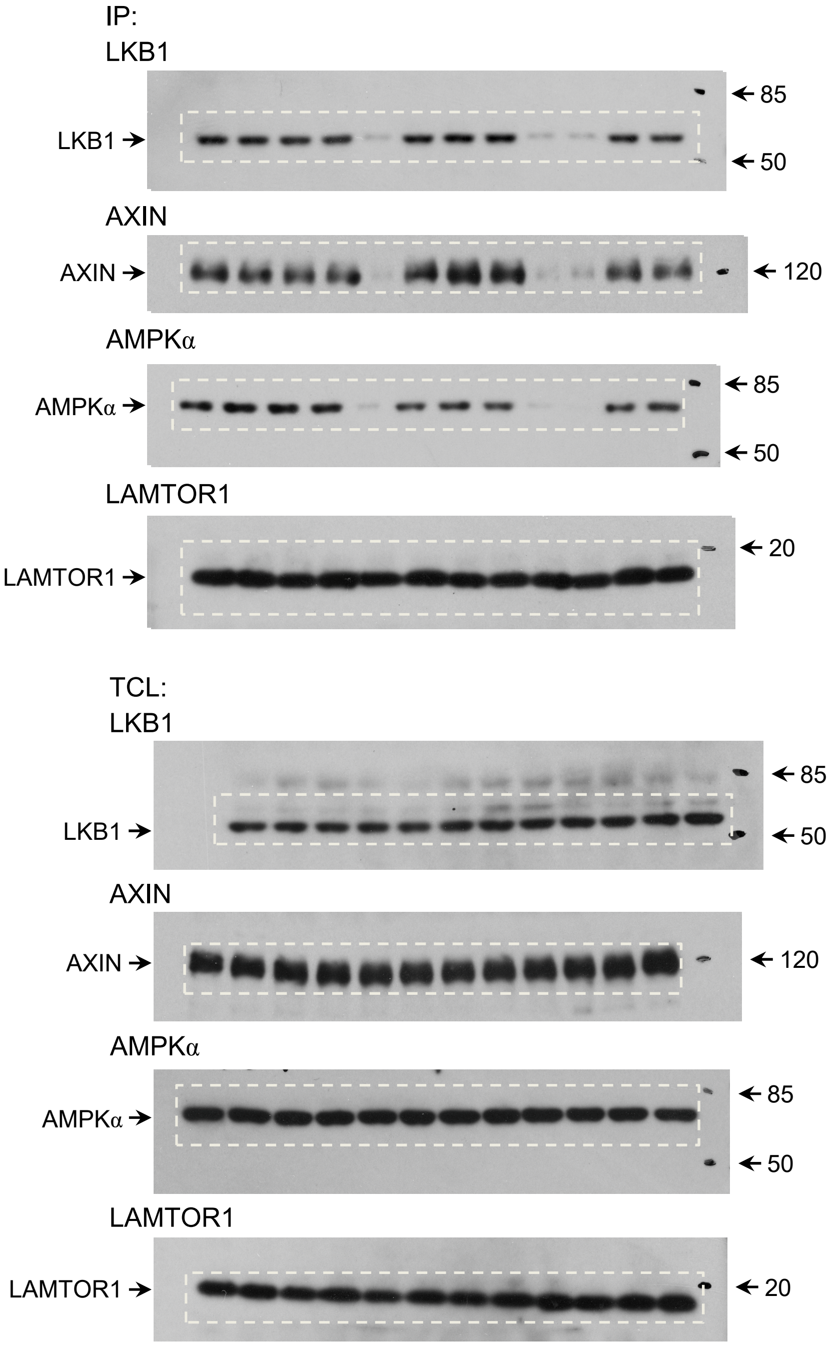
IP:
LKB1
LKB1
85
50
AXIN
AXIN
120
AMPKα
AMPKα
85
50
LAMTOR1
LAMTOR1
20
TCL:
LKB1
LKB1
85
50
AXIN
AXIN
120
AMPKα
AMPKα
85
50
LAMTOR1
LAMTOR1
20

Fig. 4i

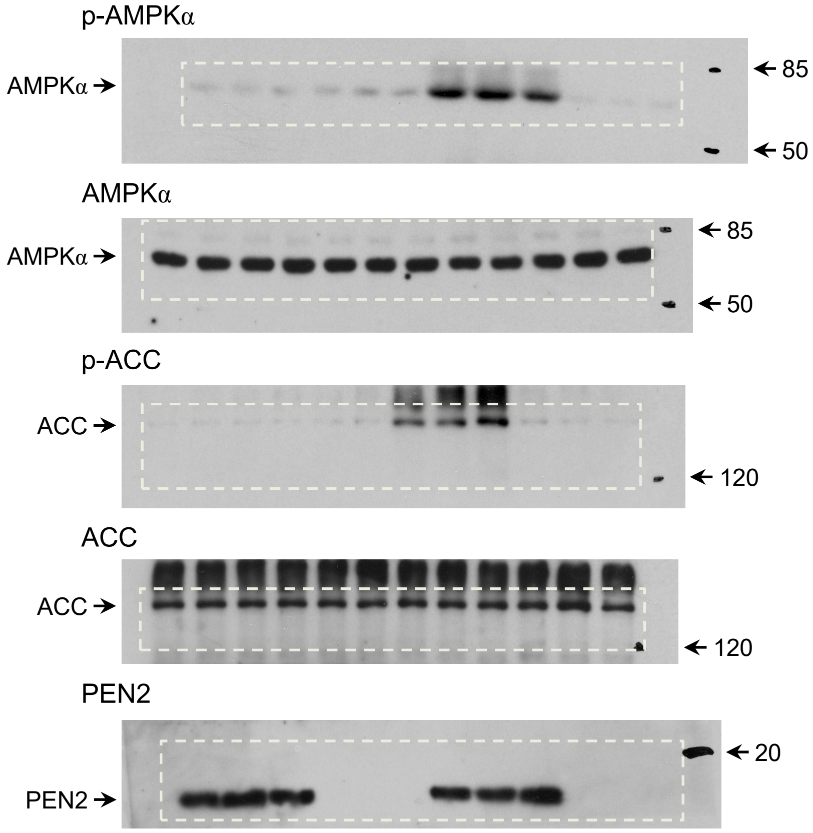
p-AMPKα
AMPKα
85
50
AMPKα
AMPKα
85
50
p-ACC
ACC
120
ACC
ACC
120
PEN2
PEN2
20

Fig. 4j

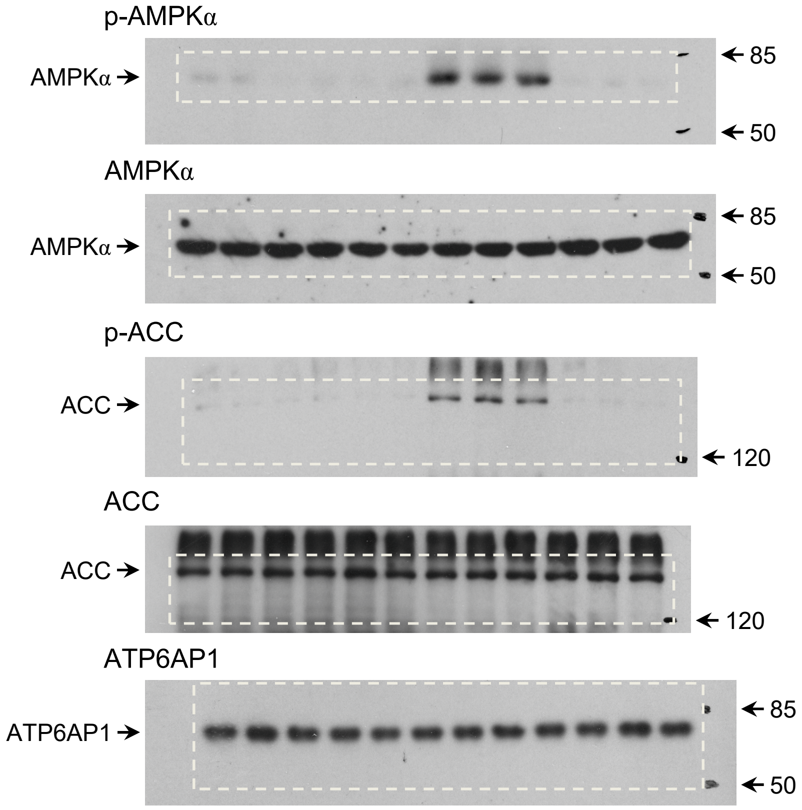
p-AMPKα
AMPKα
85
50
AMPKα
AMPKα
85
50
p-ACC
ACC
120
ACC
ACC
120
ATP6AP1
ATP6AP1
85
50

Fig. S1b

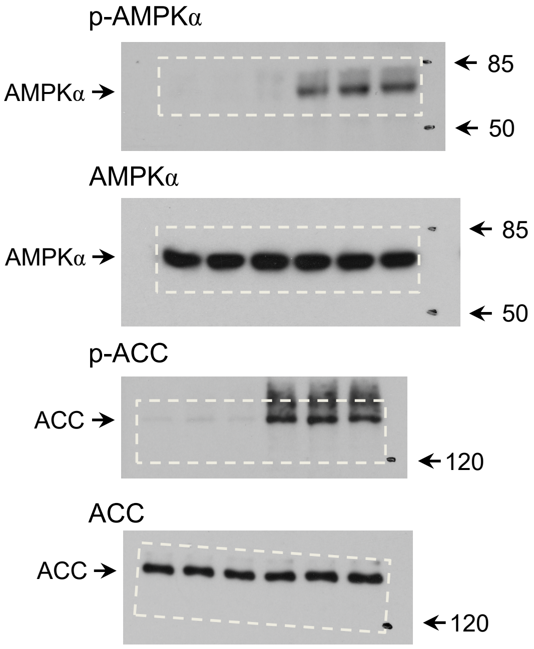

Fig.S1c

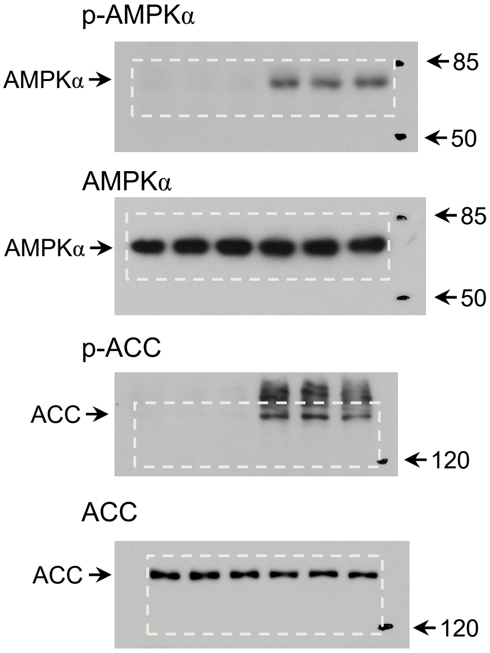

Fig. S1j left

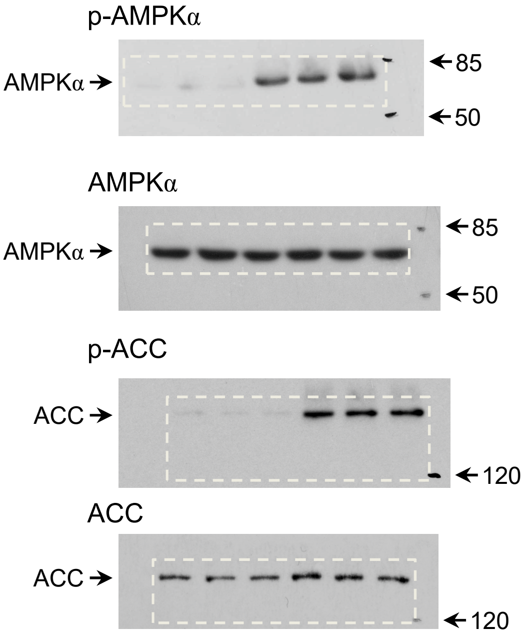

Fig. S1j right

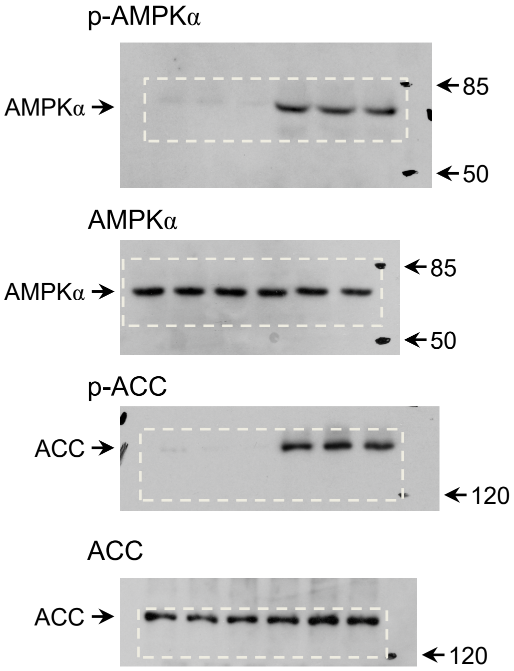

Fig. S1m

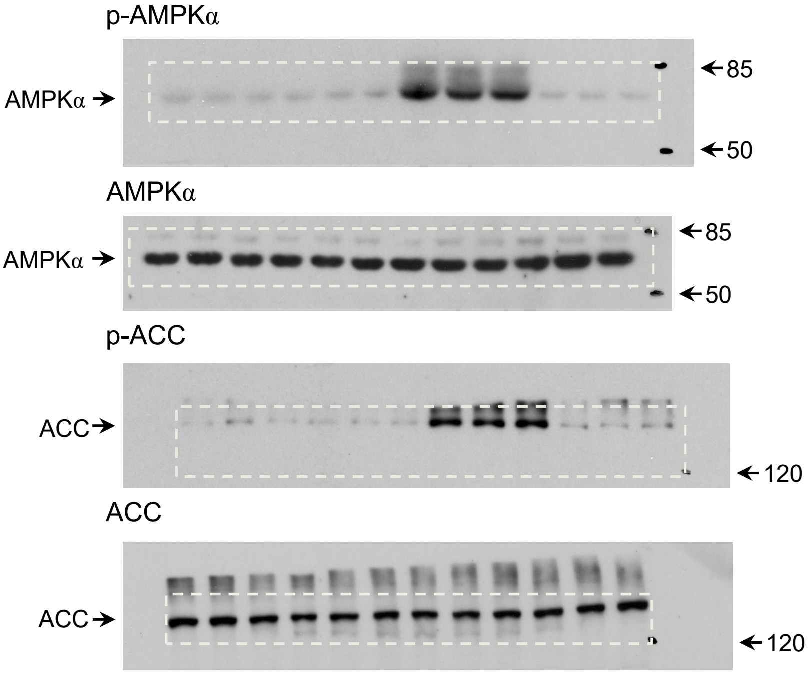

Fig. S1n

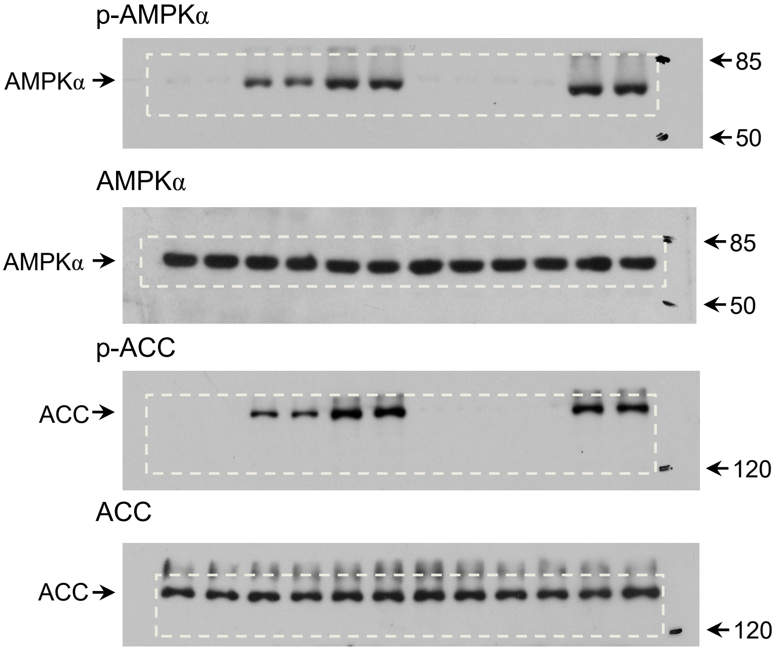

Fig. S1o

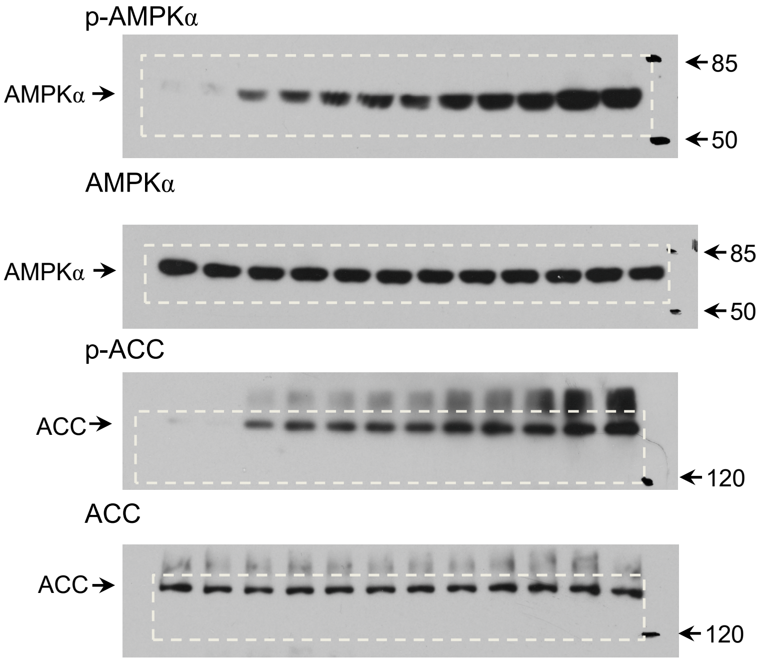

Fig. S1p

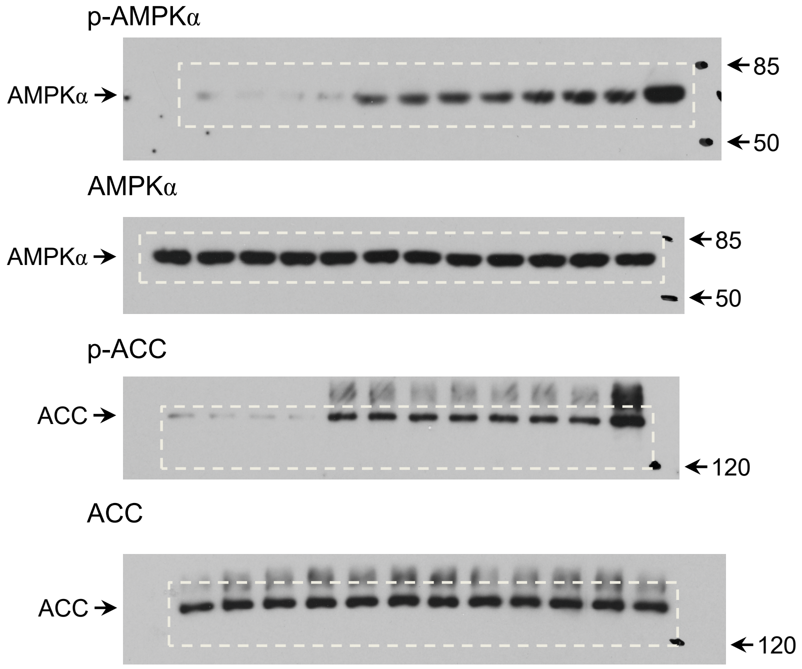

Fig. S1v

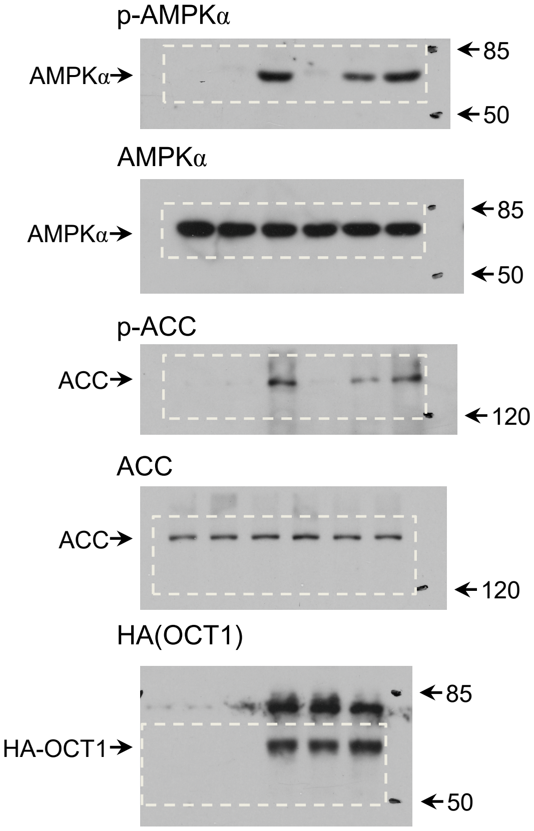

Fig. S1w

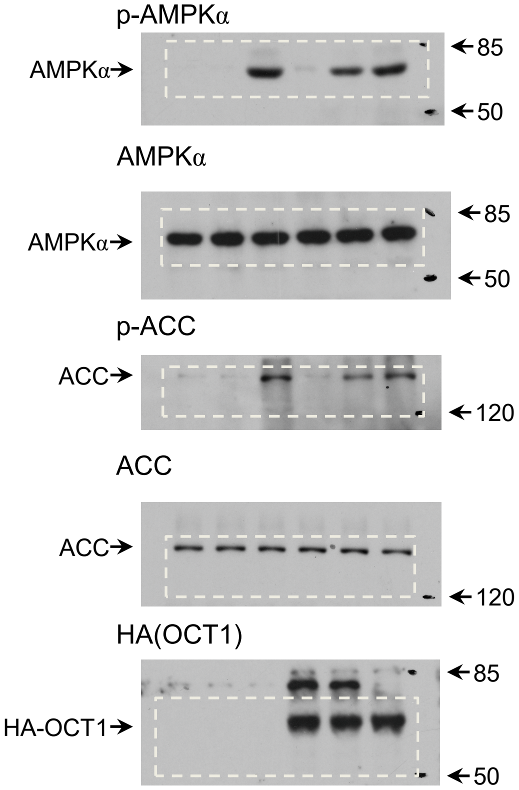

Fig. S2d

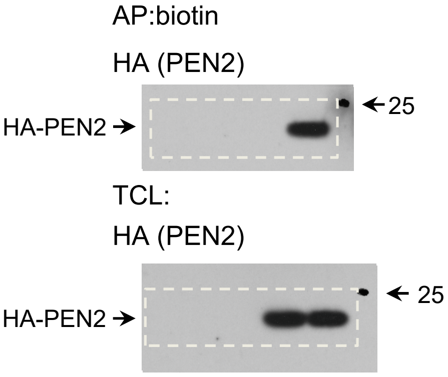

Fig. S3b

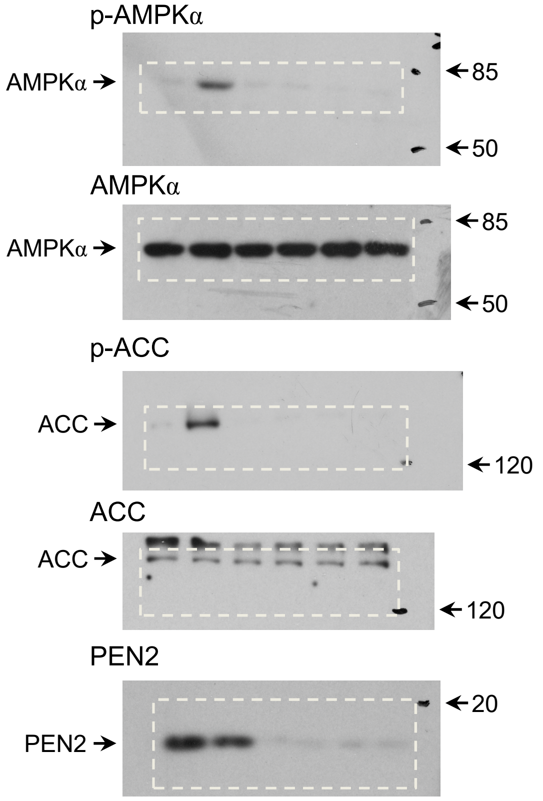

Fig. S3d

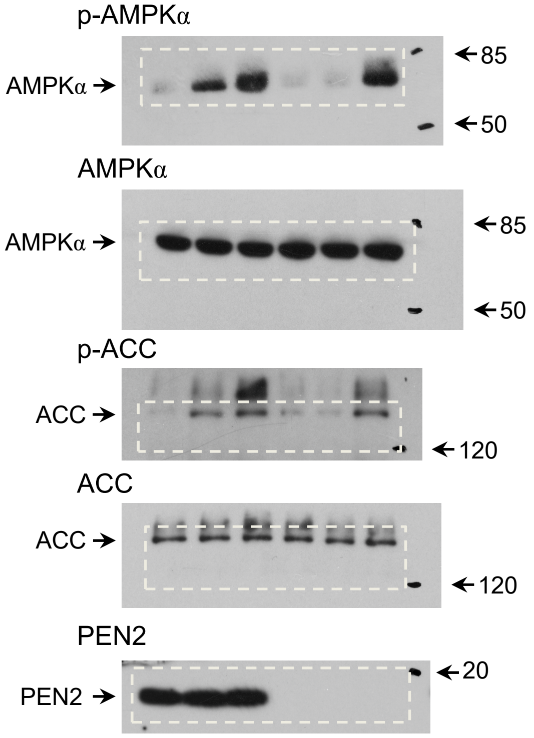

Fig. S3g

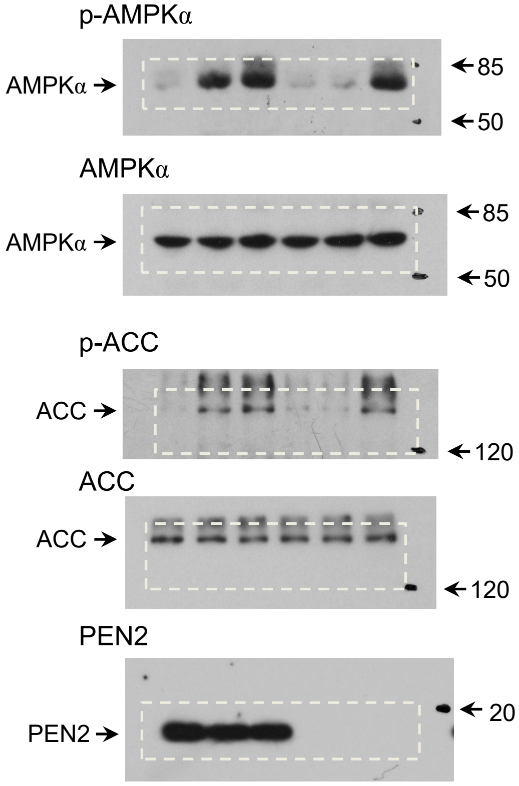

Fig. S3h

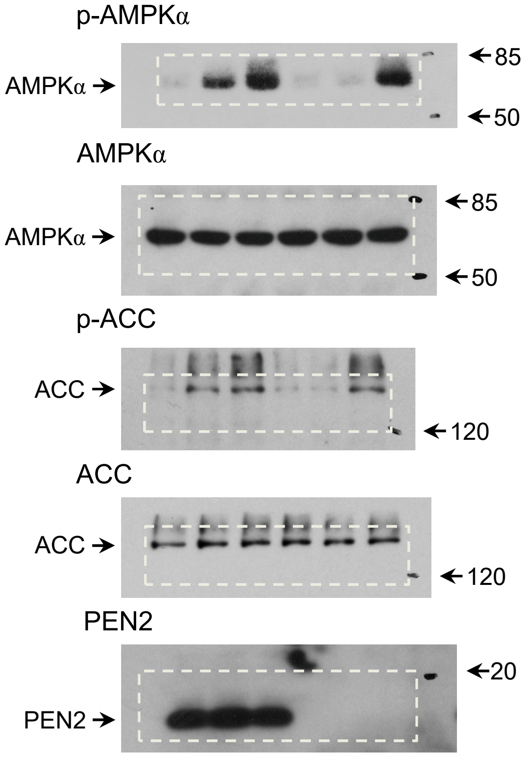

Fig. S3k left

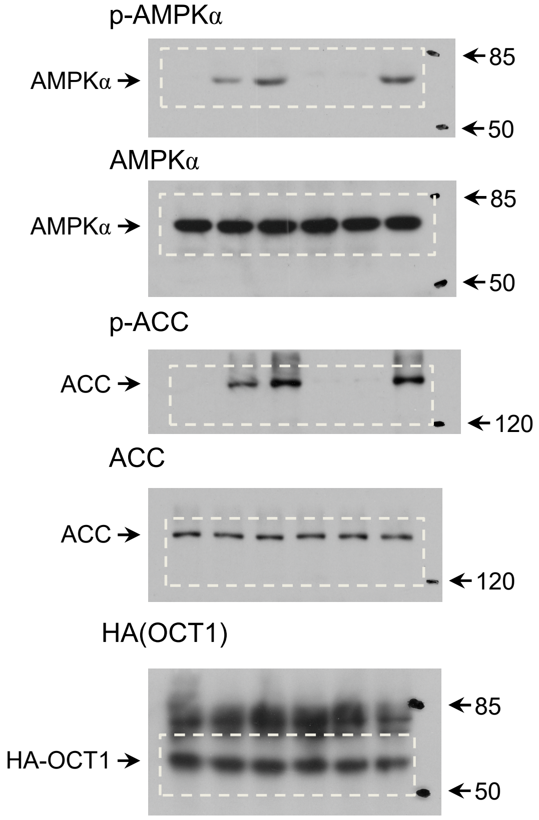

Fig. S3k right

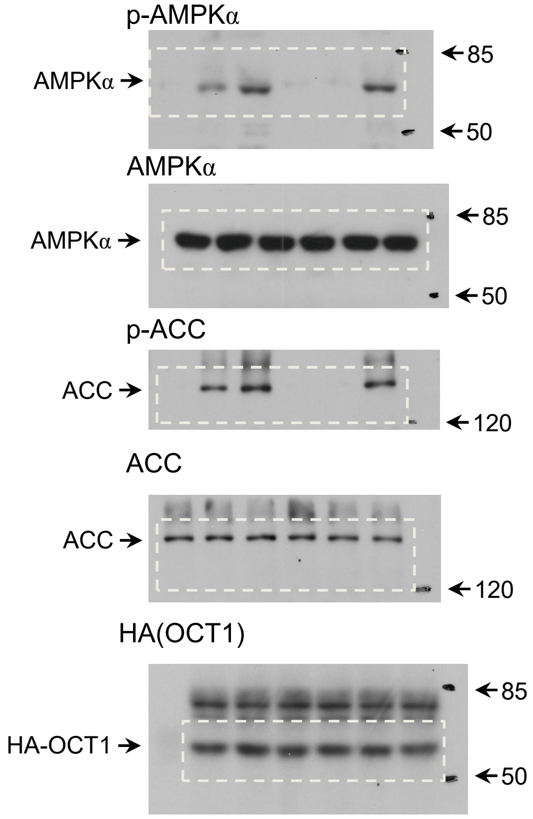

Fig. S3l left

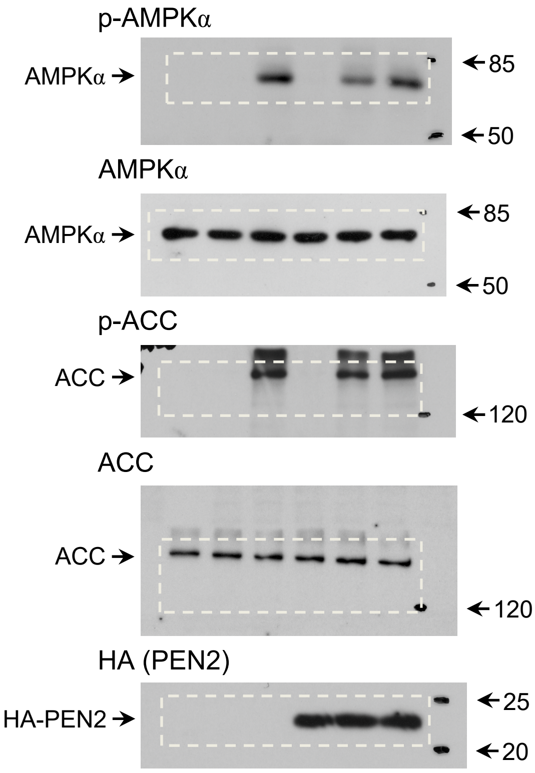

Fig. S3l right

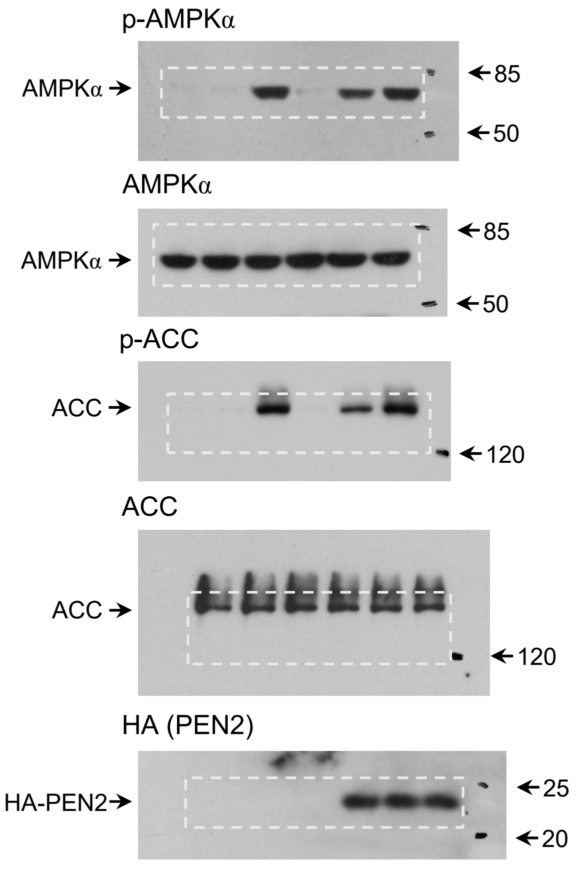

Fig. S3m left

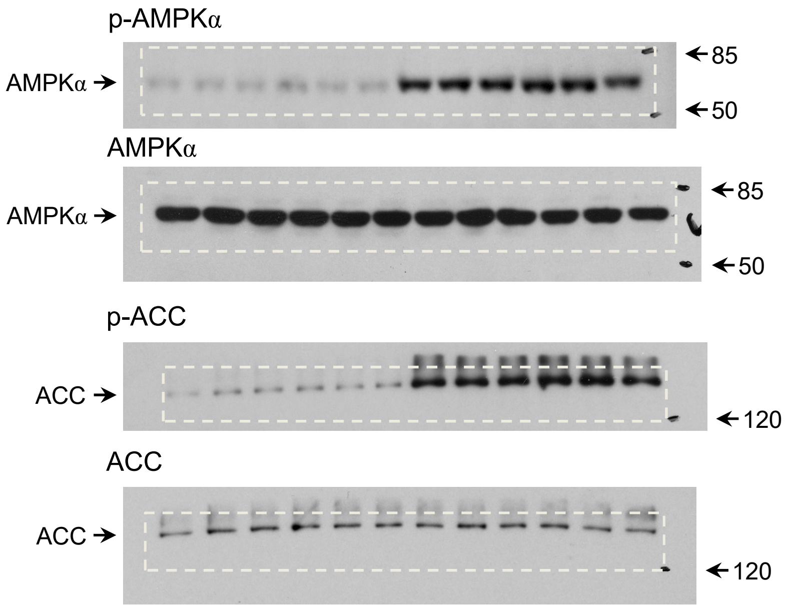

Fig. S3m right

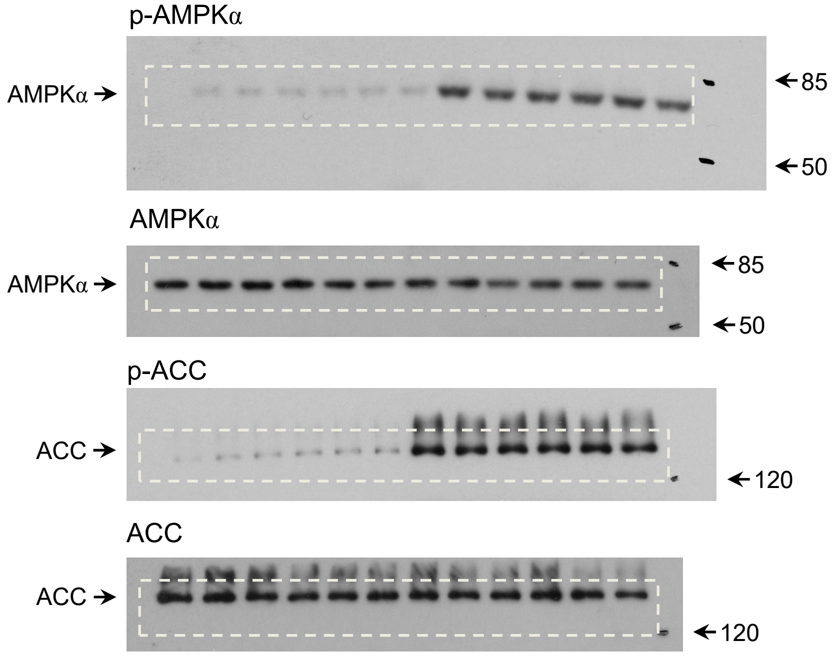

Fig. S3n

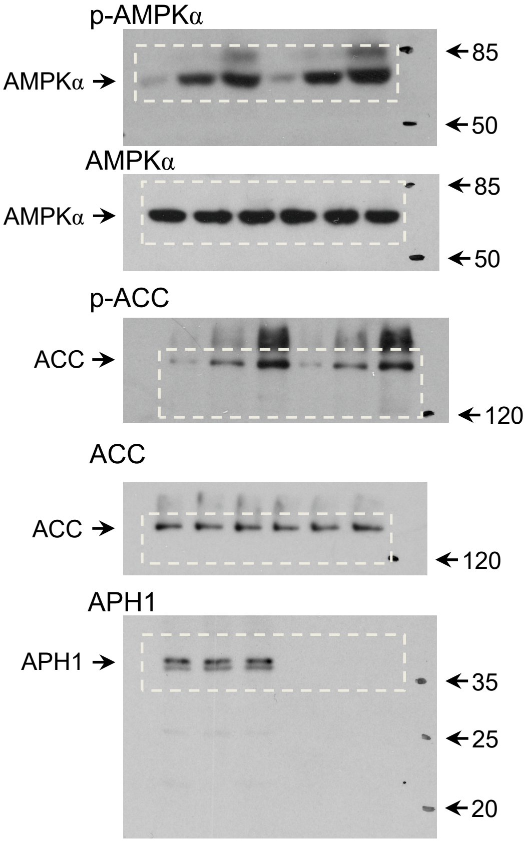

Fig. S3p

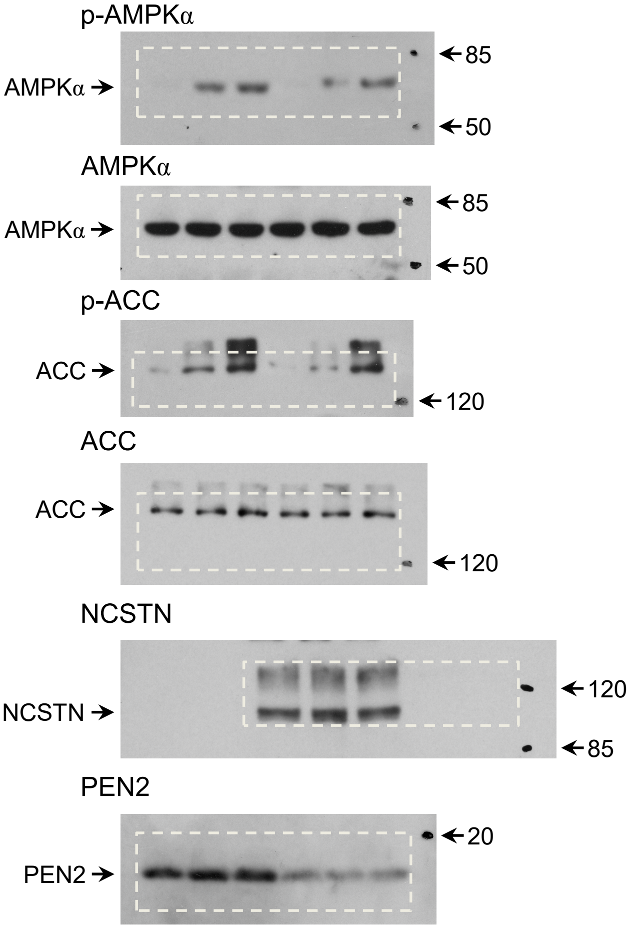

Fig. S3q

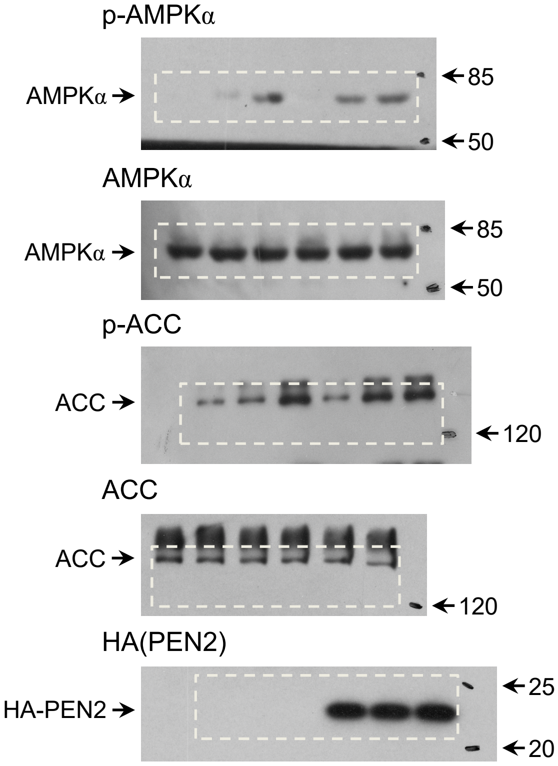

Fig. S3r

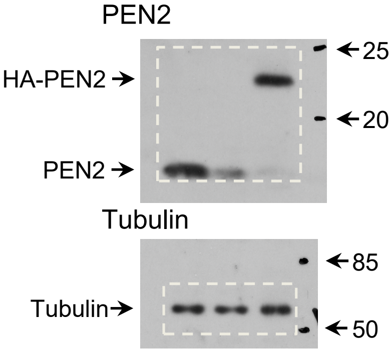

Fig. S4a

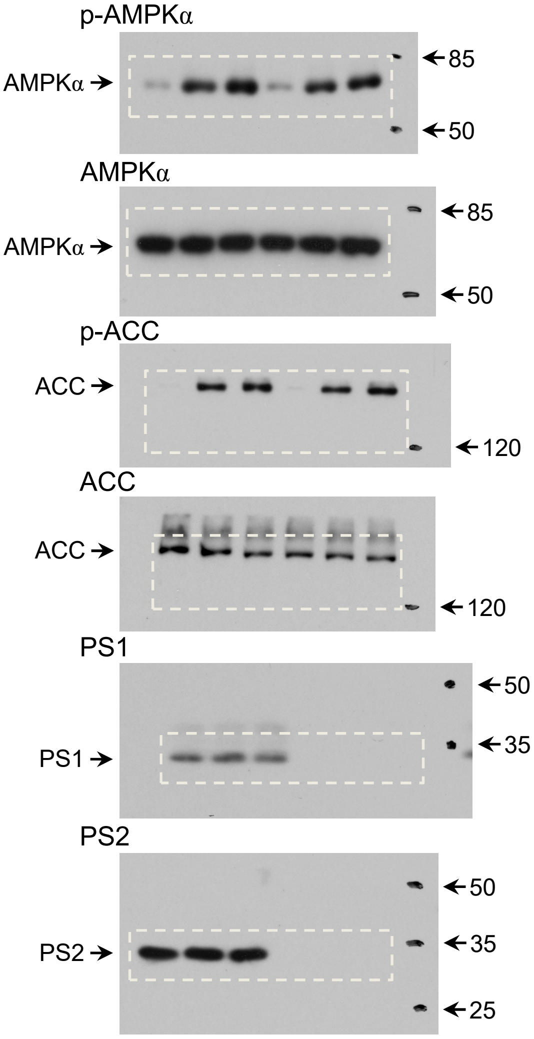

Fig. S4b left

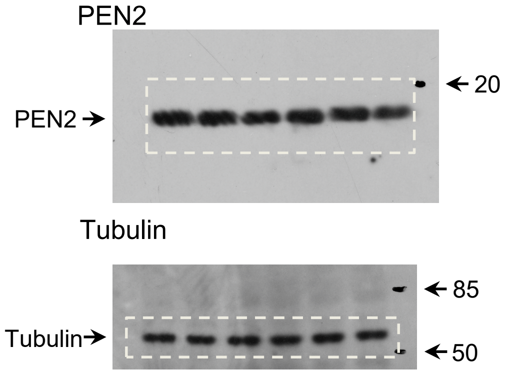

Fig. S4b right

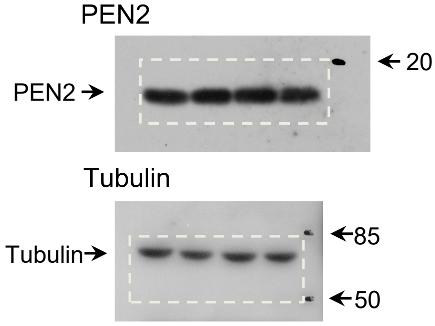

Fig. S4e

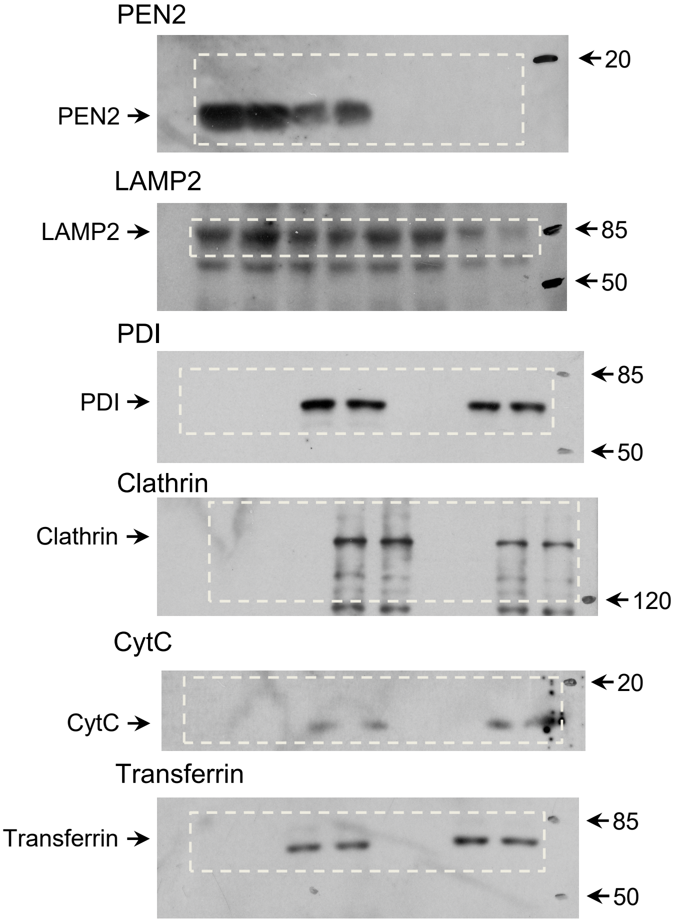

Fig. S5c

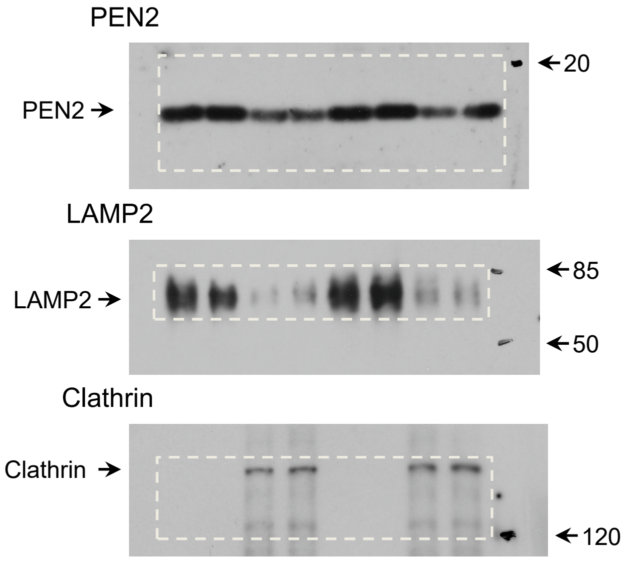

Fig. S5d

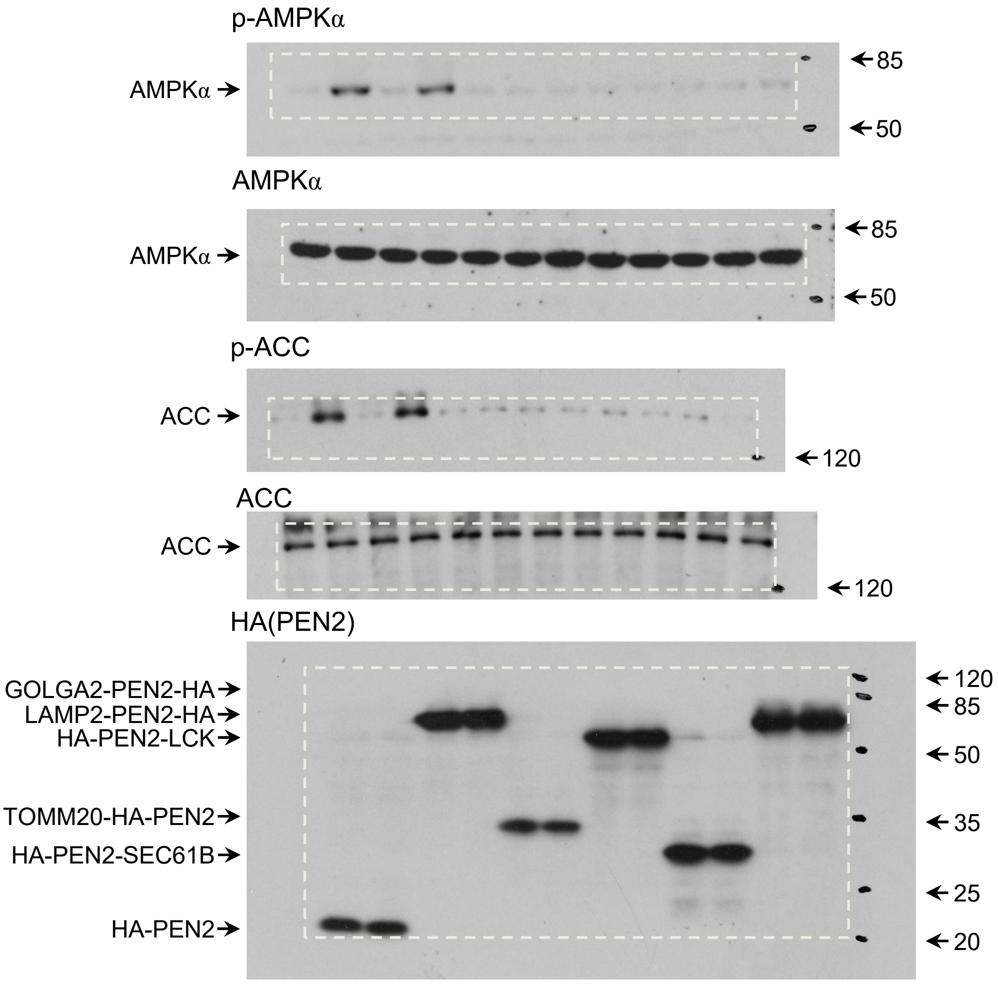

Fig. S5f

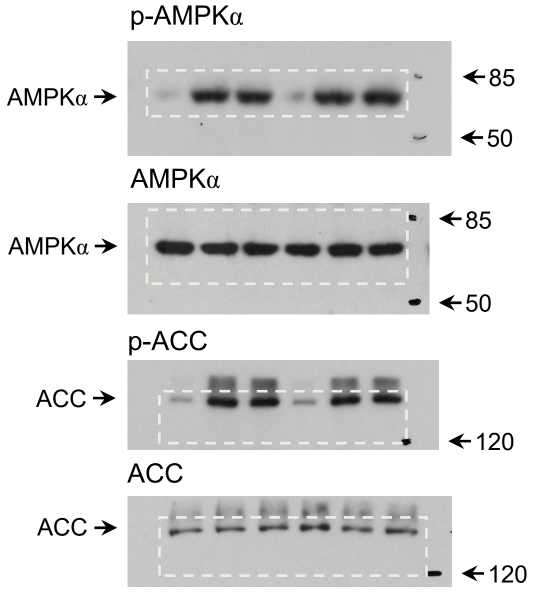

Fig. S5g

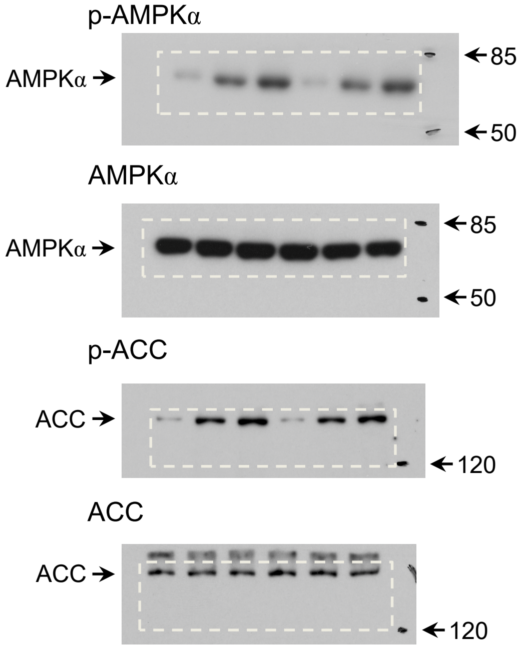

Fig. S5h

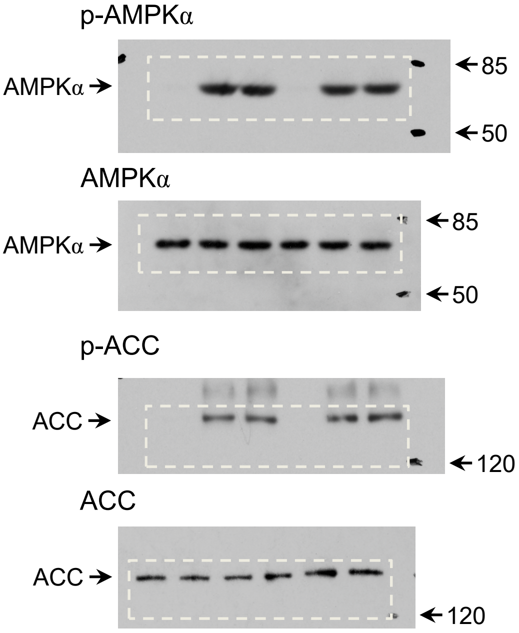

Fig. S6c

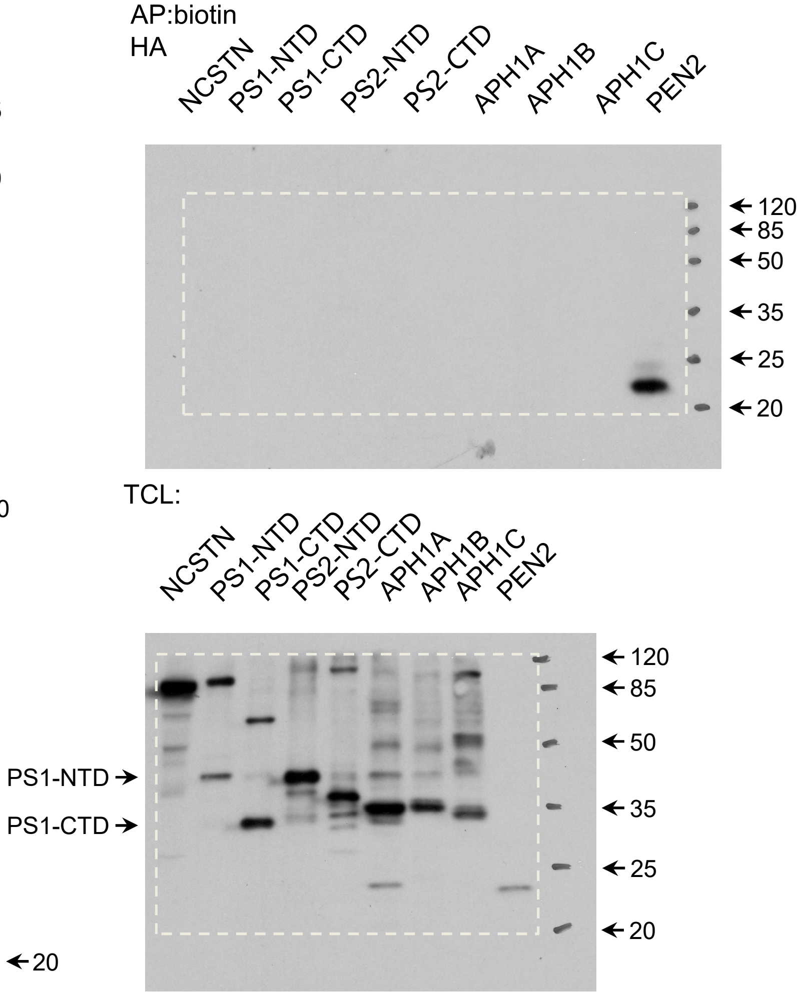

Fig. S6g upper

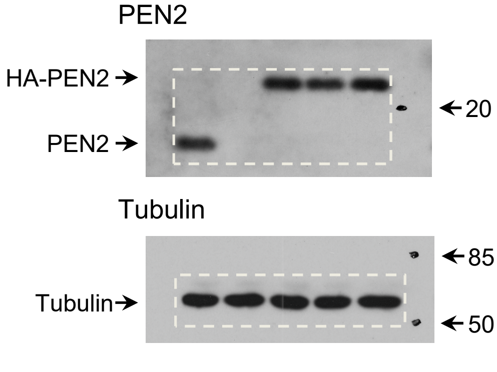

Fig. S6k

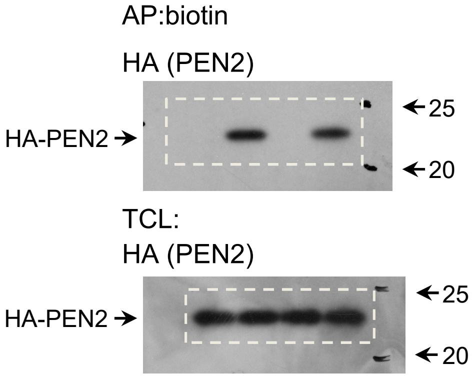

Fig. S6g lower

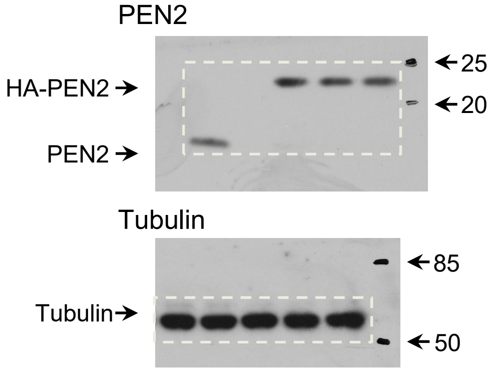

Fig. S6l

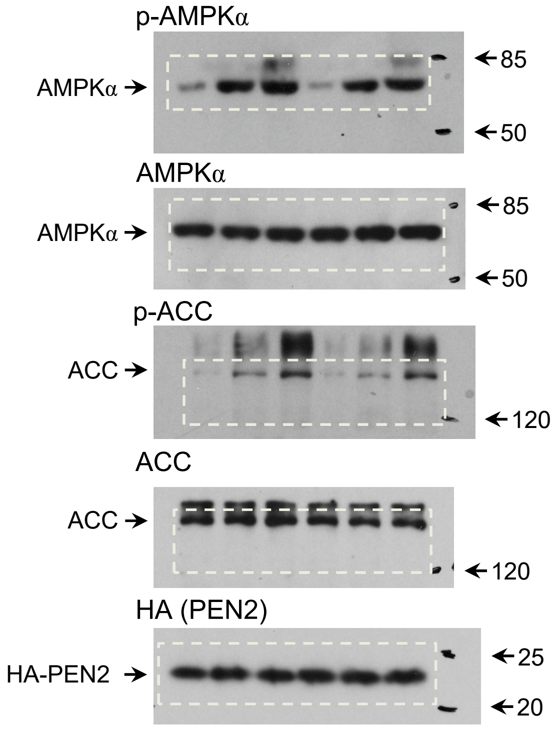

Fig. S6m

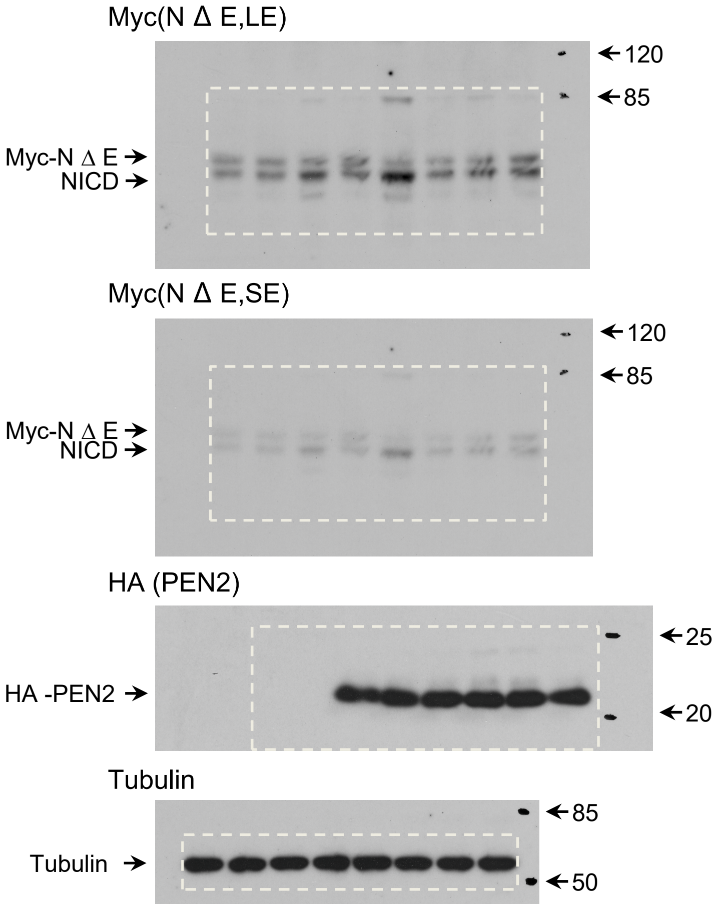

Fig. S6n

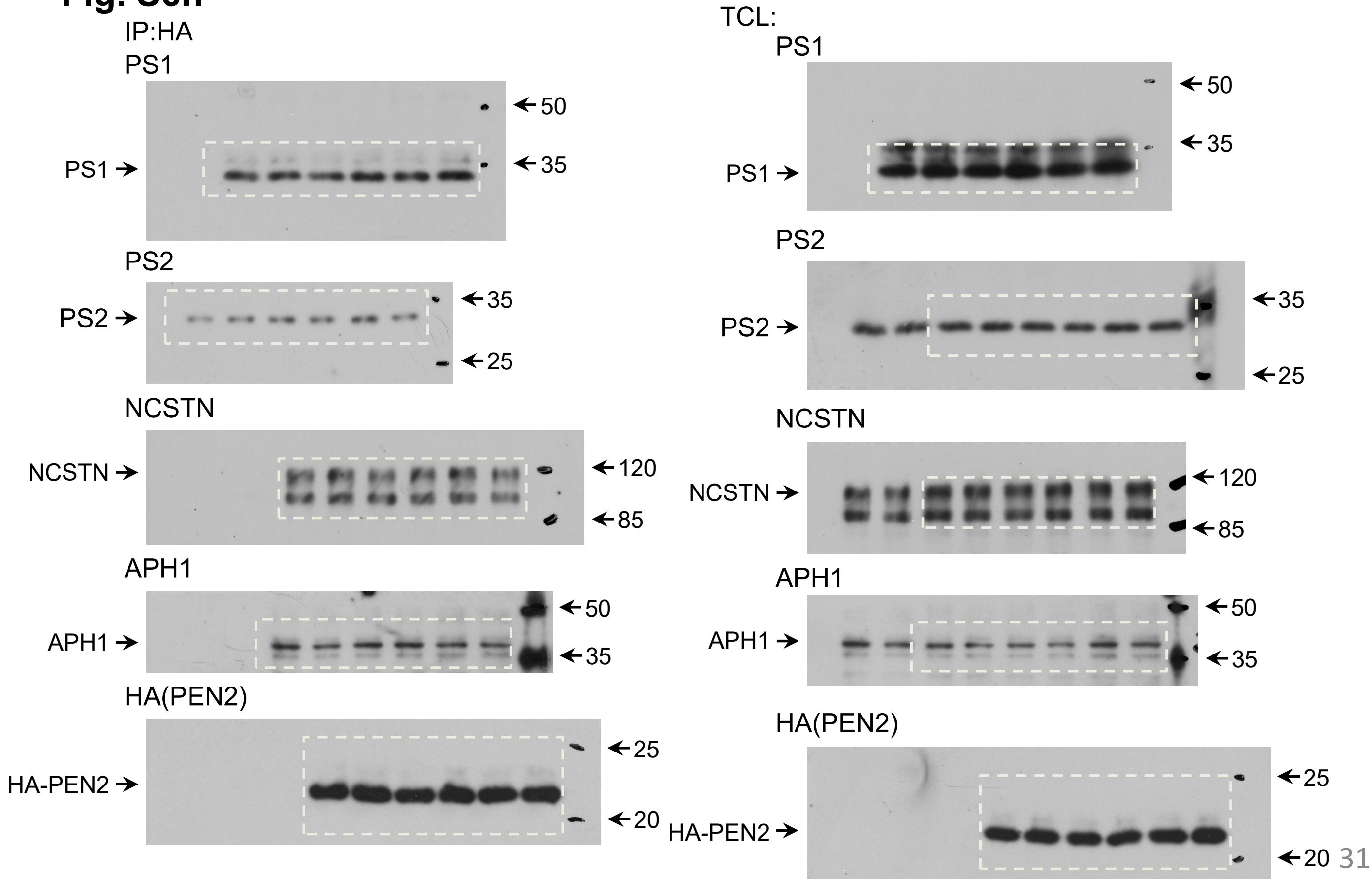

Fig. S6o

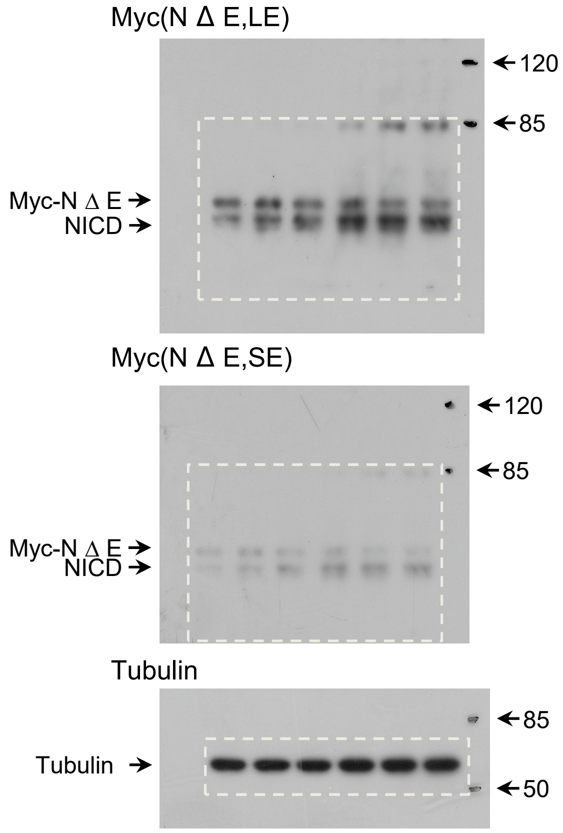

Fig. S7a

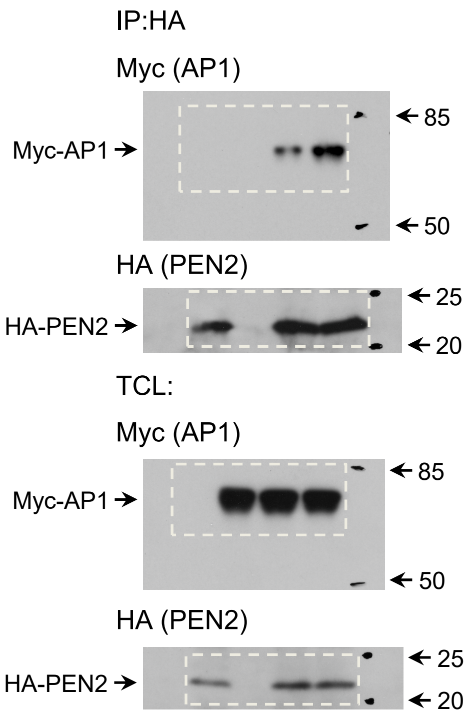

Fig. S7b

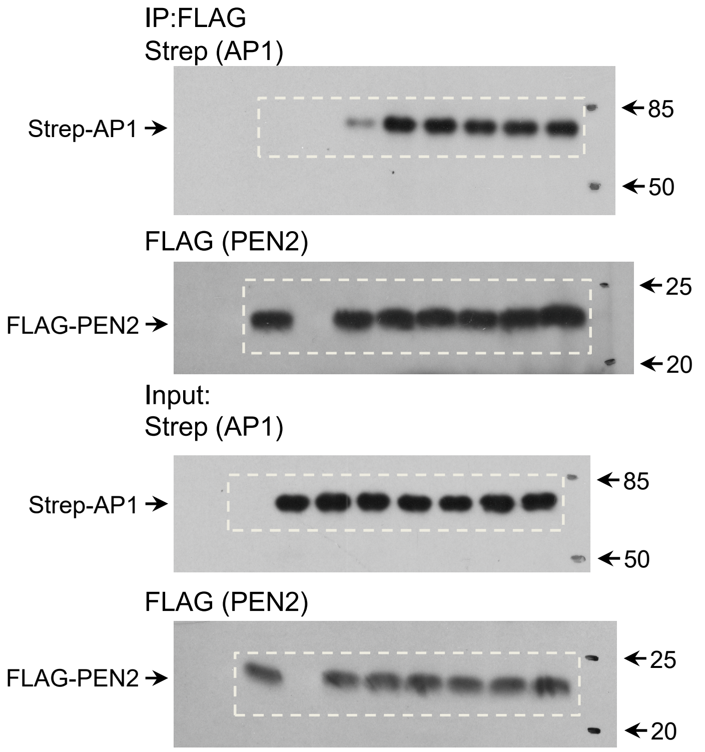

Fig. S7c

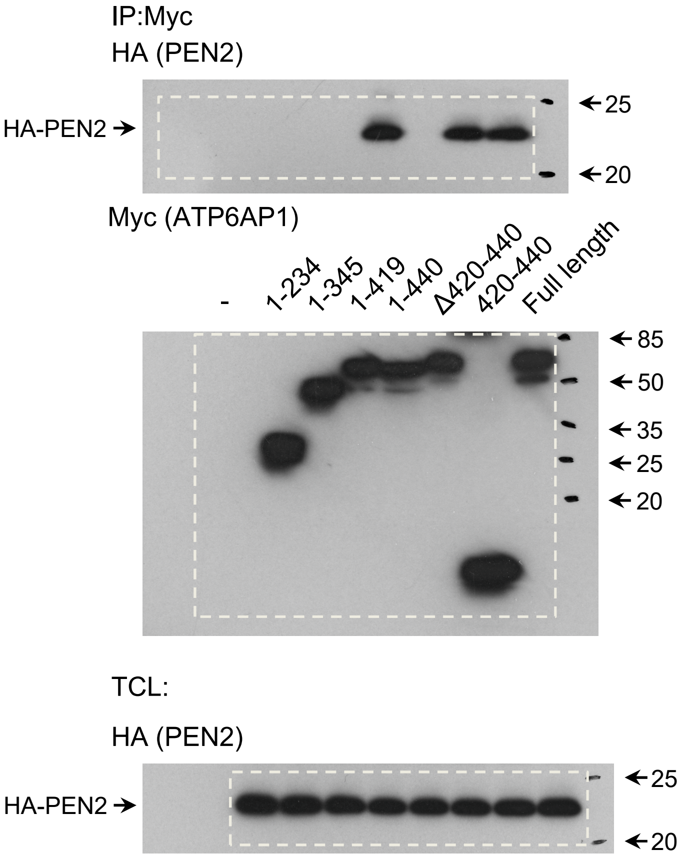

Fig. S7d

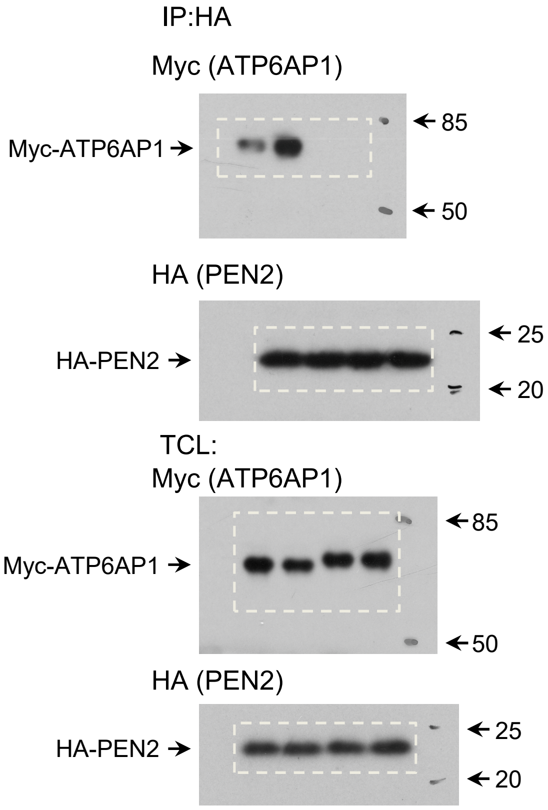

Fig. S7f

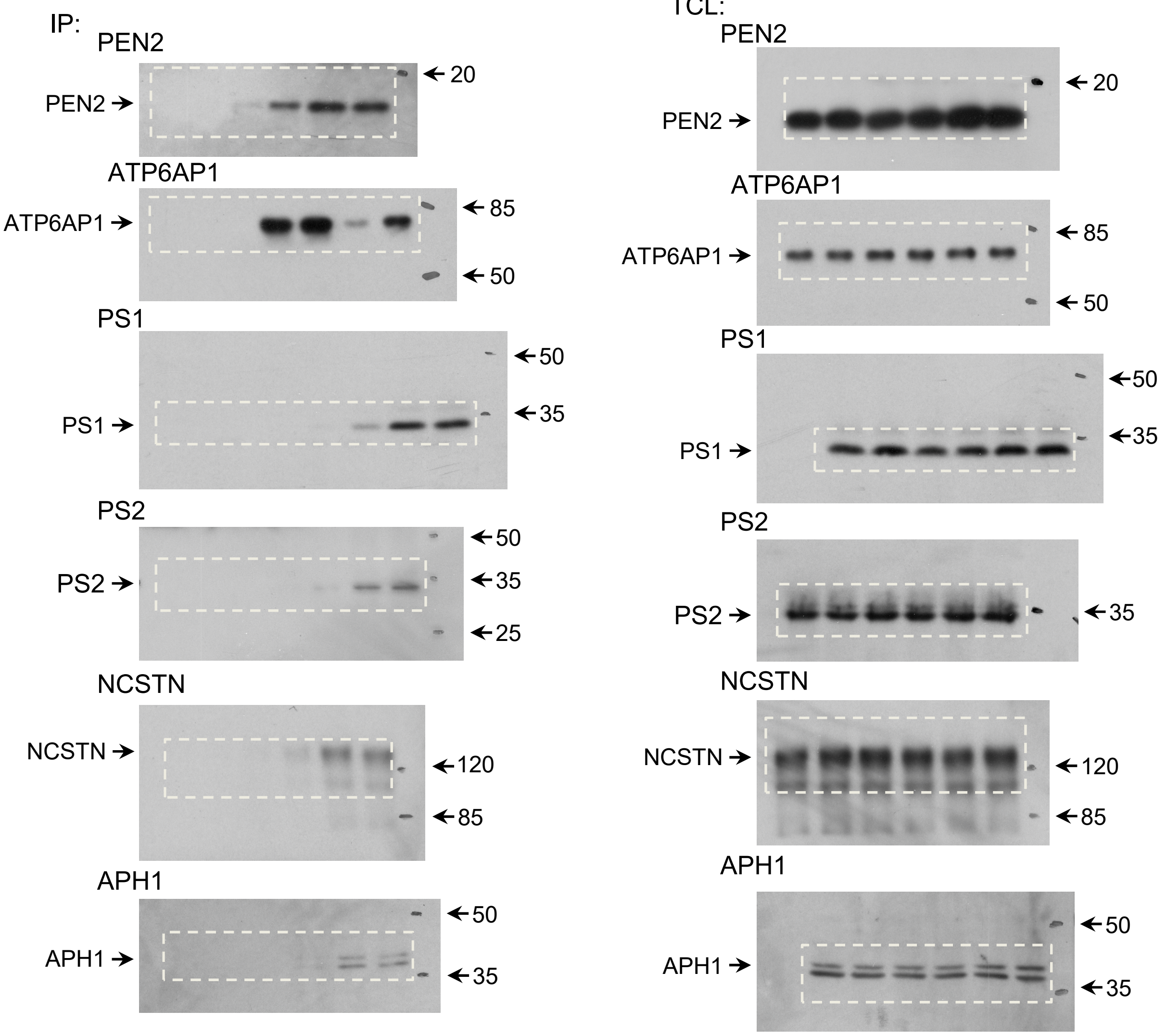

IP:
PEN2
PEN2
20
ATP6AP1
ATP6AP1
85
50
PS1
PS1
50
35
PS2
PS2
50
35
25
NCSTN
NCSTN
120
85
APH1
APH1
50
35
TCL:
PEN2
PEN2
20
ATP6AP1
ATP6AP1
85
50
PS1
PS1
50
35
PS2
PS2
35
NCSTN
NCSTN
120
85
APH1
APH1
50
35

Fig. S7g

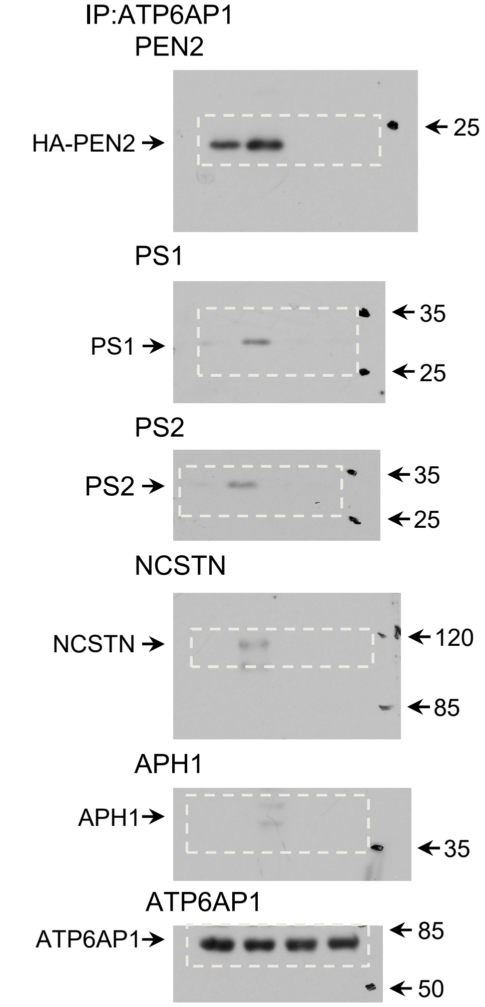

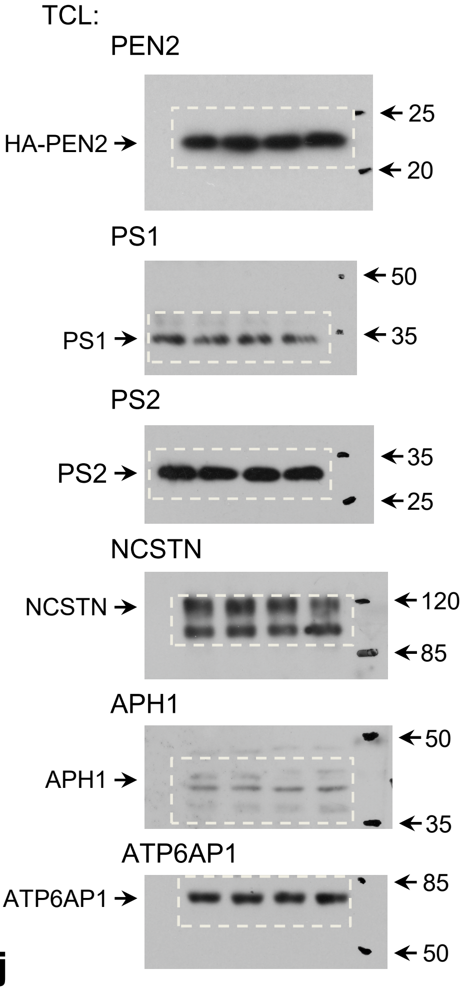

Fig. S7h

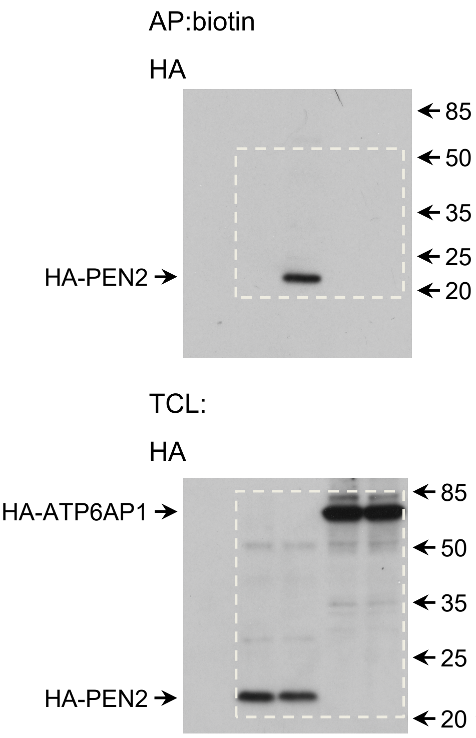

Fig. S7j

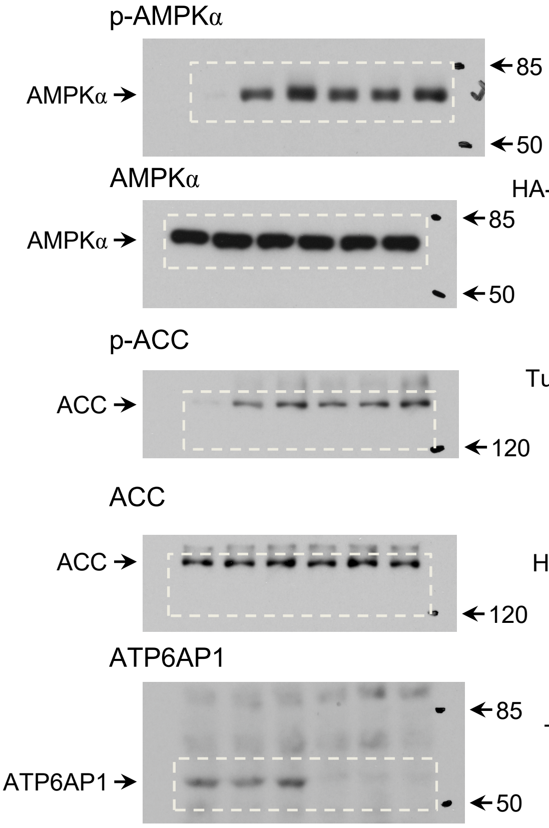

Fig. S7l upper

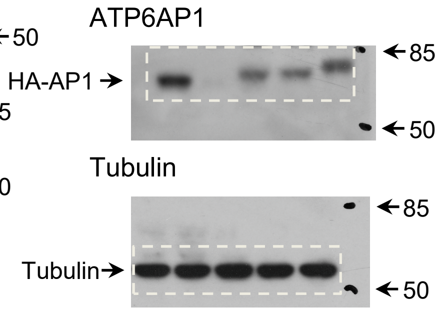

Fig. S7l lower

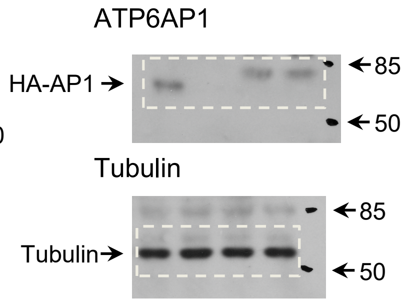

Fig. S8c left

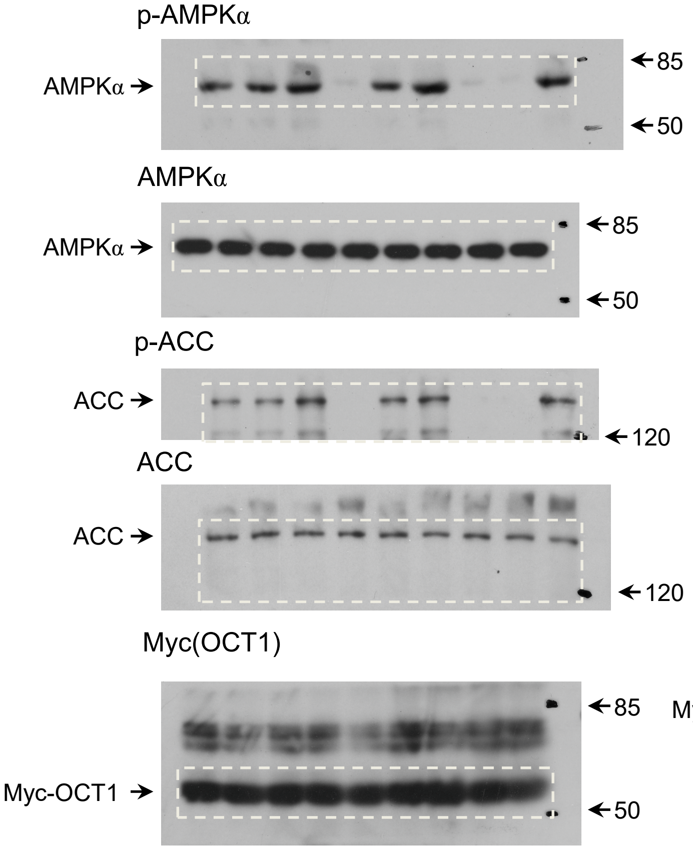

Fig. S8c right

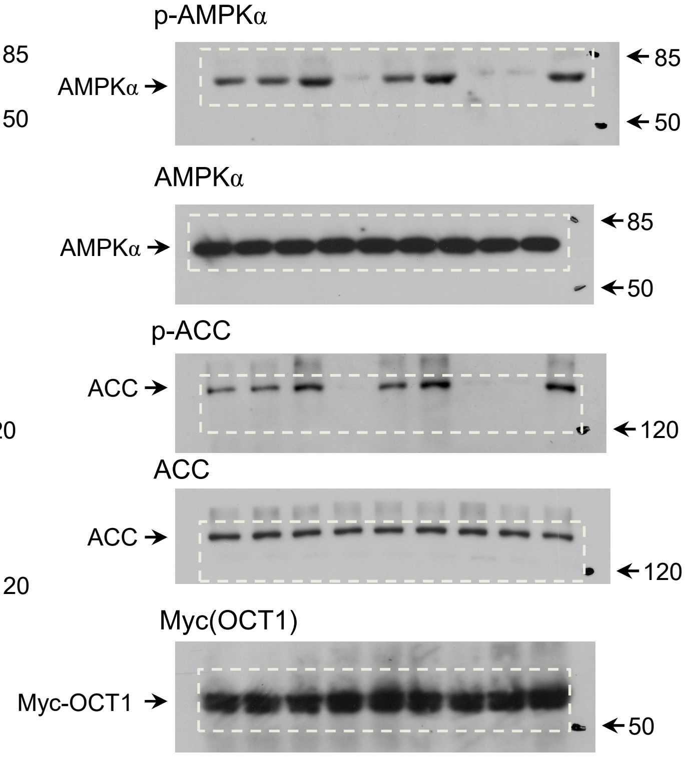

Fig. S8d

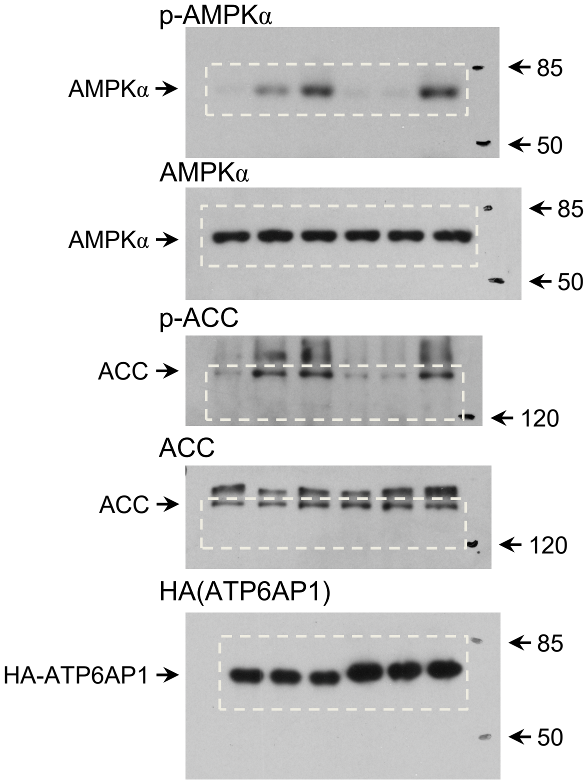

Fig. S9a

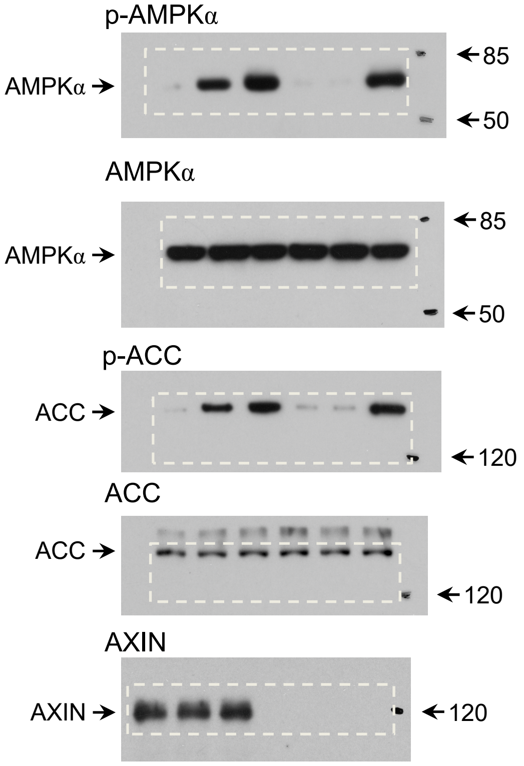

Fig. S9b

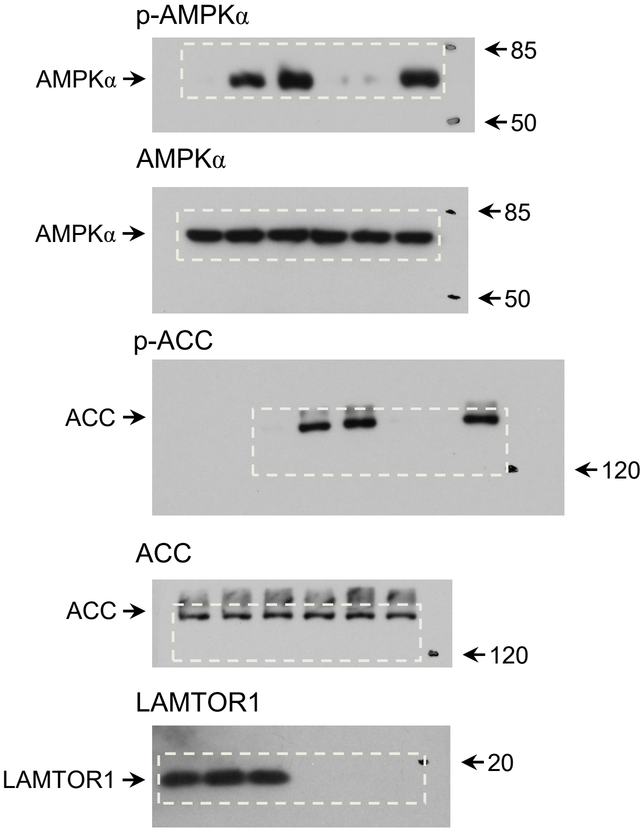

Fig. S9c

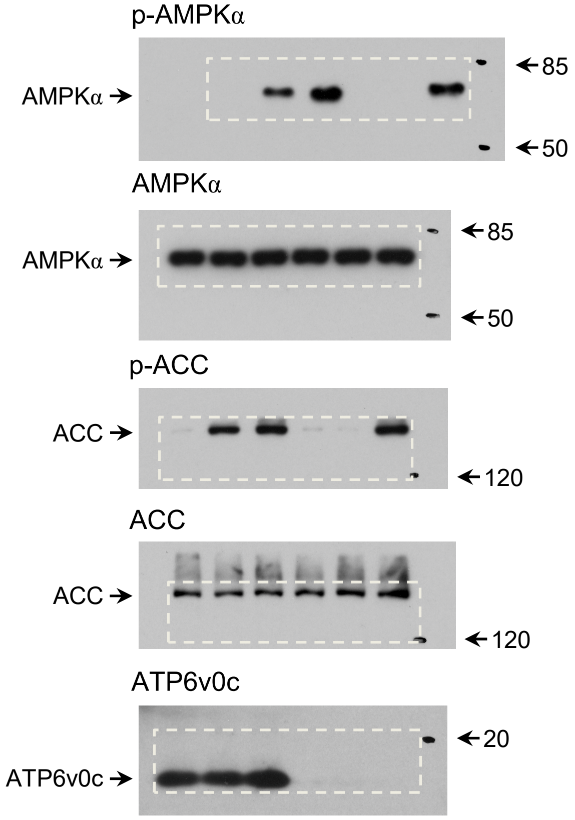

Fig. S9d

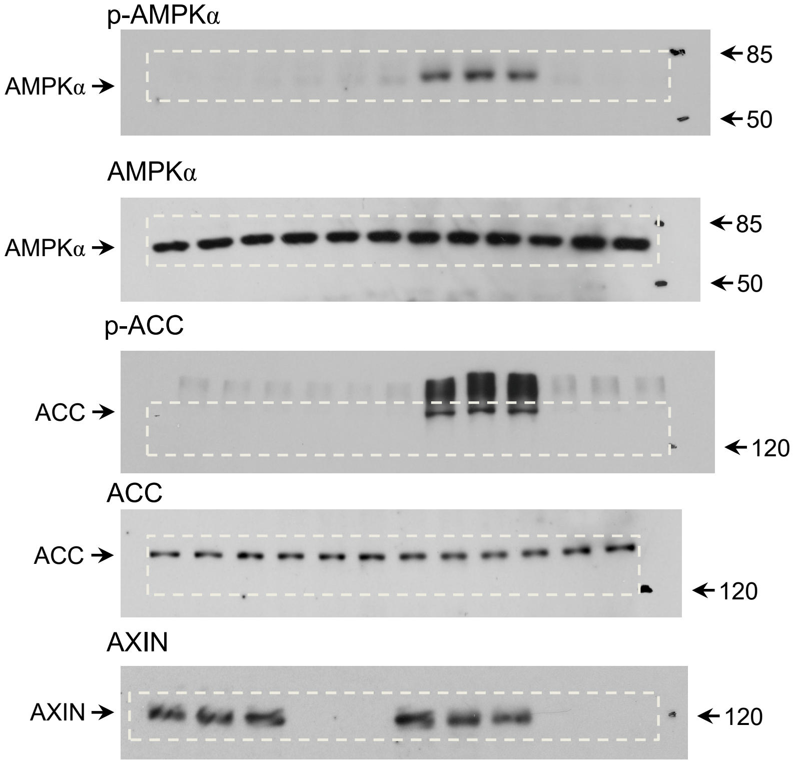

Fig. S9e

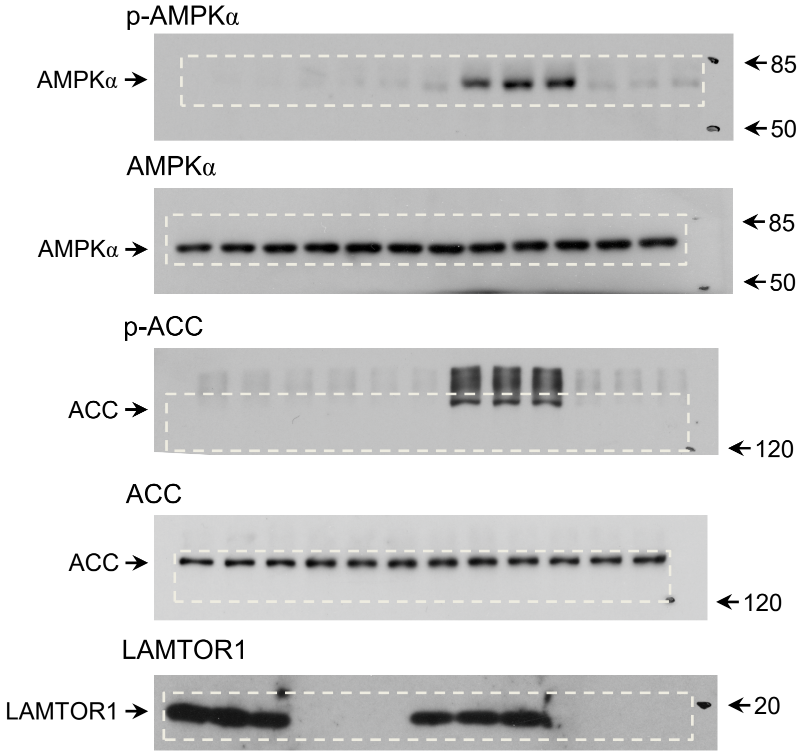

Fig. S9g

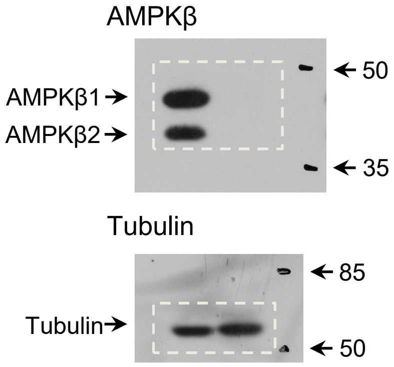

Fig. S9f

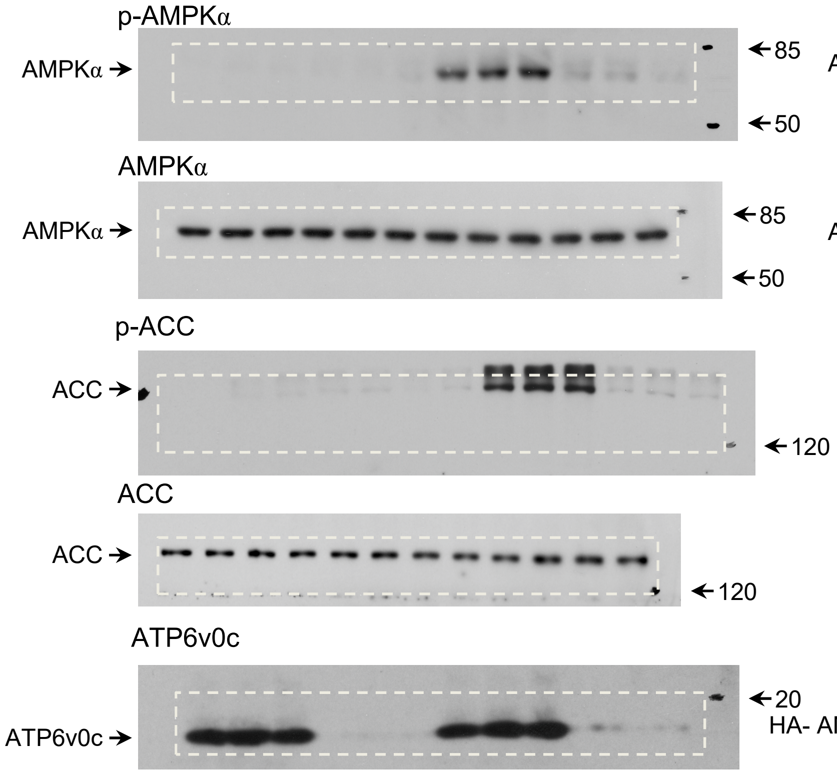

Fig. S9h

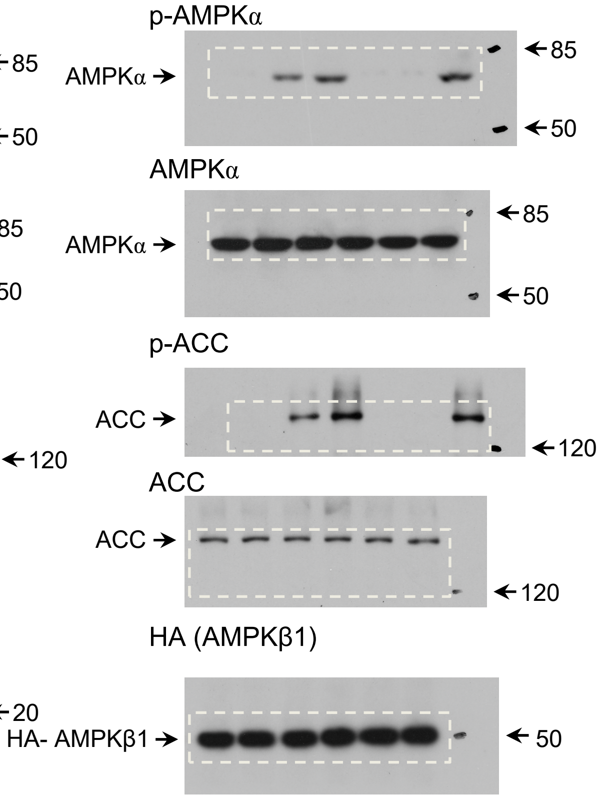

Fig. S9j

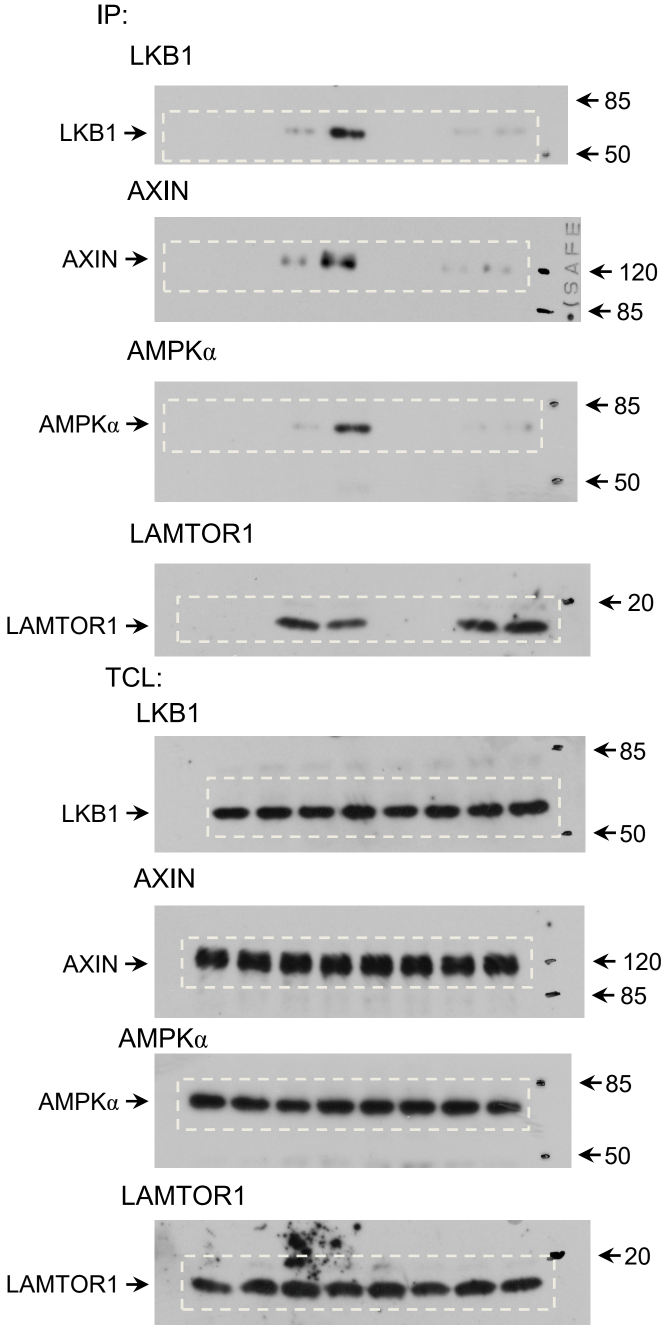

Fig. S9k

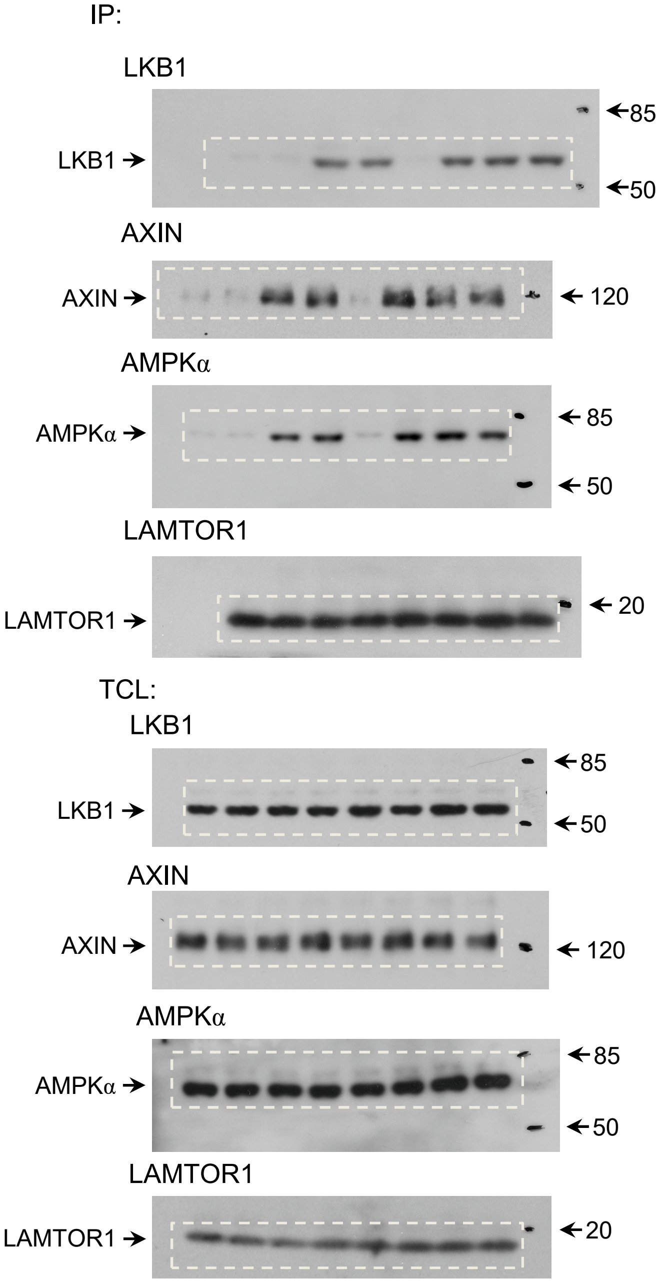

Fig. S9l

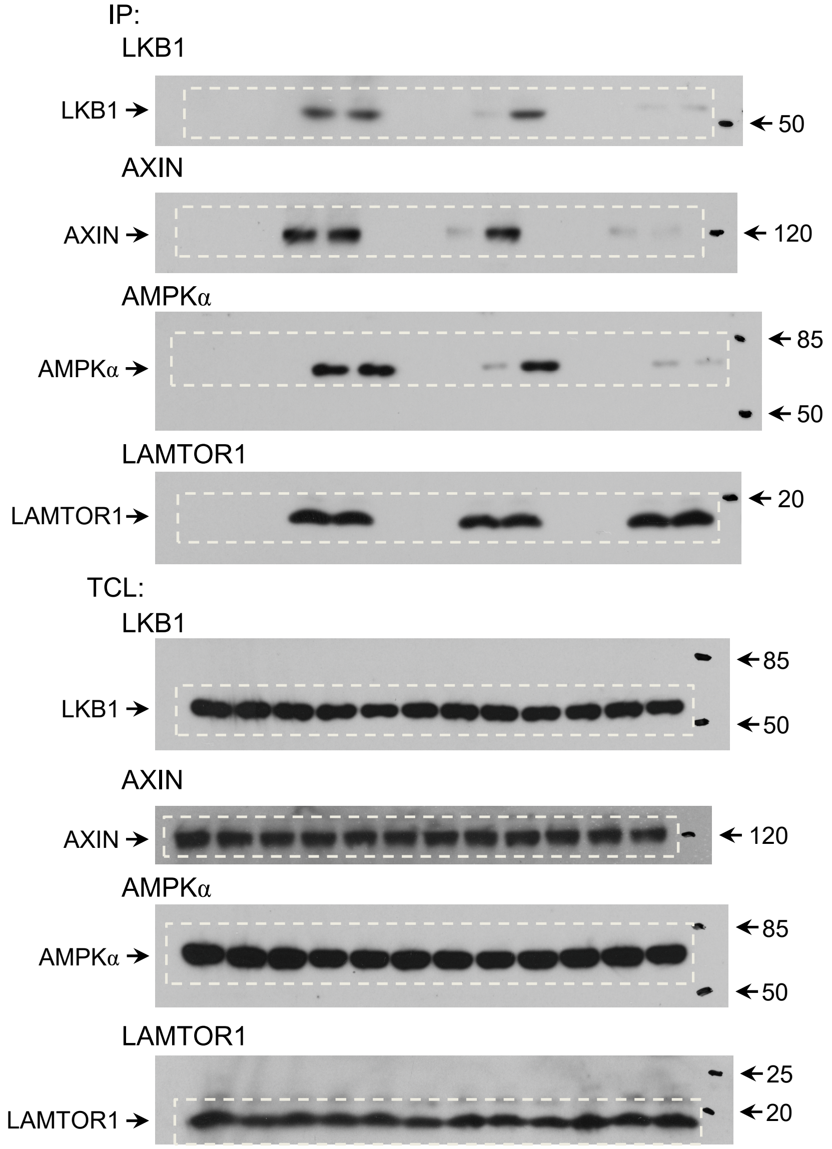

Fig. S11a

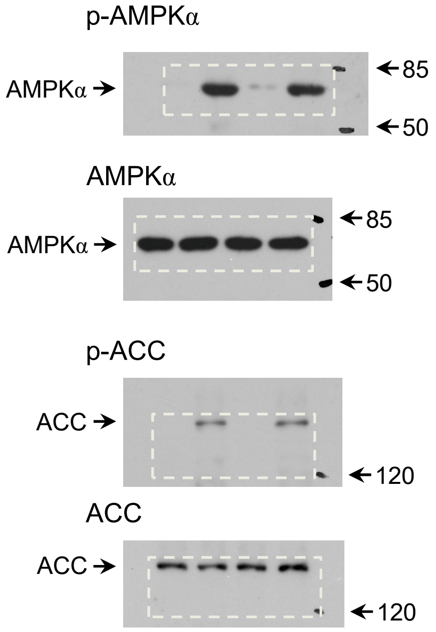

Fig. S11b

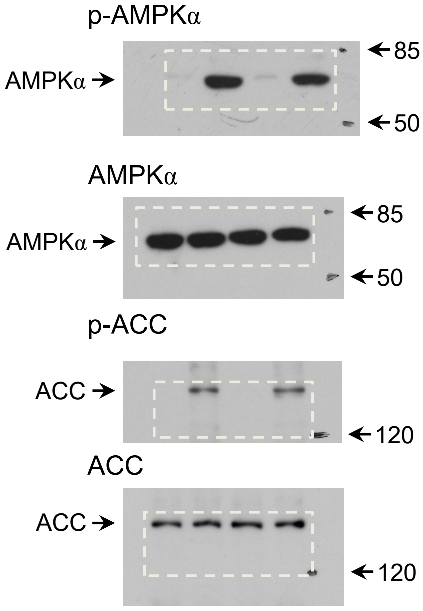

Fig. S11c

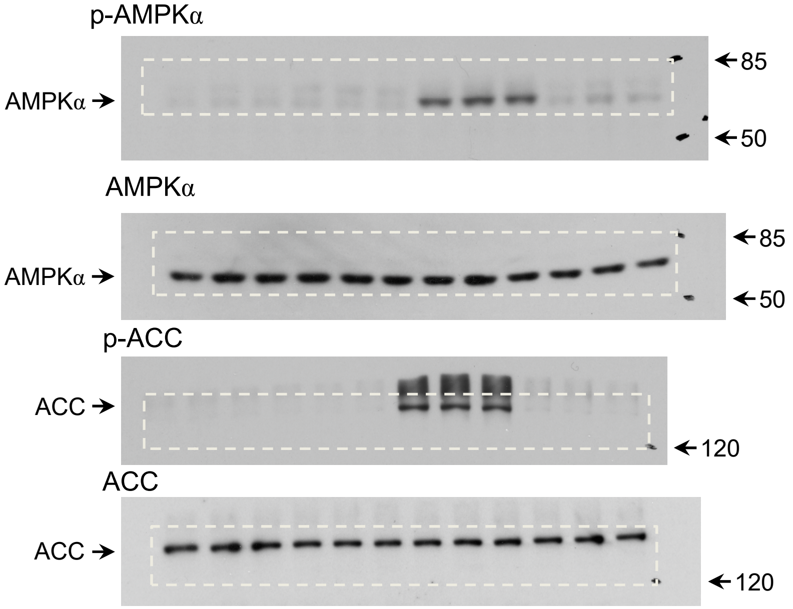

Fig. S11d

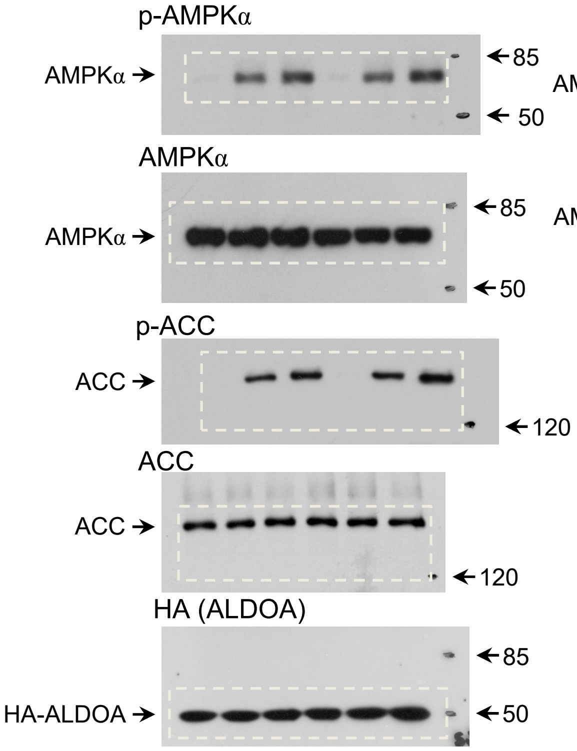

Fig. S11e

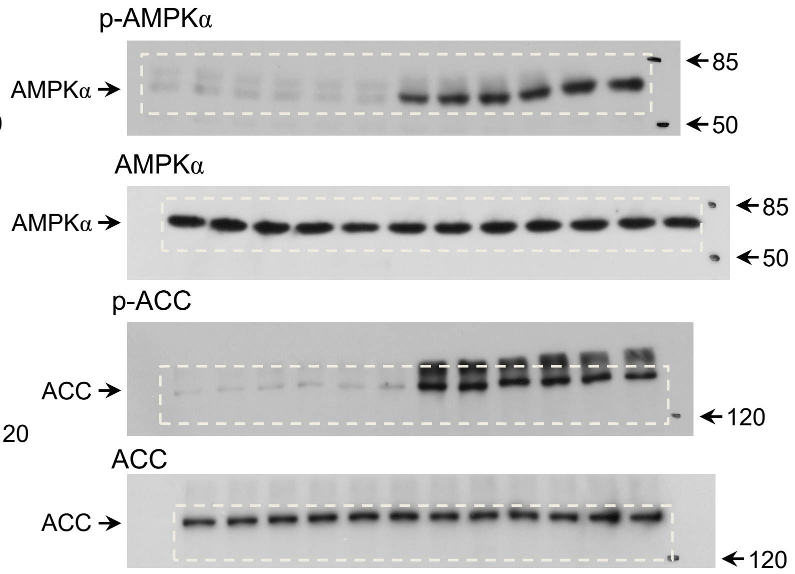

Fig. S11f

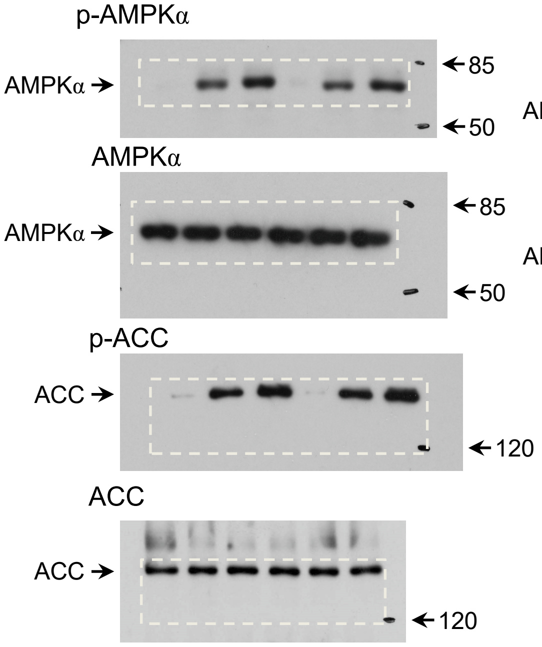

Fig. S11g

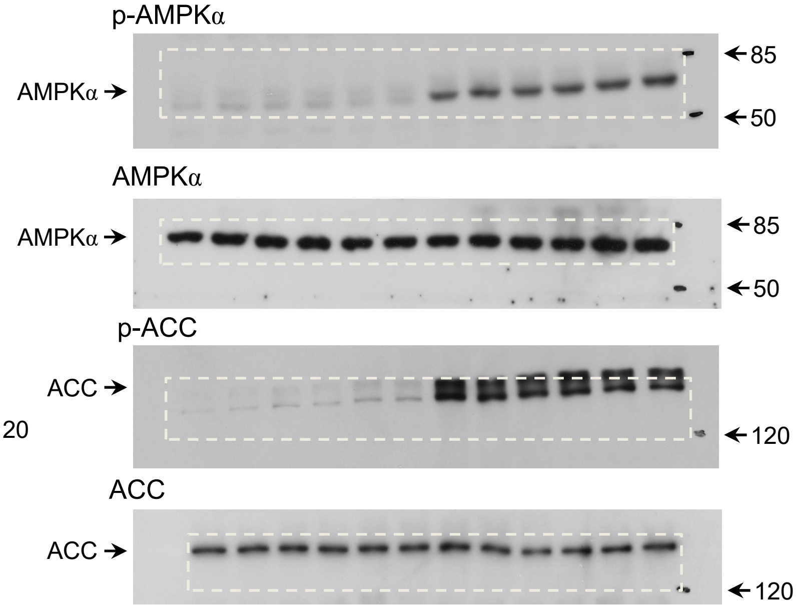

Fig. S12a

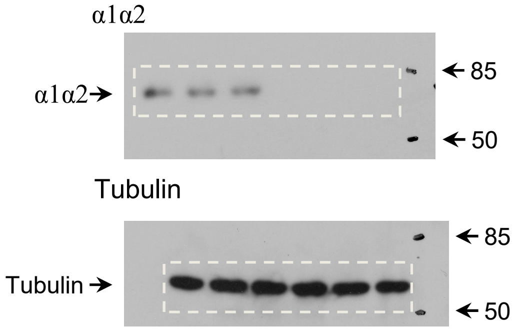

Fig. S12f

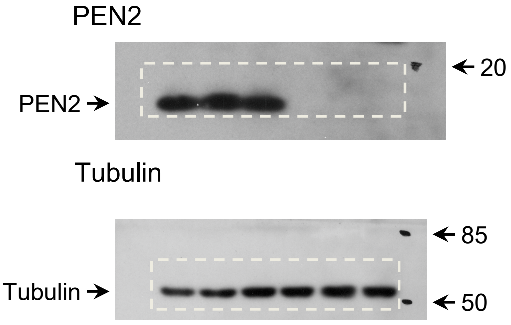

Fig. S12i right

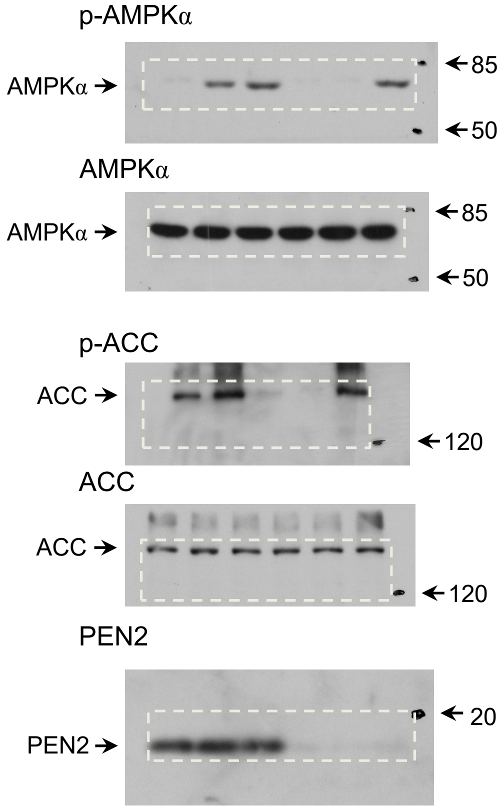

Fig. S12d

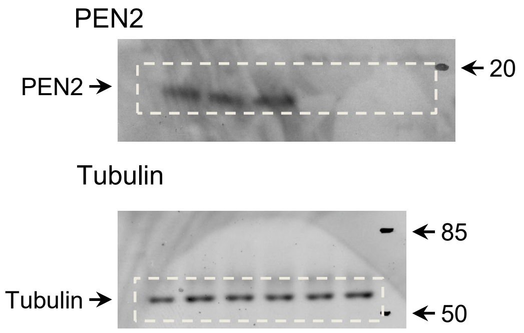

Fig. S12i left

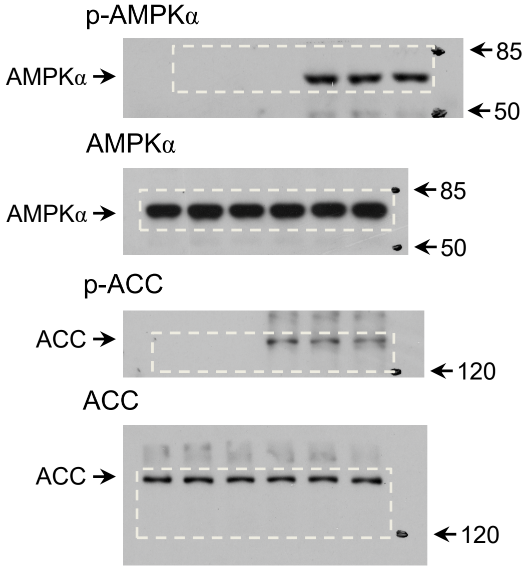

Fig. S12j

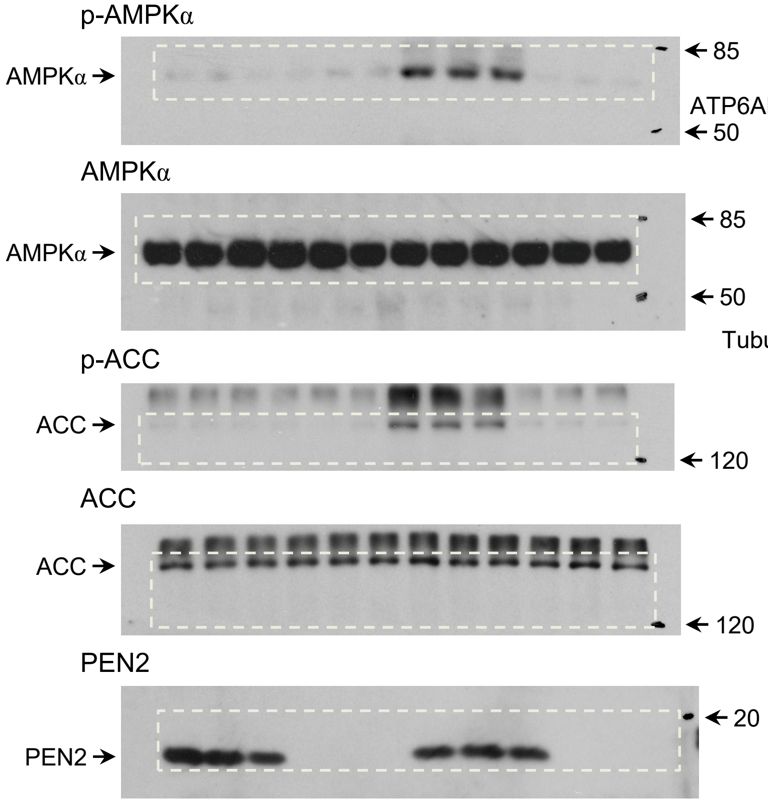

Fig. S12m

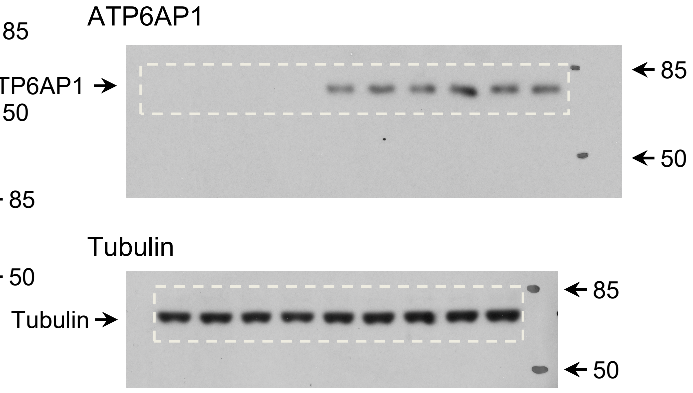

Fig. S12p

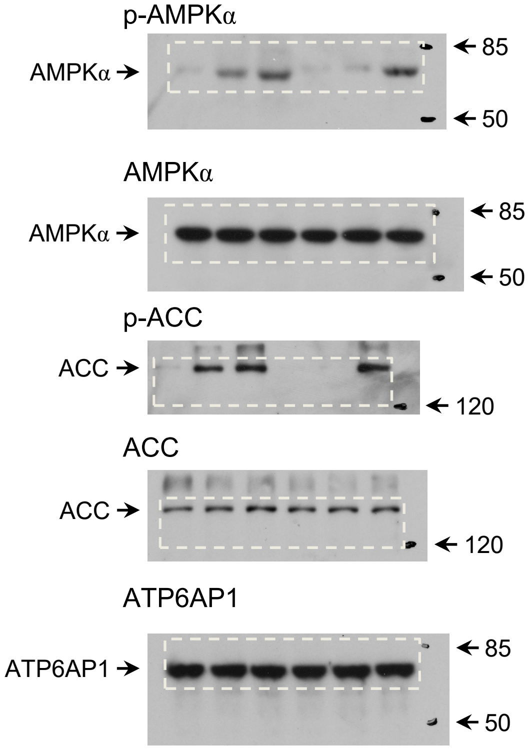

Fig. S12q

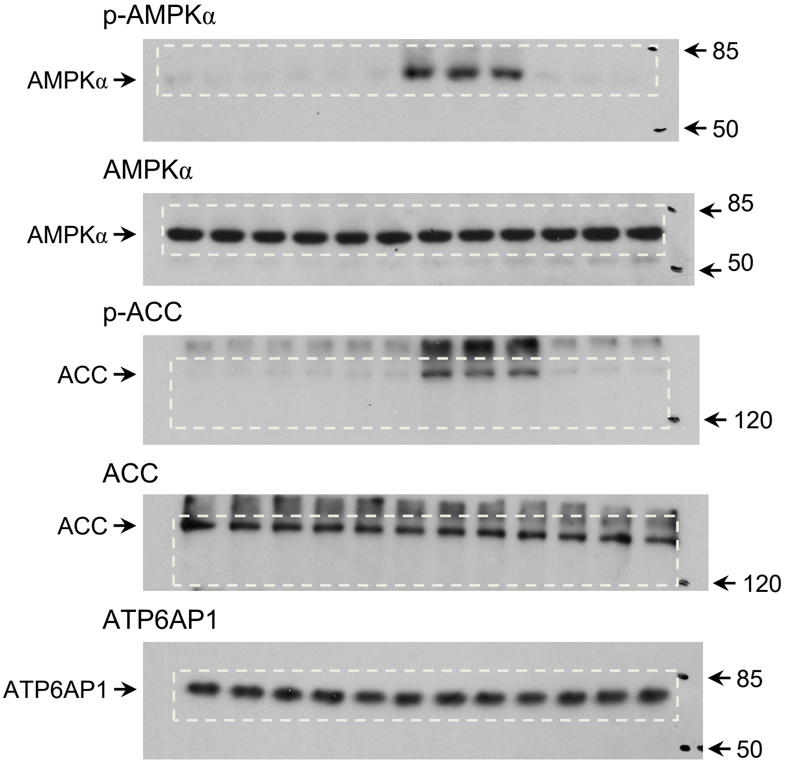

Fig. S13c

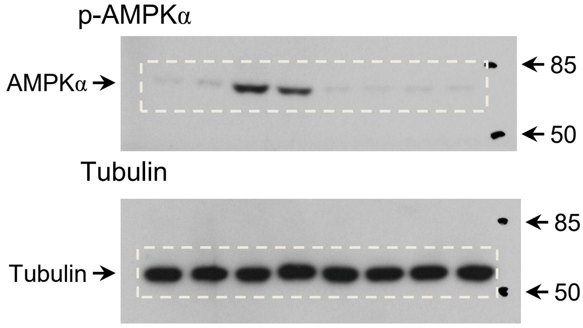

Fig. S13i

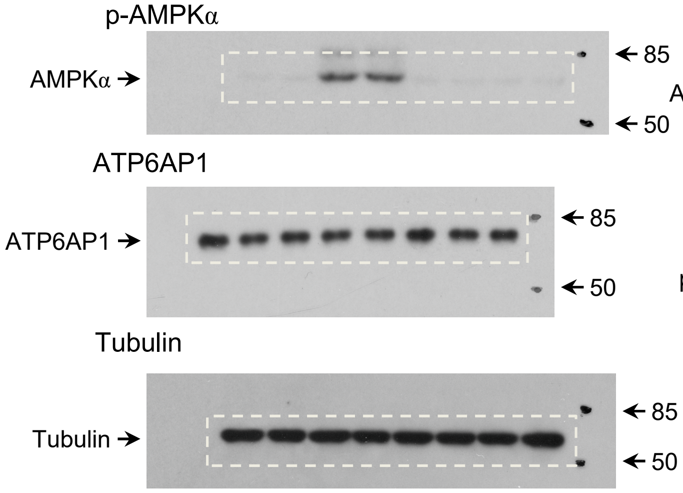

Fig. S14b

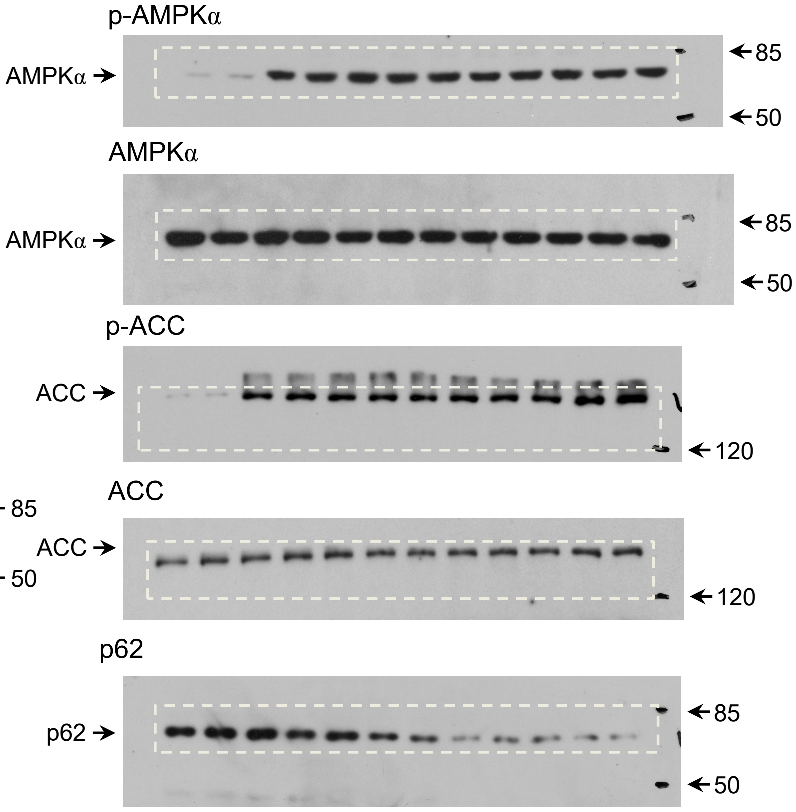

Fig. S14g left

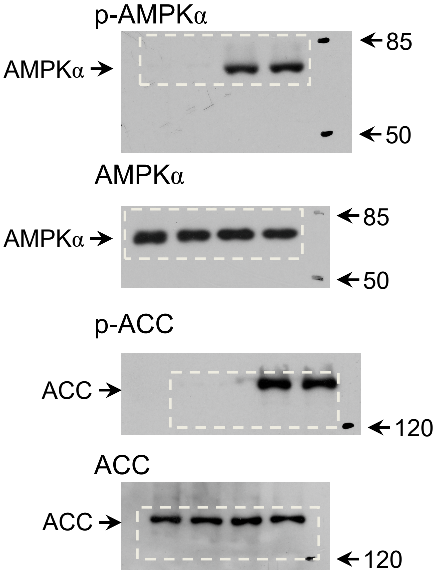

Fig. S14g middle

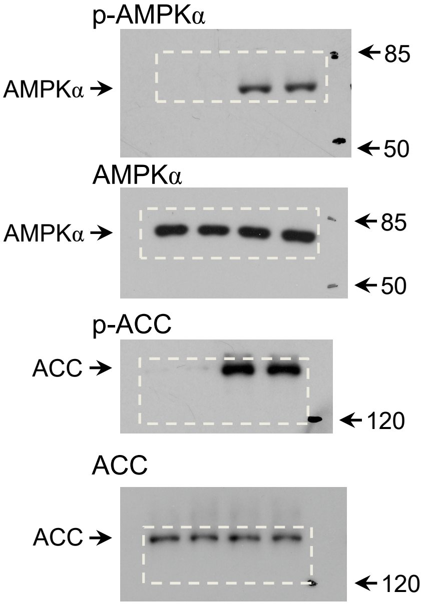

Fig. S14g right

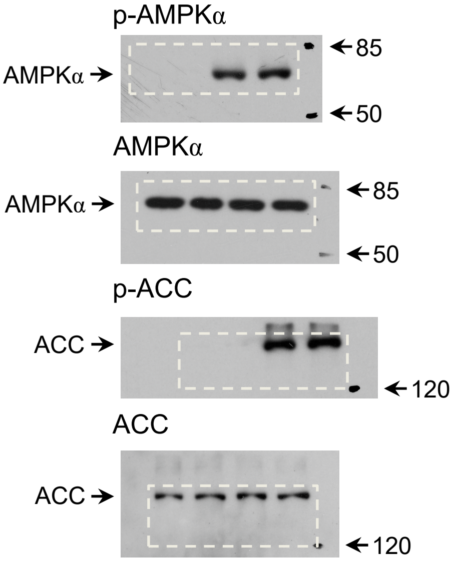

Fig. S14i

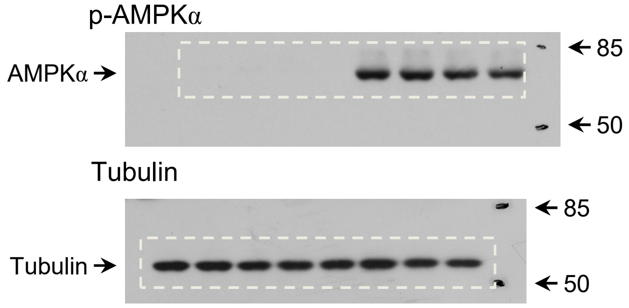

Fig. S14l upper

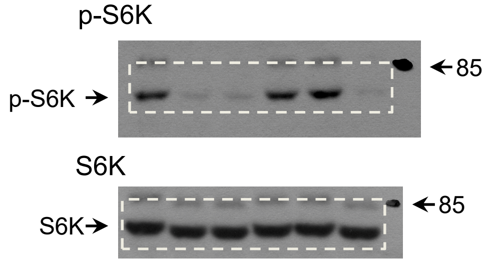

Fig. S14l middle

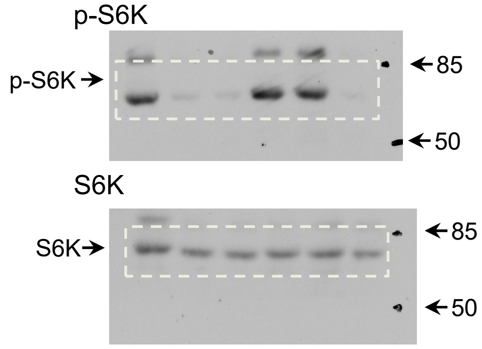

Fig. S14l lower

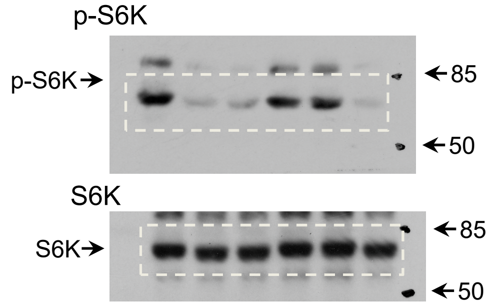

Fig. S14m upper

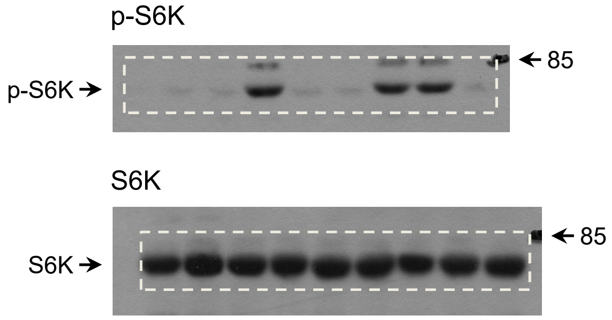

Fig. S14m middle

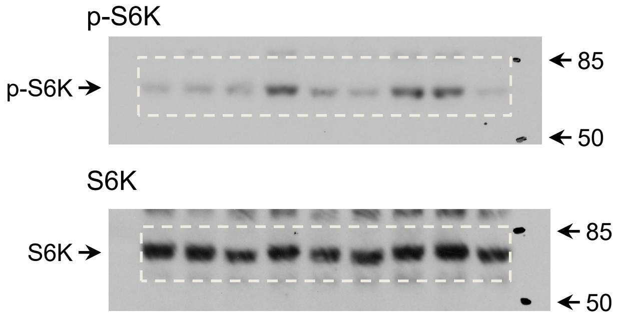

Fig. S14m lower

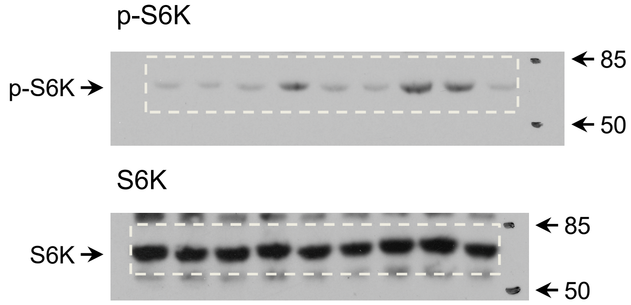

Fig. S14n

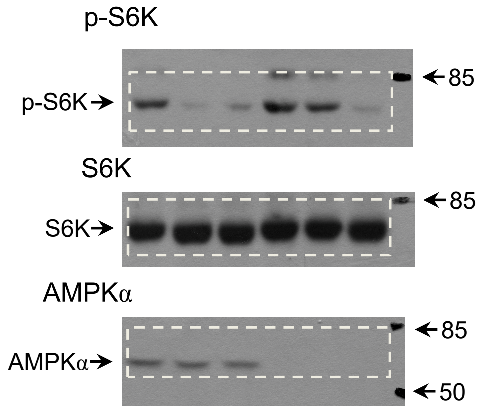

Supplementary Fig. 2 | NMR spectrums of Met-Ps. The ^{1}H NMR spectrum of Met-P1 (top left), ^{13}C NMR spectrum of Met-P1 (top right), ^{1}H NMR spectrum of Met-P2 (bottom left), and ^{13}C NMR spectrum of Met-P2 (bottom right) are shown. See also Extended Data Fig. 2a, c, and the "Synthesis of metformin probes" of Methods section.

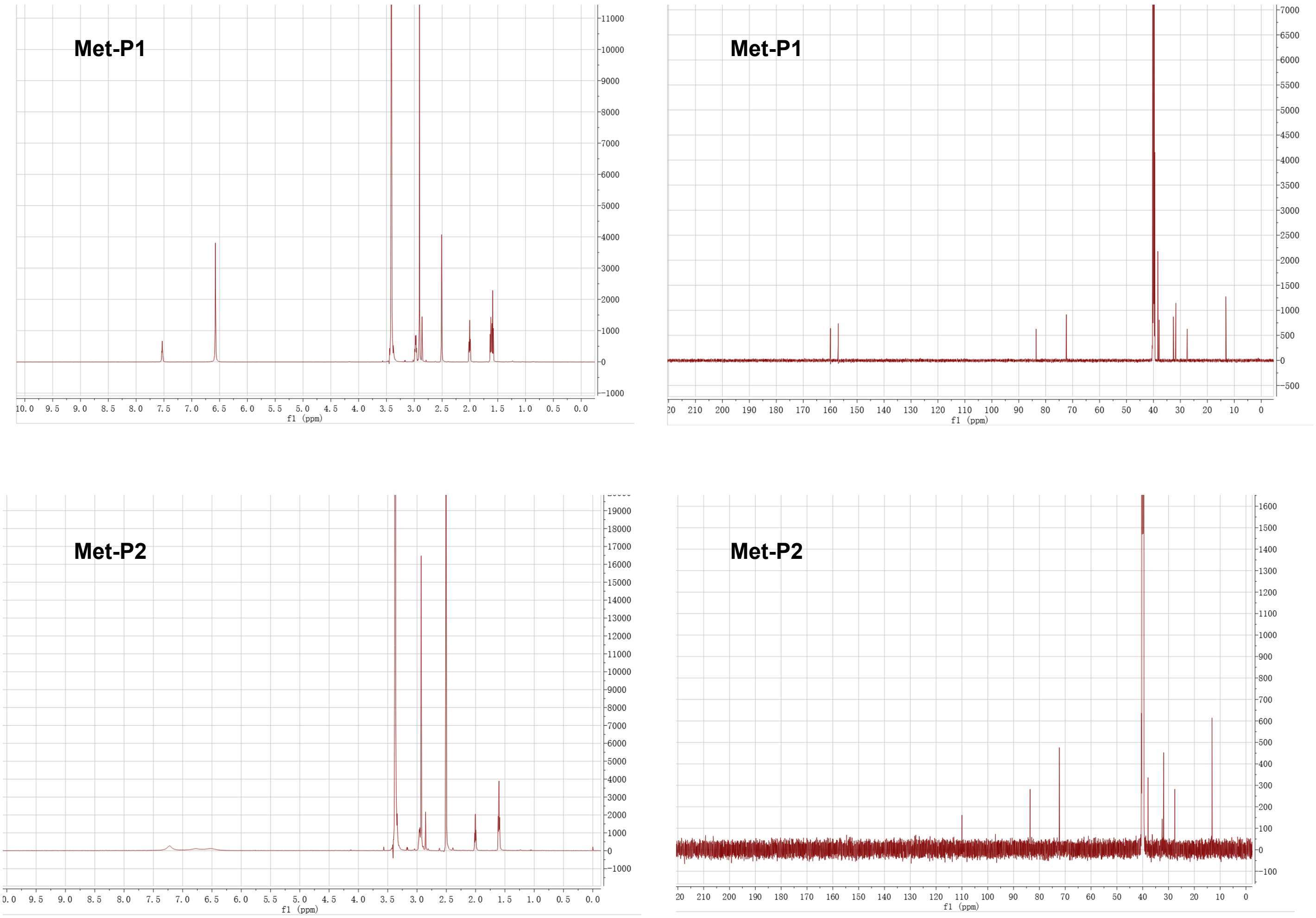

Supplement: Supplementary file 1 — This file contains Supplementary Notes 1–10, Supplementary References and Supplementary Fig. 1. [file 41586_2022_4431_MOESM1_ESM.pdf]
