# Supplementary material for: Low-dose metformin targets the lysosomal AMPK pathway through PEN2
Source: Nature. 2022 Feb 23;603(7899):159–65. doi: 10.1038/s41586-022-04431-8 (PMC8891018; doi:10.1038/s41586-022-04431-8)
Supplement: Supplementary file 2 — Reporting Summary [file 41586_2022_4431_MOESM2_ESM.pdf]

nature portfolio

Corresponding author(s): Xianming Deng, Chen-Song Zhang and Sheng-Cai Lin

Last updated by author(s): 2022/2/4

Reporting Summary

Nature Portfolio wishes to improve the reproducibility of the work that we publish. This form provides structure for consistency and transparency in reporting. For further information on Nature Portfolio policies, see our Editorial Policies and the Editorial Policy Checklist.

Statistics

For all statistical analyses, confirm that the following items are present in the figure legend, table legend, main text, or Methods section.

n/a	Confirmed	
☐	☒	The exact sample size (*n*) for each experimental group/condition, given as a discrete number and unit of measurement
☐	☒	A statement on whether measurements were taken from distinct samples or whether the same sample was measured repeatedly
☐	☒	The statistical test(s) used AND whether they are one- or two-sided<br>*Only common tests should be described solely by name; describe more complex techniques in the Methods section.*
☐	☒	A description of all covariates tested
☐	☒	A description of any assumptions or corrections, such as tests of normality and adjustment for multiple comparisons
☐	☒	A full description of the statistical parameters including central tendency (e.g. means) or other basic estimates (e.g. regression coefficient) AND variation (e.g. standard deviation) or associated estimates of uncertainty (e.g. confidence intervals)
☐	☒	For null hypothesis testing, the test statistic (e.g. *F*, *t*, *r*) with confidence intervals, effect sizes, degrees of freedom and *P* value noted<br>*Give P values as exact values whenever suitable.*
☒	☐	For Bayesian analysis, information on the choice of priors and Markov chain Monte Carlo settings
☒	☐	For hierarchical and complex designs, identification of the appropriate level for tests and full reporting of outcomes
☒	☐	Estimates of effect sizes (e.g. Cohen's *d*, Pearson's *r*), indicating how they were calculated

Our web collection on statistics for biologists contains articles on many of the points above.

Software and code

Policy information about availability of computer code

Data collection	EPSON Scan 3.9.3.4 was used to scan blots from X-ray films.<br>Zen 2012, LAS X (version 3.0.2.16120), NIS Elements software with STORM package (version 4.30 build 1053), and Hitachi TEM system control 01.20 were used to collect microscopic images, as described in related method sections.<br>VPViewer 2000 (version 2.66.7) was used to collect DSC data, ITC200 (version 1.26.1) to ITC, and BIAcore T200 Control software (version 2.0) to BIAcore.<br>OCR results were collected by Wave 2.6.1.<br>MassLynx 4.1 was used to monitor and collect data from preparative HPLC.<br>TopSpin (version 3.2) was used to collect NMR data, and Tune (version 2.1) to HRMS.<br>MassHunter LC/MS acquisition 10.1.48 was used to collect data of adenylate concentrations on CE-MS.<br>Analyst TF 1.6 was used to collect data of TAG levels on HPLC-MS, Analyst 1.6.3 for metformin, and Analyst 1.7.1 for adenylate concentrations on HPLC-MS.<br>MassHunter GC/MS acquisition B.07.04.2260 was used to collect data from GC-MS.
Data analysis	The band intensities of immunoblots and the intensity of CM-H2DCFDA in nematodes intestine were quantified using Image J software (version 1.8.0).<br>Plots were generated by Prism v9.<br>Statistical analysis was performed by Prism v9 and SPSS 27.0.<br>Microscopic images were analysed and processed by Zen 2012, Imaris 7.4.0, NIS Elements software with STORM package version 4.30 build 1053, and Hitachi TEM system control 01.20 as described in related Methods section, and were formatted on Photoshop 2021.<br>Results of metabolites measured on CE-MS were analysed on Qualitative Analysis B.06.00, and HPLC-MS on MultiQuant 3.0.3 (for adenylates) or MultiQuant 3.0.3 (for metformin).<br>Protein and peptide mass spectrometry data were analysed using Peaks Studio (version X+, for timsTOF Pro), ProteinPilot software (version

5.0, for TripleTOF 5600+), or Proteome Discoverer (version 2.2, for Orbitrap Fusion Lumos Tribrid).
RT-PCR results were analysed by Bio-Rad CFX Manager 3.1 (for C. elegans) or Roche LightCycler 96 1.1 (for hepatocytes) .
Preparative HPLC data were analysed by EasyChrom 2.0.
NMR data were analysed on MestReNova 9.0, and on HRMS by Xcalibur 2.2.
DSC and ITC results were analysed by Origin 7 (version 7.0552), and SPR results on BIAcore T200 Evaluation software (version 2.0).
OCR results were analysed by Wave 2.6.1.
Results of TAG measured on HPLC-MS were analysed by MS-DIAL 4.7, and TCA cycle intermediates by GC-MS MassHunter Workstation Software (version B.07.01SP1, Qualitative Analysis).

For manuscripts utilizing custom algorithms or software that are central to the research but not yet described in published literature, software must be made available to editors and reviewers. We strongly encourage code deposition in a community repository (e.g. GitHub). See the Nature Portfolio guidelines for submitting code & software for further information.

Data

Policy information about availability of data

All manuscripts must include a data availability statement. This statement should provide the following information, where applicable:

- Accession codes, unique identifiers, or web links for publicly available datasets
- A description of any restrictions on data availability
- For clinical datasets or third party data, please ensure that the statement adheres to our policy

The data supporting the findings of this study are available within the paper and its Supplementary Information files. The proteomics data have been deposited in the iProX integrated proteome resources (IPX0003776000). Materials, reagents or other experimental data are available upon reasonable request from the corresponding author. Full immunoblots are provided as Supplementary Information Fig. 1. Source data for all graphs are provided with this paper.
The analysis was performed using standard protocols with previously described computational tools. No custom code was used in this study.

Field-specific reporting

Please select the one below that is the best fit for your research. If you are not sure, read the appropriate sections before making your selection.

☒ Life sciences ☐ Behavioural & social sciences ☐ Ecological, evolutionary & environmental sciences

For a reference copy of the document with all sections, see nature.com/documents/nr-reporting-summary-flat.pdf

Life sciences study design

All studies must disclose on these points even when the disclosure is negative.

Sample size	The chosen sample sizes were similar to those previously used by us and others in this field: n = 4-6 human participants or mice were used to determine the pharmacokinetics of metformin (ref. 16,31,32); n = 5-7 mice were usually used to determine the effects of metformin on blood glucose (ref. 33,34) and fatty liver (ref. 20,35); n = 100-300 worms were used to determine lifespan (ref. 36,37); n = 20-42 cells from 2 - 6 experiments/dishes were included when conclusions were based on immunofluorescent staining (ref. 6,25); n = 3-5 samples were used for evaluation of the levels of AMP, ADP and ATP in cells and tissues (ref. 6,15,25,27); n = 3 samples to determine the expression levels and phosphorylation levels of a specific protein (ref. 24,38); n = 3 samples to determine the mRNA levels of a specific gene (ref. 20,24,39); and n = 3 samples to determine the activity of v-ATPase in vitro (ref. 25). No statistical methods were used to predetermine sample size.
Data exclusions	No data was excluded.
Replication	All experimental findings were repeated at least three times as stated in figure legends.
Randomization	Randomisation was applied wherever possible. For example, during MS analyses, samples were processed and subjected to the mass spectrometer in random orders. In animal experiments, sex-matched (only for mice), age-matched litter-mate mice in each genotype were randomly assigned to pharmacological or diet treatments. In cell experiments, cells of each genotype were parallel seeded and randomly assigned to different treatments. Otherwise, randomisation was not performed. For example, when performing immunoblotting, samples needed to be loaded in a specific order to generate the final figures.
Blinding	Blinding was applied wherever possible. For example, samples, cages or agar plates during sample collection and processing were labelled as code names that were later revealed by the individual who picked and treated animals or cells, but did not participate in sample collection and processing, until assessing outcome. Similarly, during microscopy data collection and statistical analyses, the fields of view were chosen on a random basis, and are often performed by different operators, preventing potentially biased selection for desired phenotypes. Otherwise, blinding was not performed, such as the measurement of v-ATPase activity, and the determination of metformin binding to PEN2 in vitro, where operators had to know the conditions of each well and added reagents to the well accordingly during the measurement.

Reporting for specific materials, systems and methods

We require information from authors about some types of materials, experimental systems and methods used in many studies. Here, indicate whether each material, system or method listed is relevant to your study. If you are not sure if a list item applies to your research, read the appropriate section before selecting a response.

Materials & experimental systems

n/a	Involved in the study
☐	☒ Antibodies
☐	☒ Eukaryotic cell lines
☒	☐ Palaeontology and archaeology
☐	☒ Animals and other organisms
☐	☒ Human research participants
☐	☒ Clinical data
☒	☐ Dual use research of concern

Methods

n/a	Involved in the study
☒	☐ ChIP-seq
☒	☐ Flow cytometry
☒	☐ MRI-based neuroimaging

Antibodies

Antibodies used

Rabbit polyclonal antibody against LAMTOR1 was raised and validated as described previously (ref. 24), and was diluted 1:100 for immunoprecipitation (IP) or 1:500 for immunoblotting (IB). Rabbit polyclonal antibody against ATP6AP1 was raised with bacterially expressed and purified ATP6AP1 (aa 440-470, GST-tagged), and was diluted 1:100 for IP. Rabbit anti-phospho-AMPKα-T172 (cat. #2535, 1:1,000 for IB), anti-AMPKα (cat. #2532, 1:1,000 for IB), anti-AMPKβ1/2 (cat. #4150, 1:1,000 for IB), anti-phospho-ACC-Ser79 (cat. #3661, 1:1,000 for IB), anti-ACC (cat. #3662, 1:1,000 for IB), anti-phospho-p70 S6K-S389 (cat. #9234, 1:1000 for IB), anti-p70 S6K (cat. #2708, 1:1000 for IB), anti-LKB1 (cat. #3047, 1:1,000 for IB), anti-AXIN1 (cat. #2074, 1:1,000 for IB), anti-presenilin 1 (cat. #3622, 1:1,000 for IB), anti-presenilin 2 (cat. #2192, 1:1,000 for IB), anti-nicastrin (cat. #3632, 1:1,000 for IB), anti-β-tubulin (cat. #2128, 1:1,000 for IB), anti-HA-tag [(cat. #3724, 1:1,000 for IB or 1:120 for immunofluorescent staining (IF)], anti-PDI (cat. #3501, 1:1,000 for IB), anti- cytochrome c (cat. #4280, 1:1,000 for IB), anti-clathrin (cat. #4796, 1:1,000 for IB), anti-p62 (cat. #23214, 1:1,000 for IB), anti-ATG5 (cat. #12994, 1:1,000 for IB), anti-PDI (Alexa Fluor 488-conjugated, cat. #5051, 1:60 for IF), HRP-conjugated mouse anti-rabbit IgG (conformation specific, cat. #5127, 1:2,000 for IB), HRP-conjugated goat anti-rat IgG (conformation specific, cat. #98164, 1:2,000 for IB) and mouse anti-Myc-tag (cat. #2276, 1:500 for IB) antibodies were purchased from Cell Signaling Technology. Rabbit anti-ATP6v0c (cat. NBP1-59654, 1:1,000 for IB or 1:100 for IP) antibody was purchased from Novus Biologicals. Mouse ANTI-FLAG® M2 (cat. F1804, 1:1,000 for IB), goat anti-rabbit IgG antibody and ANTI-FLAG® M2 Affinity Gel (cat. A2220, 1:500 for IP) were purchased from Sigma. Rabbit anti-PEN2 (cat. ab154830, 1:1,000 for IB or 1:100 for IP and IF), anti-ATP6AP1 (cat. ab176609, 1:500 for IB), anti-transferrin (cat. ab1223, 1:500 for IB), anti-TGN46 (cat. ab76282, 1:60 for IF), and rat anti-LAMP2 (cat. ab13524; 1:1000 for IB or 1:120 IF) antibodies were purchased from Abcam. Goat anti-AXIN (cat. sc-8567, 1:120 for IF), mouse anti-HA (cat. sc-7392, 1:1,000 for IB, 1:500 for IP or 1:120 for IF), mouse anti-goat IgG-HRP antibody were purchased from Santa Cruz Biotechnology. Normal rabbit control IgG (cat. CR1, 1:100 for IP) was purchased from Sino Biological. Goat anti-mouse IgG (cat. 115-035-003, 1:1,000 for IB) and anti-rabbit (cat. 111-035-003, 1:1,000 for IB) antibodies were purchased from Jackson ImmunoResearch. Donkey anti-goat IgG (cat. #A-11055, 1:1000 for IB), anti-rabbit IgG (cat. #A-21206, 1:1000 for IB), anti-rat IgG (cat. #A21209, 1:1,000 for IB), goat anti-rat IgG (cat. #A-21247, 1:1,000 for IB), rabbit anti-APH1 (cat. #PA1-2010, 1:1,000 for IB), mouse anti-Strep-tag (cat. #MA5-17283, 1:1,000 for IB) antibodies were purchased from Thermo Scientific. Rabbit anti-ATP1A1 (cat. #14418-1-AP, 1:60 for IF) and anti-TOMM20 (cat. #11802-1-AP, 1:60 for IF) antibodies were purchased from Proteintech.

Validation

Rabbit polyclonal antibody against LAMTOR1 was validated as described previously (ref. 6). The in-house generated rabbit polyclonal antibody against ATP6AP1 (for IP), as well as the rabbit anti-ATP6AP1 (cat. ab176609, validated by the manufacturer: https://www.abcam.com/atp6ap1atp6s1-antibody-ab176609.html) purchased from Abcam was validated in this study using ATP6AP1 knockout cell lines/mouse strains. Rabbit anti-PEN2 (cat. ab154830) was validated by the manufacturer: https://www.abcam.com/pen2-antibody-epr9200-ab154830.html, and also in this study using PEN2 knockout cell lines/mouse strains.

The following commercially available antibodies were validated by the company, as well as other researchers (as the information collected by the RRID database):
Rabbit anti-phospho-AMPKα-T172 (cat. #2535, RRID: AB_331250), anti-AMPKα (cat. #2532, RRID: AB_330331), anti-AMPKβ1/2 (cat. #4150, RRID: AB_10828832), anti-phospho-ACC-Ser79 (cat. #3661, RRID: AB_330337), anti-ACC (cat. #3662, RRID: AB_2219400), anti-phospho-p70 S6K-S389 (cat. #9234, AB_2269803), anti-p70 S6K (cat. #2708, RRID:AB_390722), anti-LKB1 (cat. #3047, RRID: AB_2198327), anti-AXIN1 (cat. #2074, RRID: AB_2062419), anti-Presenilin 1 (cat. #3622, RRID: AB_2172895), anti-Presenilin 2 (cat. #2192, RRID: AB_2170609), anti-Nicastrin (cat. #3632, RRID: AB_2149581), anti-β-tubulin (cat. #2128, RRID: AB_823664), anti-HA-tag (cat. #3724, RRID: AB_1549585), anti-PDI (cat. #3501, RRID: AB_2156433), anti- Cytochrome c (cat. #4280, RRID: AB_10695410), anti-Clathrin (cat. #4796, RRID: AB_10828486), anti-p62 (cat. #23214, RRID:AB_2798858), anti-ATG5 (cat. #12994, RRID:AB_2630393), anti-PDI (Alexa Fluor 488-conjugated, cat. #5051, RRID:AB_10950503),and mouse anti-Myc-tag (cat. #2276, RRID: AB_331783) by Cell Signaling Technology; rabbit anti-ATP6v0c (cat. NBP1-59654, RRID: AB_11004830) antibody by Novus Biologicals; mouse ANTI-FLAG® M2 (cat. F1804, RRID: AB_262044), ANTI-FLAG® M2 Affinity Gel (cat. A2220, RRID: AB_10063035), and Atto 488 goat anti-rabbit IgG (cat. 18772, RRID: AB_1137637) antibodies by Sigma; rabbit anti-Transferrin (cat. Ab1223, RRID: AB_298951), anti-TGN46 (cat. ab76282, RRID:AB_1524486), and rat anti-LAMP2 (cat. ab13524; RRID: AB_369111) antibodies by Abcam; goat anti-AXIN (cat. sc-8567, RRID: AB_2227789), mouse anti-HA (cat. sc-7392, RRID: AB_627809) by Santa Cruz Biotechnology; Alexa Fluor 488 donkey anti-goat IgG (cat. #A-11055, RRID: AB_2534102), Alexa Fluor 488 donkey anti-rabbit IgG (cat. #A-21206, RRID: AB_2535792), Alexa Fluor 594 donkey anti-rat IgG (cat. #A21209, RRID: AB_2535795), Alexa-Fluor 647 goat anti-rat IgG (cat. #A-21247, RRID: AB_141778), rabbit anti-APH1 (cat. #PA1-2010, RRID: AB_2227105), mouse anti-Strep-tag (cat. #MA5-17283, RRID: AB_2538749) antibodies by Thermo; Rabbit anti-ATP1A1 (cat. #14418-1-AP, RRID:AB_2227873) and anti-TOMM20 (cat. #11802-1-AP, RRID:AB_2207530) antibodies by Proteintech.

Eukaryotic cell lines

Policy information about cell lines

Cell line source(s)

HEK293T, and L929 (NCTC clone 929) cells were purchased from ATCC. AD293 (Adeno-X 293) cells were purchased from

	Clontech. MEFs and primary hepatocytes were obtained from the indicated mouse strains. Suspension HEK293T cell line was established from the HEK293T cells and a generous gift from Dr. Zhen Wang from Xiamen Immocell Biotechnology Co., Ltd.
Authentication	HEK293T cells were obtained from and pre-authenticated by ATCC by STR sequencing and used at low passages. L929 cells and AD293 (Adeno-X 293) cells were obtained from and pre-authenticated by ATCC and Clontech, respectively. Although no detailed authentication information was provided by the suppliers, both strains were used at very low passages in this study (i.e., packaging virus for infecting other cell lines/mice, and examining the efficiencies of sgRNAs used for generating KO mice), and no phenotype was analysed from these two cell lines.
Mycoplasma contamination	The cell lines were routinely tested negative for mycoplasma contamination in our lab.
Commonly misidentified lines (See ICLAC register)	No commonly misidentified lines were used.

Animals and other organisms

Policy information about studies involving animals; ARRIVE guidelines recommended for reporting animal research

Laboratory animals

Mice were housed with free access to water and standard diet (65% carbohydrate, 11% fat, 24% protein) under specific pathogen-free (SPF) conditions. The light was on from 8 a.m. to 8 p.m., with temperature kept at 21-24 °C, and humidity at 40-70%. Male littermate controls were used throughout the study. Metformin was supplied either in drinking water at desired concentrations, or in standard diet at 0.1% (w/w) for 1 week. For creating the diabetic mouse model, mice were fed with HFD (high fat diet, with 60% calories from fat, D12492, Research Diets) for desired time periods starting at 4 weeks old.

AXIN-floxed, LAMTOR1-floxed, AXIN-LKO, and LAMTOR1-LKO mice were generated and maintained as described previously (ref. 24). Wildtype C57BL/6 mice (#000664), DBA2 mice (#000671), and ROSA26-FLPe mice (#016226) were obtained from The Jackson Laboratory. ICR mice (#N000294) were obtained from GemPharmatech. TRPV1-KO mice were obtained from The Jackson Laboratory provided by Dr. David Julius (#003770). TRPV1-KO mice with knockdown of TRPV2-4 or GFP were generated as described previously (ref. 25). APH1A-floxed/APH1B-KO/APH1C-floxed mice were obtained from The Jackson Laboratory provided by Dr. Bart De Strooper (#030985). ATG5-floxed mice were obtained from RIKEN, provided by Dr. Noboru Mizushima (BRC No. RBRC02975). AMPKα1-floxed (Jackson Laboratory, # 014141) and AMPKα2-floxed mice (Jackson Laboratory, # 014142) were obtained from Jackson Laboratory, provided by Dr. Sean Morrison. TG-ALDOA, Tg-ALDOA-D34S, PEN2-floxed and ATP6AP1-floxed mice were generated in this study.

The PEN2-floxed mice were the crossed with Alb-CreERT2 or Villin-CreERT2 mice to generate inducible liver-specific PEN2 knockout mice (PEN2-LKO) or inducible intestine-specific PEN2 knockout mice (PEN2-IKO). The ATP6AP1-floxed mice were crossed with Alb-CreERT2 to generate inducible liver-specific ATP6AP1 knockout mice (ATP6AP1-LKO). AMPKα1/2-floxed mice were crossed with Villin-CreERT2 mice to generate inducible intestine-specific AMPKα knockout mice (AMPKα-IKO). PEN2, ATP6AP1 and AMPKα were deleted by injecting intraperitoneally the mice with tamoxifen (dissolved in corn oil) at 200 mg/kg, 3 times a week. Knockout efficiencies were analysed at 1 week after the last injection by western blotting. ATP6AP1-LKO mice expressing wildtype ATP6AP1 or ATP6AP1Δ420-440 were generated by injection via the tail vein of the different adeno-associated viruses (AAV) carrying indicated inserts before knockout of the endogenous ATP6AP1 by tamoxifen. Levels of the re-introduced ATP6AP1 proteins were analysed at 4 weeks after the virus injection.

The ages of mice used were as follows: a) for isolating primary hepatocytes, normal chow diet-fed wildtype and AMPKα-floxed mice of 4 weeks old (Fig. 1a, b, Extended Data Fig. 1c, e-g, 13g, h, 14f-h, j, n), normal chow diet-fed PEN2-LKO mice of 6 weeks old (Fig. 2b, Extended Data Fig. 3c, j, 14l; into which tamoxifen was injected at 4 weeks old), HFD-fed wildtype mice of 38 weeks old (Extended Data Fig. 12i; fed with HFD for 34 weeks starting from 4 weeks old), HFD-fed PEN2-LKO mice of 38 weeks old (Extended Data Fig. 12i; into which tamoxifen was injected at 35 weeks old after 31 weeks of HFD-treatment starting from 4 weeks old), normal chow diet-fed ATP6AP1-LKO mice expressing full-length ATP6AP1 or ATP6AP1Δ420-440 of 8 weeks old (Extended Data Fig. 14m; into which AAV carrying ATP6AP1 was injected at 4 weeks old, and tamoxifen at 5 weeks old), and HFD-fed ATP6AP1-LKO mice expressing full-length ATP6AP1 or ATP6AP1Δ420-440 of 38 weeks old (Extended Data Fig. 12p; into which AAV was injected at 34 weeks old, and tamoxifen at 35 weeks old, after 34 weeks of HFD-treatment starting from 4 weeks old) were used; b) for GTT, ITT, and measurements metformin, GLP-1, insulin and TAG contents, PEN2-IKO and AMPKα1/2-IKO mice of 6 weeks old (Fig. 4a, b, Extended Data Fig. 12a, b, d, e; mice at 4 weeks old were fed with HFD for a week, and then treated with metformin for another week), HFD-fed PEN2-LKO mice of 54 weeks old (Fig. 4c, d, Extended Data Fig. 12g, h; mice at 4 weeks old were fed with HFD for 31 weeks, and then injected with tamoxifen. At 38 weeks old, mice were treated with metformin for 16 weeks), HFD-fed ATP6AP1-LKO mice expressing full-length ATP6AP1 or ATP6AP1Δ420-440 of 54 weeks old (Fig. 4e, f, Extended Data Fig. 12n, o; mice at 4 weeks old were fed with HFD for 30 weeks, and were injected with AAV at 34 weeks old. At 35 weeks old, the mice were injected with tamoxifen, and then treated with metformin for 16 weeks); c) for PTT, PEN2-LKO mice of 6 weeks old (Extended Data Fig. 14k; mice at 4 weeks old were injected with tamoxifen, and were treated with metformin for a week starting from 5 weeks old); d) for immunoblotting and the measurement of adenylates, wildtype mice of 5 weeks old (Extended Data Fig. 1h-n, 13l; treated with metformin for a week starting from 4 weeks old), PEN2-LKO mice of 6 weeks old (Fig. 4i, Extended Data Fig. 12f; mice at 4 weeks old were injected with tamoxifen, and were treated with metformin for a week starting from 5 weeks old), ATP6AP1-LKO mice expressing full-length ATP6AP1 or ATP6AP1Δ420-440 of 9 weeks old (Fig. 4j, Extended Data Fig. 12m; mice at 4 weeks old were injected with AAV. At 5 weeks old, the mice were injected with tamoxifen, and then treated with metformin for a week starting from 8 weeks old), HFD-fed PEN2-LKO mice of 39 weeks old (Extended Data Fig. 12j; mice at 4 weeks old were fed with HFD for 31 weeks, and then injected with tamoxifen. At 38 weeks old, mice were treated with metformin for a week), HFD-fed ATP6AP1-LKO mice expressing full-length ATP6AP1 or ATP6AP1Δ420-440 39 weeks old (Extended Data Fig. 12q; mice at 4 weeks old were fed with HFD for 30 weeks, and were injected with AAV at 34 weeks old. At 35 weeks old, the mice were injected with tamoxifen, and then treated with metformin for a weeks starting from 38 weeks old), AXIN-LKO mice, LAMTOR1-LKO mice and Tg-ALDOA-D34S of 7 weeks old (Extended Data Fig. 9d, e, 11c, e; treated with metformin for a week starting from 6 weeks old), hepatic ATP6v0c-KD mice of 7 weeks old (Extended Data Fig. 9f; treated with metformin 1-week starting from 6 weeks old, into which AAV carrying siRNA against ATP6v0c was intravenously injected at 4 weeks old), and TRPV1-KO and hepatic TRPV2/3/4-KD mice of 8 weeks old (Extended Data Fig. 11g; treated with metformin 1-week starting from 7 weeks old, into which AAV carrying siRNAs against TRPV2/3/4 was intravenously injected at 5 weeks old); e) for hepatic H&E staining, HFD-fed PEN2-LKO mice of 54 weeks old (Extended Data Fig. 12k; mice at 4 weeks old were fed with HFD for 31

	weeks, and then injected with tamoxifen. At 38 weeks old, mice were treated with metformin for 16 weeks), HFD-fed ATP6AP1-LKO mice expressing full-length ATP6AP1 or ATP6AP1Δ420-440 of 54 weeks old (Extended Data Fig. 12r; mice at 4 weeks old were fed with HFD for 30 weeks, and were injected with AAV at 34 weeks old. At 35 weeks old, the mice were injected with tamoxifen, and then treated with metformin for 16 weeks); and f) for all the other experiments, mice of 4 weeks old were used.<br><br>Worms were maintained on nematode growth medium (NGM) plates [1.7% (w/v) agar, 0.3% (w/v) NaCl, 0.25% (w/v) bacteriological peptone, 1 mM CaCl2, 1 mM MgSO4, 25 mM KH2PO4-K2HPO4, pH 6.0, 0.02% (w/v) streptomycin, and 5 μg/ml cholesterol] spread with E. coli OP50 as standard food. Metformin of desired concentrations was added to the autoclaved NGM (cooled down to 50 °C) before pouring onto pates. All worms were cultured at 20 °C. Wildtype (N2 Bristol), aak-2(ok524), and unc-76(e911) strains were obtained from Caenorhabditis Genetics Center. The ATP6AP1-KO C. elegans strains expressing ATP6AP1 or its Δ420-440 mutant were established in this study.<br><br>The ages of nematodes used in this study were as follows: a) for lifespan assays, worms at L4 stage were used (Fig.4 g, h, Extended Data Fig. 13a, 14d, e; treated with metformin, NAC, etc. until death); b) for analysis of adenylates and pharmacokinetics of metformin, phospho-AMPKα, and ROS levels (Extended Data Fig. 13b, c, e, f, i-k, 14c, i), worms at L4 stage (after treatment of metformin for a day) were used; and c) for the experiments using PEN2-KD worms, worms at L1 stage were used to knocked down of PEN2 (Extended Data Fig. 13d; fed with HT115 E.coli strain containing RNAi against PEN2).
Wild animals	The study did not involve wild animals
Field-collected samples	The study did not involve samples collected from the field.
Ethics oversight	Protocols for all mouse experiments were approved by the Institutional Animal Care and the Animal Committee of Xiamen University (XMULAC20180028).

Note that full information on the approval of the study protocol must also be provided in the manuscript.

Human research participants

Policy information about studies involving human research participants

Population characteristics	Participants were administered with diane-35 (Bayer, Germany), one tablet per day, plus metformin (Bristol-Myers Squibb) at 2000 mg/day. Such a combined treatment lasted 6 months, which was then switched to metformin-alone treatment for another 6 months. At the endpoint of the treatment, six subjects with the lowest BMI were selected for determining pharmacokinetics. A potential selection bias may be introduced. However, they were characterised as follows (mean ± SD): ages 25.5 ± 6.2 years, weight 69.0 ± 6.4 kg, BMI 26.5 ± 1.1 kg/m2, waist circumference 89.8 ± 5.8 cm, hip circumference 102.2 ± 11.4 cm, W/H 0.89 ± 0.12, fasting plasma glucose 5.1 ± 0.7 mM, fasting serum insulin 28.2 ± 15.3 μU/ml, HbA1c 5.1 ± 0.2 %, total cholesterol 5.1 ± 1.0 mM, triglycerides 1.4 ± 0.7 mM, HDL-C 1.4 ± 0.3 mM, LDL-C 3.0 ± 0.6 mM, and were therefore able to represent the general population.
Recruitment	Obese women diagnosed with polycystic ovary syndrome (PCOS) between September 2017 and July 2020 at Shanghai Jiao Tong University Affiliated Sixth People's Hospital were recruited. Subjects included fulfilled the following criteria: a) ages 18 to 40 (inclusive); b) BMI higher than 27.5 kg/m2; and c) meeting the diagnostic criteria of PCOS. The diagnostic criteria of PCOS were as follows: a) irregular period over a year; b) hyperandrogenism; and/or c) ultrasound examination with polycystic ovaries. Subjects with following conditions were excluded: a) after hysterectomy; b) with congenital adrenal hyperplasia, Cushing's syndrome, or androgen secreting tumours within five years; c) mothers pregnant or lactating; d) with thrombosis related history or risk factors, such as deep vein thrombosis, pulmonary embolism, myocardial infarction (angina), valvular heart disease, atrial fibrillation, and cerebrovascular accident (TIA); e) with abnormal liver function (e.g., caused by viral hepatitis); f) with a history of liver malignancy or adenoma, or a history of genital or breast malignancies; g) history of severe or frequent migraine attacks; h) with renal insufficiency; and i) with other factors that may affect the efficacy of the drug or cause complications by the drug.
Ethics oversight	The study was approved by the Ethics Committee of Shanghai Sixth People's hospital and was in accordance with the Helsinki Declaration and Good Clinical Practice. All participants signed an informed consent form before enrollment. This study was registered on ClinicalTrials.gov (ChiCTR-IOR-17013169).

Note that full information on the approval of the study protocol must also be provided in the manuscript.

Clinical data

Policy information about clinical studies

All manuscripts should comply with the ICMJEguidelines for publication of clinical research and a completedCONSORT checklist must be included with all submissions.

Clinical trial registration	The trial was registered with the Chinese Clinical Trial Registry (ChiCTR), with the URL link: http://www.chictr.org.cn/showproj.aspx?proj=21769.
Study protocol	Participants were fasted for 12 h before experiment (started from 10:00 p.m. of previous day), followed by taking orally 0.5 g of Metformin Hydrochloride Extended-release Tablets (Bristol-Myers Squibb) per person. Blood samples were taken at 1, 3, 6 and 12 h after metformin intake, followed by serum preparation.<br>Some 50 μl of serum collected from each mouse or human subject was mixed with 80% methanol (v/v) in water using buformin at 100 μg/l as an internal standard. Aqueous phase was then collected after centrifugation at 15,000g for 15 min at 4 °C. The merformin concentration was then determined by HPLC-MS.
Data collection	Measurement was performed on a QTRAP MS (SCIEX, QTRAP 6500+) connected to a UPLC system (SCIEX, ExionLC AD). Some 2 μl of

each sample was loaded onto a pHILIC column (ZIC-pHILIC, 5 µm, 2.1× 100 mm, PN: 1.50462.0001, Millipore). The mobile phase consisted of 10 mM ammonium formate containing 0.1% formate (v/v) in the LC-MS grade water (mobile phase A) and LC-MS grade acetonitrile containing 0.1% (v/v) formate in LC-MS grade water (mobile phase B) run at a flow rate of 0.3 ml/min. The HPLC gradient was as follows: 95% B held for 1 min, then to 40% B in 6 min, held for 1 min, then to 95% B within 7.5 min, and held for 2.5 min. The QTRAP mass spectrometer was run on a Turbo V ion source and running in negative mode run in a spray voltage of -5,500 V, with source temperature at 500 °C, Gas No.1 at 50 psi, Gas No.2 at 55 psi, and curtain gas at 40 psi. Compounds were measured using the multiple reactions monitoring mode (MRM), and declustering potentials and collision energies were optimised through using analytical standards. The following transitions were used for monitoring each compound: 130/71 for metformin and 158.1/60 for buformin.

Outcomes

The concentration of metformin in each sample was calculated according to the standard curves generated using desired concentrations of metformin.
