# Supplementary material for: Low-dose metformin targets the lysosomal AMPK pathway through PEN2
Source: Nature. 2022 Feb 23;603(7899):159–65. doi: 10.1038/s41586-022-04431-8 (PMC8891018; doi:10.1038/s41586-022-04431-8)
Supplement: Supplementary file 6 — Summary of lifespan analysis for PEN2 and ATP6AP1 worms. [file 41586_2022_4431_MOESM6_ESM.pdf]

Supplementary Table 3 | Summary of life span analysis for PEN2 and ATP6AP1 worms[a,b] Values of mean lifespan and median lifespan, and numbers of worms counted (total, deaths and censored) in each lifespan experiments are shown. See also statistical analysis data (*P* values calculated by Mantel-CoX) comparing the lifespan of worms between different phenotypes treated with metformin.

Genotypes/ treatments	Mean life span (days)			Median life span (days)						P-value Vs saline control within each genotype (Mantel-CoX)
	Estimated life span ± s.e.m.	95% confidence interval		Estimated life span ± s.e.m.	95% confidence interval					
		Lower bound	Upper bound		Lower bound	Upper bound	N[c]	N[d]	N[e]	
**Fig. 4g**										
Ctrl	18.468 ± 0.338	17.805	19.132	18.000 ± 0.590	16.844	19.156	243	57	300	N/A
Ctrl + Met	26.077 ± 0.425	25.243	26.911	26.000 ± 0.417	25.183	26.817	201	49	250	<0.001
si*PEN2*	15.482 ± 0.320	14.854	16.111	16.000 ± 0.298	15.416	16.584	199	51	250	N/A
si*PEN2* + Met	16.382 ± 0.336	15.723	17.041	16.000 ± 0.383	15.249	16.751	198	52	250	0.037
**Fig. 4h**										
FL	21.968 ± 0.484	21.019	22.917	22.000 ± 0.474	21.071	22.929	122	78	200	N/A
FL + Met	29.000 ± 0.529	27.962	30.038	30.000 ± 0.488	29.043	30.957	160	40	200	<0.001
Δ420-440	21.190 ± 0.390	20.425	21.955	22.000 ± 0.488	21.043	22.957	137	63	200	N/A
Δ420-440 + Met	20.923 ± 0.375	20.189	21.657	22.000 ± 0.423	21.171	22.829	163	37	200	0.793
**Extended Data Fig. 13a**										
Saline	19.081 ± 0.466	18.168	19.994	18.000 ± 0.788	16.456	19.544	164	41	205	N/A
10 mM Met	20.284 ± 0.475	19.353	21.215	20.000 ± 0.617	18.790	21.210	166	34	200	0.104
25 mM Met	19.648 ± 0.435	18.796	20.501	20.000 ± 0.515	18.990	21.010	147	53	200	0.636
50 mM Met	25.611 ± 0.530	24.572	26.649	28.000 ± 0.461	27.097	28.903	157	43	200	<0.001
**Extended Data Fig. 14d**										
Saline	18.788 ± 0.415	17.975	19.601	18.000 ± 0.751	16.527	19.473	156	44	200	N/A
Met	26.220 ± 0.450	25.337	27.102	28.000 ± 0.473	27.013	28.927	155	45	200	<0.001
Rotenone	26.989 ± 0.537	25.937	28.041	28.000 ± 0.494	27.032	28.958	157	43	200	<0.001
Met + NAC	18.501 ± 0.401	17.715	19.288	20.000 ± 0.619	18.786	21.214	159	41	200	0.494
Rotenone + NAC	18.490 ± 0.439	17.629	19.531	18.000 ± 0.664	16.698	19.302	159	41	200	0.786
**Extended Data Fig. 14e**										
Saline	14.133 ± 0.313	13.520	14.747	14.000 ± 0.390	13.236	14.764	83	17	100	N/A
Met	13.910 ± 0.279	13.363	14.457	14.000 ± 0.380	13.244	14.756	91	9	100	0.465

[a]Independent repeats of each life span experiment were performed. Data from representative experiments are shown.
[b]Life span data sets within each panel of this table were done in parallel and statistical analyses was done within the data set.
[c]Number of worms scored (death events).
[d]Number of worms censored.
[e]Total number of worms.
